# Nicotinonitriles-based therapeutics as versatile pharmacophores in rational anticancer drug design

**DOI:** 10.1039/d6ra05778k

**Published:** 2026-10-21

**Authors:** Mohamed S. Nafie, Mohamed A. Zeidan, Sherif Ashraf Fahmy, Mohamed K. Diab, Khaled M. Darwish, Asmaa S. A. Yassen

**Affiliations:** a Department of Chemistry, College of Sciences, University of Sharjah Sharjah 27272 United Arab Emirates mohamed.elsayed@sharjah.ac.ae; b Bioinformatics and Functional Genomics Research Group, Research Institute of Sciences and Engineering (RISE), University of Sharjah Sharjah 27272 United Arab Emirates; c Chemistry Department, Faculty of Science, Suez Canal University Ismailia 41522 Egypt; d Department of Pharmaceutical Chemistry, Faculty of Pharmacy, Horus University-Egypt New Damietta 34518 Egypt; e Department of Pharmacy, Institute of Pharmaceutics and Biopharmaceutics, Marburg University Robert-Koch-Str. 4 35037 Marburg Germany sherif.fahmy@pharmazie.uni-marburg.de; f Pest Physiology Department, Plant Protection Research Institute, Agricultural Research Center Giza 12311 Egypt; g Department of Medicinal Chemistry, Faculty of Pharmacy, Galala University New Galala 43713 Egypt asmaa.yassen@gu.edu.eg; h Medicinal Chemistry Department, Faculty of Pharmacy, Suez Canal University Ismailia 41522 Egypt; i Pharmaceutical Organic Chemistry Department, Faculty of Pharmacy, Suez Canal University Ismailia 41522 Egypt

## Abstract

Nicotinonitriles (3-cyanopyridine-based compounds) are emerging as privileged frameworks in rational anticancer drug discovery due to their distinctive electronic properties, metabolic stability, and diverse pharmacological profiles. This review discusses recent developments in nicotinonitrile-derived agents as effective kinase inhibitors of EGFR, VEGFR-2, HER2, CDKs, c-Met, BRAF, and PIM kinases, among others. By discussing mono-, bi-, and multi-target inhibitors, the mechanisms of action are reviewed, with adaptations to induce apoptosis, arrest the cell cycle, and reverse drug resistance. Many clinical and preclinical candidates are nanomolar to sub-micromolar in equipotency across multiple cancer models. The substitution at the core of nicotinonitrile improves targeted selectivity, drug-like properties, and the safety profile, and is a key toward next-generation anticancer agents. Moreover, the review presents information on enzyme-mediated inhibition, hybrid construction, and molecular docking that strengthen the scaffold's potential for ecotoxicological pipelines. Overall, nicotinonitrile-based derivatives seem to be an attractive nucleus for multi-target drug discovery, with a broad therapeutic spectrum in cancer management.

## Abstract 2

Nicotinonitriles (3-cyanopyridine-based compounds) are emerging as privileged frameworks in rational anticancer drug discovery due to their distinctive electronic properties, metabolic stability, and diverse pharmacological profiles.

## Graphical abstract

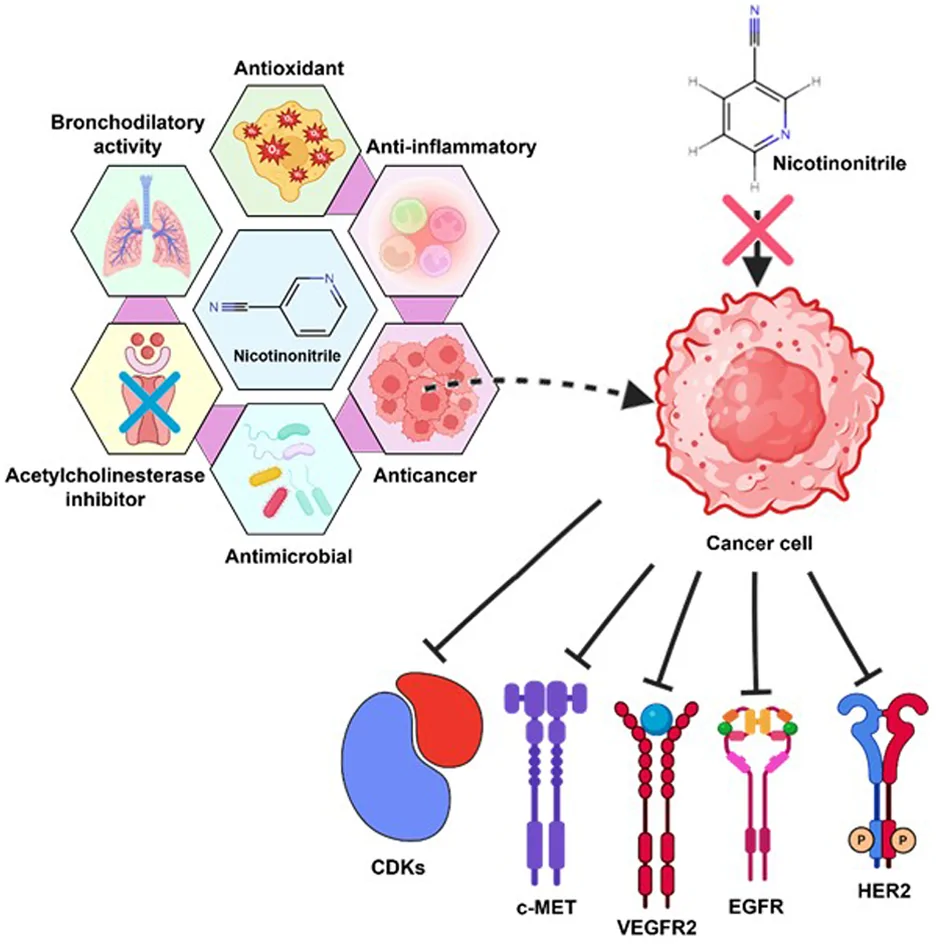

## 1. Introduction

Highly diverse and extensively functionalized nitrogen-containing heterocycles serve as key structural frameworks in numerous natural products and synthetic drugs. These molecules exhibit significant potential in drug discovery and serve as highly valuable functional materials.^1–3^ The pyridine ring system is one of the most common N-heteroaromatics found in a wide range of physiologically active compounds. It is also present in numerous natural compounds such as nicotinic acid, nicotinamide, and vitamin B6, all of which play important roles in metabolism.^4,5^ In recent years, the nitrile group has gained significant importance in medicinal chemistry. Since 2010, the US FDA has approved at least one nitrile-containing drug each year, peaking at 5 approvals in 2020. Due to its distinctive physicochemical characteristics, the nitrile group can enhance a drug's pharmacokinetic and pharmacological performance compared to other compounds with similar therapeutic actions.^6,7^

The 3-cyanopyridine(nicotinonitrile) scaffold has attracted significant interest in recent years due to its versatile pharmacological potential, especially in medicinal chemistry. Its ability to combine with various functional groups to form fused cyanopyridine structures has been shown to yield compounds with a wide range of biological activities. Numerous substituted-3-cyanopyridine derivatives have been synthesized, demonstrating diverse effects, including antioxidant, anti-inflammatory, antiproliferative, antibacterial, acetylcholinesterase-inhibitory, antitubulin, and bronchodilatory activities.^8–10^ Several marketed drugs, including Milrinone, Bosutinib, Neratinib, and Olprinone, are based on nicotinonitrile derivatives Fig. 1.

**Fig. 1:**
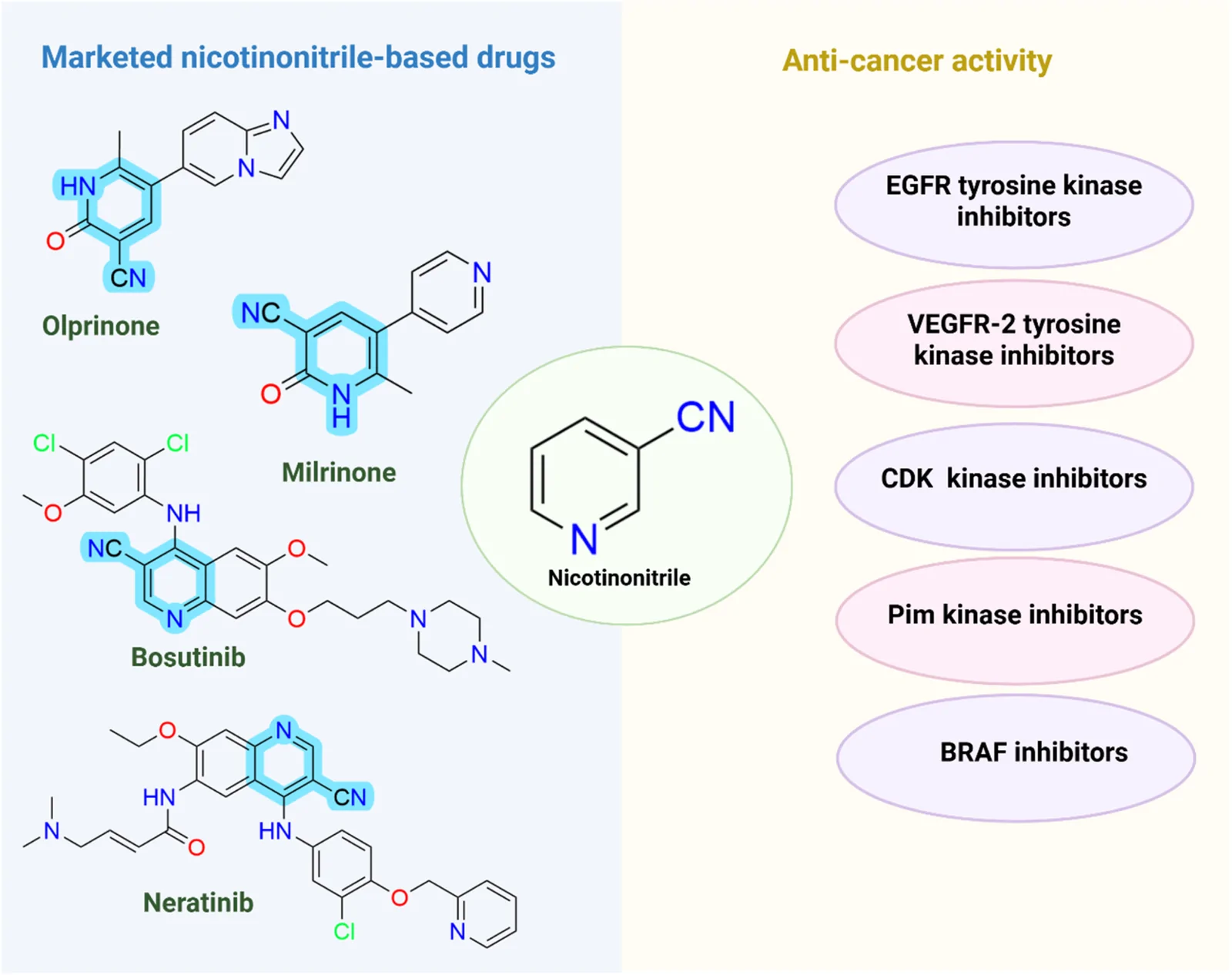
Nicotinonitrile scaffold and representative clinically approved nicotinonitrile-based drugs with their primary molecular targets in cancer therapy.

Bosutinib (Bosulif^®^) and Neratinib (Nerlynx^®^), two potent tyrosine kinase inhibitors, each with Bcr-Abl and HER2/EGFR inhibitory activity, respectively, have received FDA approval and have been indicated in the treatment of chronic myelogenous leukemia as well as HER2-positive breast cancer.^11,12^ Both Milrinone (Primacor^®^) and Olprinone, although designed for cardiovascular use, highlight the pharmacological heterogeneity of this structural template.^13^ TUKYSA^®^ (tucatinib) is a prescription medicine used to treat adults with HER2-positive cancers, including HER2-positive breast cancer and RAS wild-type, HER2-positive colorectal cancer^14^Table 1.

**Table 1:** Nicotinonitrile-standard drugs

Drug	Specificity	Target	References
Bosutinib (Bosulif®)	Philadelphia chromosome-positive chronic myeloid leukemia (Ph + CML)	Targeting and blocking the Bcr-Abl and Src enzymes	15 and 16
Milrinone (Primacor®)	Used for cardiac assistance in cases of acute heart failure, pulmonary hypertension, or chronic heart failure	A phosphodiesterase-3 inhibitor that inhibits the degradation of cyclic adenosine monophosphate (cAMP)	17 and 18
Neratinib (Nerlynx®)	Treating certain types of HER2-positive breast cancer	Kinase inhibitor: binding to and inhibiting HER1 (EGFR), HER2, and HER4	19 and 20
Olprinone	Inotropic, vasodilating, and improving myocardial mechanical efficiency	Phosphodiesterase-3 inhibitor	21 and 22
Tucatinib (TUKYSA®)	Cancer targeting therapy in the treatment of specific types of HER2-positive breast cancer and colorectal cancer	Kinase inhibitor: inhibits the tyrosine kinase enzyme of the HER2 gene	23 and 24

The capability of the nicotinonitrile motif to engage hydrogen-bonding interactions with kinase hinge regions confers its potency and selectivity against targets including EGFR, VEGFR-2, CDKs, c-Met, and PIM-1 kinases.^10–25^ As a result, the 3-cyanopyridine skeleton is adopted as a promising pivotal nucleus for kinase inhibition and apoptosis induction in rational drug design due to the multi-functional therapeutic potential of structurally novel anticancer drugs with targeted higher efficacy, bioavailability, and safety.^25–27^

Nicotinonitriles-based derivatives display a wide range of biological activities.^28^ Anticancer effect is one of the most studied of their wide range of pharmacological activities.^29^ Many nicotinonitrile-based derivatives have been shown to display effective cytotoxic activity against a wide range of cancer cell lines by blocking key oncogenic kinases, including epidermal growth factor receptor (EGFR), vascular endothelial growth factor receptor-2 (VEGFR-2), cyclin-dependent kinases (CDKs), and PIM kinases, consequently inducing apoptosis, suppression of angiogenesis, and arrest in the cell cycle.^25–32^ Some derivatives of them were also reported to exhibit dual- or multi-target inhibition and to reverse drug resistance, thereby improving therapeutic efficacy.^33^ In addition to their role in oncology, nicotinonitrile-based derivatives exhibit other biological activities, including anti-inflammatory, antioxidant, antimicrobial, antitubercular, and acetylcholinesterase-inhibitory effects, as well as bronchodilatory activity, highlighting their pharmacological diversity.^34–38^ Their structural modifiability *via* hybridization or functional replacement enables optimization of physicochemical and pharmacokinetic properties, thereby ensuring their value as lead scaffolds in multi-target drug discovery.^10^

Early pharmacokinetic profiling is critical for the development of successful small molecule drug candidates beyond target potency.^39^ Importantly, parameters such as aqueous solubility, membrane permeability, and overall absorption, distribution, metabolism, excretion and toxicity (ADMET) are vital physiological gatekeepers.^40^ The special structural architecture of the nicotinonitrile scaffold, which exhibits a highly polarized cyano group and an electron-deficient heteroaromatic core, can offer excellent synthetic versatility and structural modifiability. As highlighted in recent studies, functional replacement or hybridization at this nucleus directly can permit the fine-tuning of these physicochemical and pharmacokinetic properties. Furthermore, this approach optimizes the bioavailability, metabolic stability, and safety profile of the scaffold to overcome the low-solubility and rapid-clearance hurdles often encountered in early kinase inhibitor design.^41^ Because most investigational nicotinonitriles in current literature are still at the cell-free biochemical or *in vitro* cell-based preclinical stage, early-stage characterization of their computational and experimental ADMET properties is a crucial translational prerequisite.^42^ This could steer the structural optimization of these nicotinonitriles towards both systemic efficacy and selective *in vivo* tolerability.

Although nicotinonitrile scaffolds have received considerable attention in medicinal chemistry, previous reviews, including those specifically addressing nicotinonitrile derivatives published in 2021 ^9^ and 2023,^10^ have mainly focused on their broad pharmacological applications across multiple therapeutic areas rather than their specific role in anticancer drug discovery. To the best of our knowledge, the present review is the first to provide a cancer-focused, target-oriented analysis of nicotinonitrile-based derivatives, organizing reported compounds according to major molecular targets, including EGFR, VEGFR-2, HER2, CDKs, c-Met, BRAF, PIM kinases, and other cancer-associated targets, with particular emphasis on mono-, dual-, and multi-target inhibition, molecular modeling, apoptosis, and cell-cycle arrest mechanisms.^25–43^ The term nicotinonitrile-based used herein primarily refers to compounds retaining the pyridine-3-carbonitrile framework. The scope is extended to selected substituted, dicarbonitrile-containing, and fused pyridine derivatives when the 3-cyanopyridine motif remains structurally identifiable or constitutes the pharmacophoric basis of the reported anticancer scaffold. Such compounds are more appropriately considered nicotinonitrile-related derivatives rather than strict nicotinonitriles. A literature search was conducted using scientific databases, including ScienceDirect and PubChem, with primary emphasis on studies published between 2020 and 2025, using combinations of keywords such as “nicotinonitrile”, “3-cyanopyridine”, “cyanopyridine”, “anticancer”, “kinase inhibitor”, and specific molecular targets. Thus, this review aims to bridge an important gap in the literature and provide a focused framework for understanding the development of nicotinonitrile-based derivatives as versatile pharmacophores for rational anticancer drug design. Throughout this review, the reported activities of nicotinonitrile-based derivatives are interpreted according to the level of experimental evidence available. Biochemical inhibition of an isolated molecular target is distinguished from antiproliferative or cytotoxic activity in cancer cell lines, while mechanistic cellular studies, such as target phosphorylation, apoptosis, or cell-cycle analyses, are considered a separate level of validation. Cytotoxicity testing in non-malignant cells is discussed as evidence of *in vitro* selectivity rather than systemic safety. Similarly, evidence of antitumor efficacy requires appropriate *in vivo* tumor models, whereas pharmacokinetic properties and systemic toxicity require dedicated animal studies. Claims concerning clinical efficacy or safety are reserved for compounds supported by clinical evidence. This distinction is particularly important because most investigational nicotinonitrile derivatives reviewed herein remain at the biochemical or cell-based preclinical stage.

## 2. Nicotinonitriles molecular targets

The antitumor effect of nicotinonitrile-based derivatives mainly involves multi-targeting and potent inhibitory activity against several key molecular targets, such as EGFR, VEGFR-2, PIM kinases, and other CDKs (CDK2, CDK4, and CDK9) involved in the regulation of the cell division process, transcriptional machinery expression, and apoptosis response in tumor progression.^9–44^ It is important to distinguish biochemical kinase inhibition from cellular target validation. An isolated-enzyme IC_50_, even when accompanied by antiproliferative activity and a favorable docking pose, does not demonstrate that the same kinase mediates the cellular response. Stronger mechanistic support is provided when compound treatment alters kinase phosphorylation or expression, modulates established downstream substrates or signaling pathways, induces a biologically coherent phenotype such as cell-cycle arrest or apoptosis, or produces pharmacodynamic effects *in vivo*. Accordingly, compounds discussed in this review are interpreted according to the depth of vailable mechanistic evidence, and kinase attribution is considered provisional when only biochemical inhibition and molecular modeling are available.^25–45^

### 2.1. Effect of nicotinonitriles on cyclin-dependent kinases' targets

Cyclin-dependent kinases (CDKs) are key enzymes that mediate cell cycle regulation and transcriptional control, making them prime targets in cancer therapeutics.^46^ Of these, CDK2, CDK4, and CDK9 are central to tumorigenesis.^47^ Nicotinonitrile-based molecules have been identified as potent CDK inhibitors due to their planar heteroaromatic cores, which mimic the adenine moiety of ATP and dock favorably in the kinase ATP-binding pocket.^48,49^ CDK2 plays a significant role in the G1/S phase transition by complexing cyclins E and A, as illustrated in Fig. 2.^50^ The G1 phase checkpoint is triggered by the CDK4-cyclin D complex.^51,52^ A fraction of nicotinonitrile-based analogs exhibited high potential for dual CDK2/CDK4 inhibition, which is linked to suppression of retinoblastoma (Rb) phosphorylation and uncontrolled cell proliferation.^53^ CDK9, which is a component of the transcription elongation complex p-TEFb, phosphorylates RNA polymerase II and potentiates transcription of anti-apoptotic genes, including Mcl-1 and Bcl-2.^54,55^ These pathways are chemosensitized when CDK9 is inhibited by nicotinonitrile hybrids, thereby enhancing cancer cell apoptosis.^45^ Taken together, nicotinonitrile scaffolds represent promising moieties for the development of CDK inhibitors that modulate both cell cycle progression and tight transcriptional control, enabling combination or multitarget anticancer strategies.^56^

**Fig. 2:**
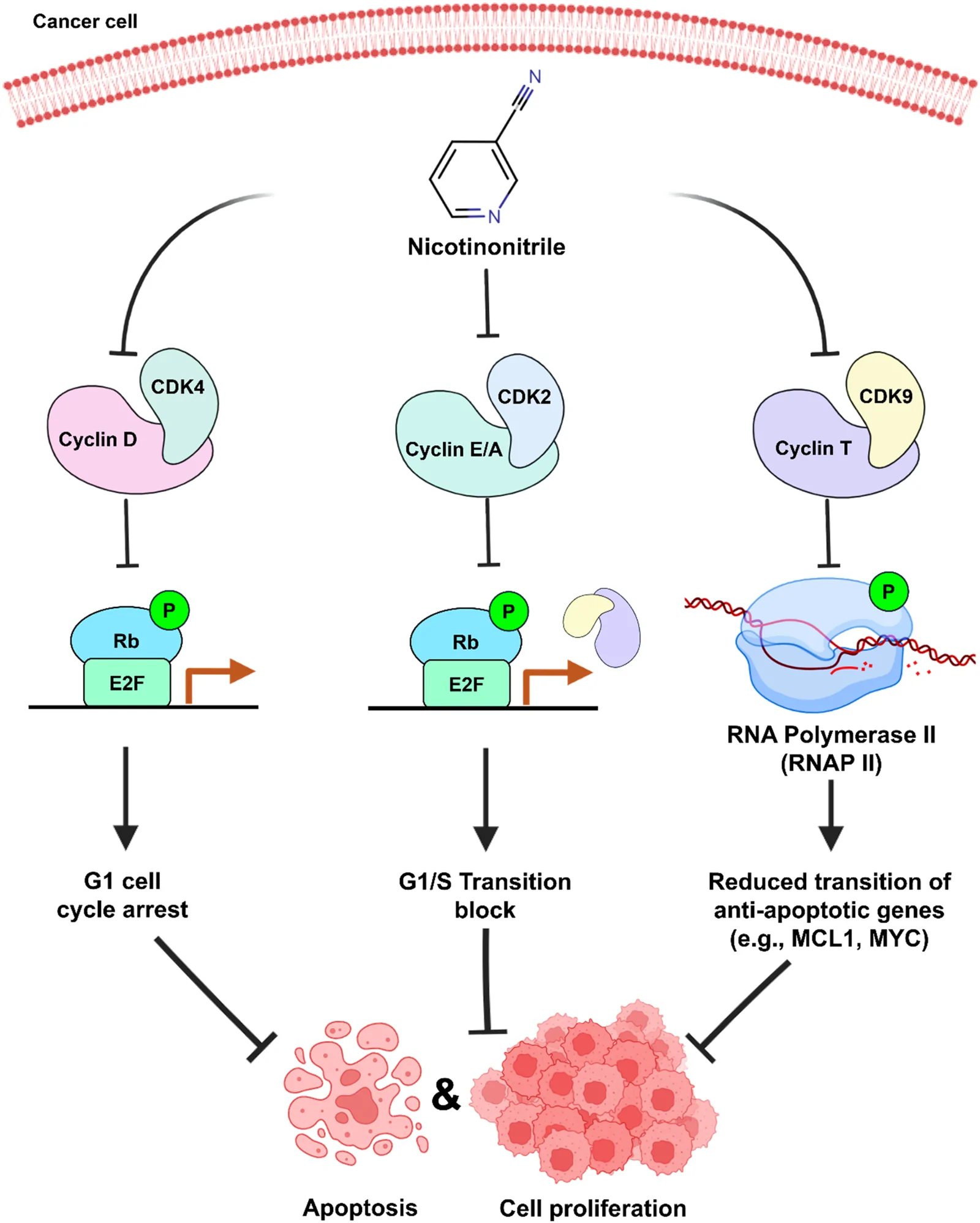
Schematic representation of the proposed multi-CDK inhibitory mechanism of nicotinonitrile and its downstream antiproliferative effects.

### 2.2. Effect of nicotinonitriles on EGFR, VEGFR-2, and HER2 targets

The epidermal growth factor receptor (EGFR), the vascular endothelial growth factor receptor-2 (VEGFR-2), and human epidermal growth factor 2 (HER-2) are key members of the family of receptor tyrosine kinases (RTKs) that regulate proliferation, angiogenesis, and survival. The dysregulation or overexpression of these kinases is associated with many types of cancers, such as lung, breast, and colorectal carcinoma.^43–59^ Nicotinonitrile-based derivatives have gained attention as an attractive small-molecule scaffold for inactivating these receptors, owing to their distinctive structural utility as ATP-like mimics capable of making favorable hydrogen-bonding contacts deep within kinase active sites.^28–33^ Some nicotinonitrile-based derivatives have shown strikingly potent EGFR-inhibiting activity.^31^ These inhibitors function as ATP competitors, as they inhibit the enzyme tyrosine kinase by preventing it from binding to its substrate ATP, which can result in decreased autophosphorylation and diminished signaling through downstream pathways RAS/RAF/MEK/ERK and PI3K/AKT, resulting in apoptotic death of the tumor cell, leading to anti-tumor activity.^60,61^

Dual EGFR-targeting strategies have gained attention for overcoming resistance to EGFR tyrosine kinase inhibitors (TKIs). EGFR drives tumor progression through key downstream pathways, including RAS/RAF/MEK, PI3K/AKT, and JAK/STAT. Resistance may arise through HER2/HER3 or c-Met amplification, as well as bypass activation of RTKs such as VEGFR-2, FGFR, and IGF-1R. In addition, distinct EGFR mutations can reduce sensitivity to first-, second-, and third-generation TKIs. These mechanisms support the development of dual inhibitors that simultaneously block EGFR and compensatory oncogenic pathways to improve therapeutic efficacy and limit acquired resistance.^62^

Similarly, small molecules such as VEGFR-2-targeted nicotinonitrile-based derivatives can inhibit tumorigenesis (one hallmark of tumor progression) by blocking endothelial cell proliferation and migration.^43^ Vascular endothelial growth factor receptor-2 (VEGFR-2), also known as kinase insert domain receptor (KDR), is one of the most well-known targets for cancer treatment. VEGFR2 is the main receptor vascular endothelial growth factor-A (VEGF-A) signals, and it is also a key player in many harmful processes that happen in cancer, going far beyond its usual role in blood vessel growth. The importance of VEGFR-2 in cancer research stems from its diverse roles in tumor progression, encompassing tumor angiogenesis, the preservation of cancer stem cells, epithelial-to-mesenchymal transition (EMT), immune evasion, and metastatic spread, as demonstrated in Fig. 3.

**Fig. 3:**
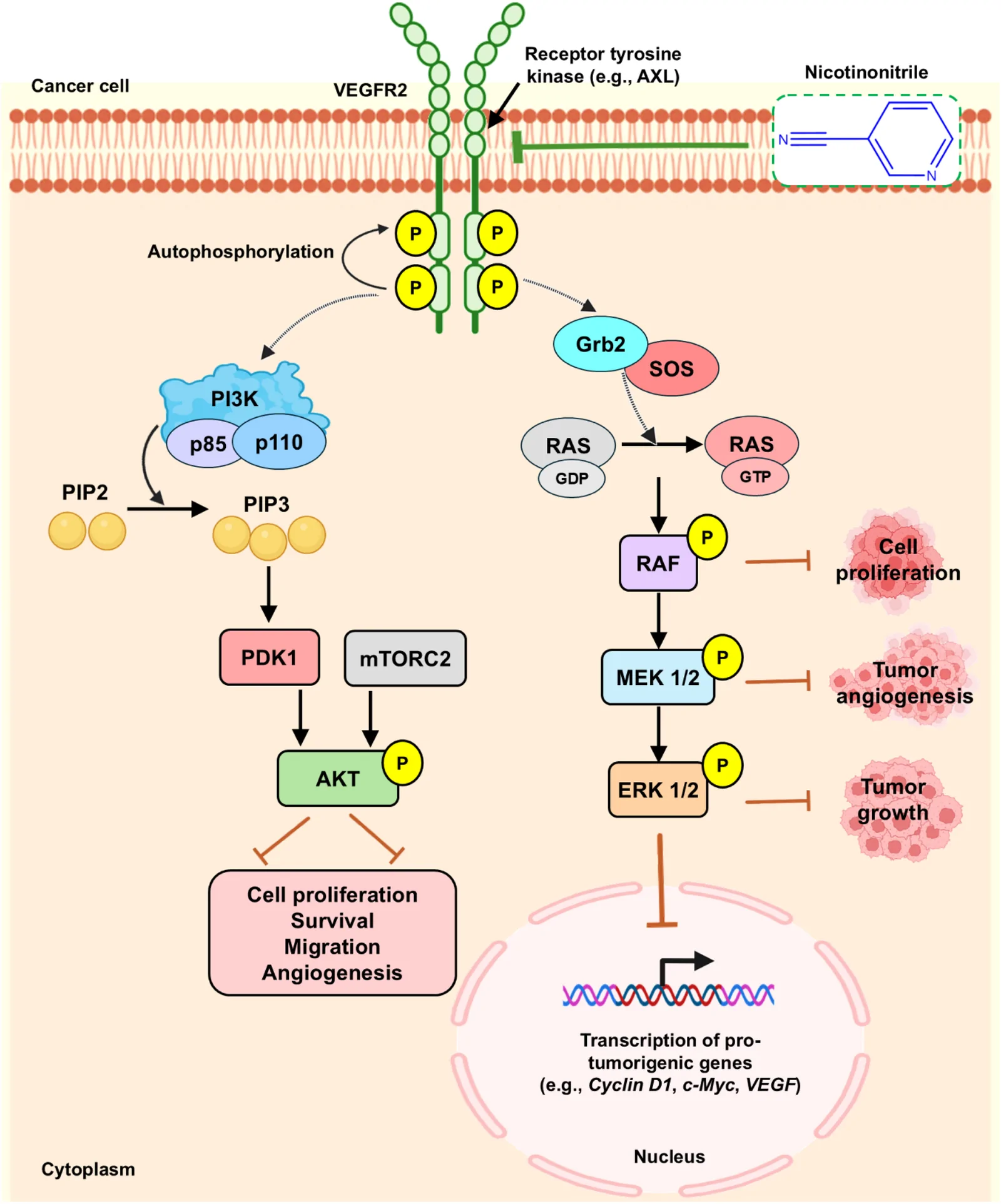
Nicotinonitrile inhibition of the VEGFR-2 pathway in cancer cells. Schematic representation of VEGFR2/AXL-driven PI3K/AKT and RAS/RAF/MEK/ERK signaling pathways regulating tumor cell proliferation, survival, migration, angiogenesis, and growth.

In the case of HER2 inhibition, nicotinonitrile hybrids have shown dual EGFR/HER2 inhibition activity leading to disruption of heterodimerization and downstream mitogenic signaling cascade, which is crucial in therapy for people living with HER-2-positive breast cancer cells.^43^ Several of these dual inhibitors have also displayed potent inhibition of both VEGFR-2 and HER-2 with nanomolar potency, a profile that exemplifies the scaffold's multitarget therapeutic opportunity. In general, nicotinonitrile-based derivatives are structurally malleable scaffolds with the potential to be highly efficacious in targeting multiple oncogenic pathways simultaneously, making them an appealing direction for targeted cancer therapy.^31^

### 2.3. Effect of nicotinonitriles on PIM kinases

The family of proviral integration site for Moloney murine leukemia virus (PIM) kinases, including PIM-1, PIM-2, and PIM-3, are serine/threonine protein kinases implicated in cellular survival signaling pathways such as cell proliferation and apoptosis inhibition.^63^ Amplification of these kinases is a common event in cancer, including prostate, hematologic, liver, and breast cancer, and their downstream effects are orchestrated *via* oncogenic pathways such as JAK/STAT or MYC.^64^ Nicotinonitrile-derived templates have recently been highlighted as potent PIM kinase inhibitors, owing to strong hydrogen-bonding interaction within the ATP-binding pocket and retained high selectivity *via* π–π stacking and hydrophobic interactions.^27–45^ Some other 3-cyanopyridine analogs have been developed as PIM kinase inhibitors with sub-micromolar to nanomolar potency.^65^ These agents induce apoptotic events by suppressing PIM-dependent phosphorylation of BAD and 4E-BP1 and reactivation of intrinsic apoptosis.^30^ In addition, dual-acting derivatives have suppressed PIM-1 and PIM-2, demonstrating improved potency against several cancer cell lines, including HepG2, PC-3, and MCF-7.^30–65^ Although PIM-3 as an anticancer target has not been investigated in detail, several recent nicotinonitrile-based analogues that inhibit all three isoforms are now being developed, potentially exploiting the redundant contribution of PIM signaling, which is often involved in drug resistance.^27^ In summary, nicotinonitrile-based derivatives serve as a versatile scaffold for the development of multi-PIM inhibitors and have great potential for suppressing tumor growth and chemoresistance through pan-PIM kinase inhibition.

PIM kinase signaling cascades are potential targets in multiple tumors and biomarkers across different tumor types. PIM kinase controls the cell cycle by phosphorylating CDK2, p21, NuMA, cdc25A, and cdc25C. Direct stimulation of PIM kinase by the STAT-3/STAT-5 pathway results in cell cycle proliferation and autoregulation *via* phosphorylation of c-Myc. PIM kinase also inhibits 4E-BP1, p70S6K, and H3 and H4 at the level of transcription and cell proliferation. PIM kinases modify the activity of transcription factors c-Myc and c-Myb by phosphorylating their co-factors, including P100, Runx1, Runx2, Notch1, and Notch3. Furthermore, PIM kinase is putatively involved in cellular transcriptional regulation *via* the p65/RelA and Cot-related pathways of NF-kappaB signaling. Cell senescence and apoptosis: caspase 3 is repressed by PIM kinase; phosphorylation of the pro-apoptotic BAD protein decreases phospho-BAD levels; it also attenuates p53 activity to prevent apoptosis. PIM kinase controls cellular senescence *via* CDKN1A, involving multiple pathways such as UHRF1/p16 and MDM2/p53/p21. Cell migratory and invasive activity: eIF4B is phosphorylated by PIM kinase, thereby promoting MET activity, while the CXCR4 receptor responds to PIM kinase-induced cell migration. PIM-2 induces MMP9 expression through Notch1/PI3K/AKT signaling to regulate cell migration. PIM-1 phosphorylates GSK3B and FOXP3, controlling PTGS2 expression to promote cell adhesion/migration/invasion. NKX3.1 in cell invasion is associated with AR and 14-3-3ζ. Translation and Metabolism: PIM kinases control translation by influencing TSC2, 4E-BP1, PRAS40, eIF4B, or LKB1. Up-regulation of PIM-2 promotes the expression of PGC-1α at the protein level and suppresses AMPK activation, suggesting an important role of PIM kinase in energy metabolism control.^25–66^

### 2.4. Effect of nicotinonitriles on c-Met, DHFR, FGFR, and BRAF targets

The nicotinonitrile-based derivatives have strong potency for the ATP-binding pocket in these kinases due to their flat aromatic pyridine and the electron-withdrawing nitrile group, with higher or comparable cytotoxicity toward various cancer cell lines.^33^ Tumor invasion and metastasis are driven by c-Met, a receptor tyrosine kinase that is activated by hepatocyte growth factor (HGF). There are, for instance, nicotinonitrile-derived conjugates that potently inhibited both enzymatic activities of c-Met and PIM-1 and blocked HGF-induced phosphorylation in a noteworthy fashion, as well as modulating metastatic potential in PC-3 and HCT-116 cell lines.^25^ DHFR, on the other hand, is a validated cancer target, and nicotinonitrile-based analogues were able to inhibit this nucleotide biosynthesis enzyme with sub-micromolar potency through hydrogen-bonding interactions with critical catalytic residues that will putatively stop DNA synthesis and cell division, as achieved by methotrexate activity.^67^ For FGFR, nicotinonitrile-based derivatives orient the pyridine nitrogen and the nitrile to form stable interactions within the hinge region, thereby promoting the interruption of oncogenic tumor-angiogenesis-driving FGFR signaling, as represented in Fig. 4.^68,69^ More importantly, BRAF inhibitors possessing 3-cyanopyridine moieties acting as dual EGFR/BRAFV600E suppressors displayed good activity against mutated BRAF-dependent cancers with maintenance of activity on the wild-type isoform.^70,71^ Collectively, these results demonstrate the broad utility of the nicotinonitrile scaffold for multitarget kinase inhibition, with the potential to modulate several oncogenic pathways concurrently, thereby providing a solid basis for new-generation anticancer drugs that overcome resistance.

**Fig. 4:**
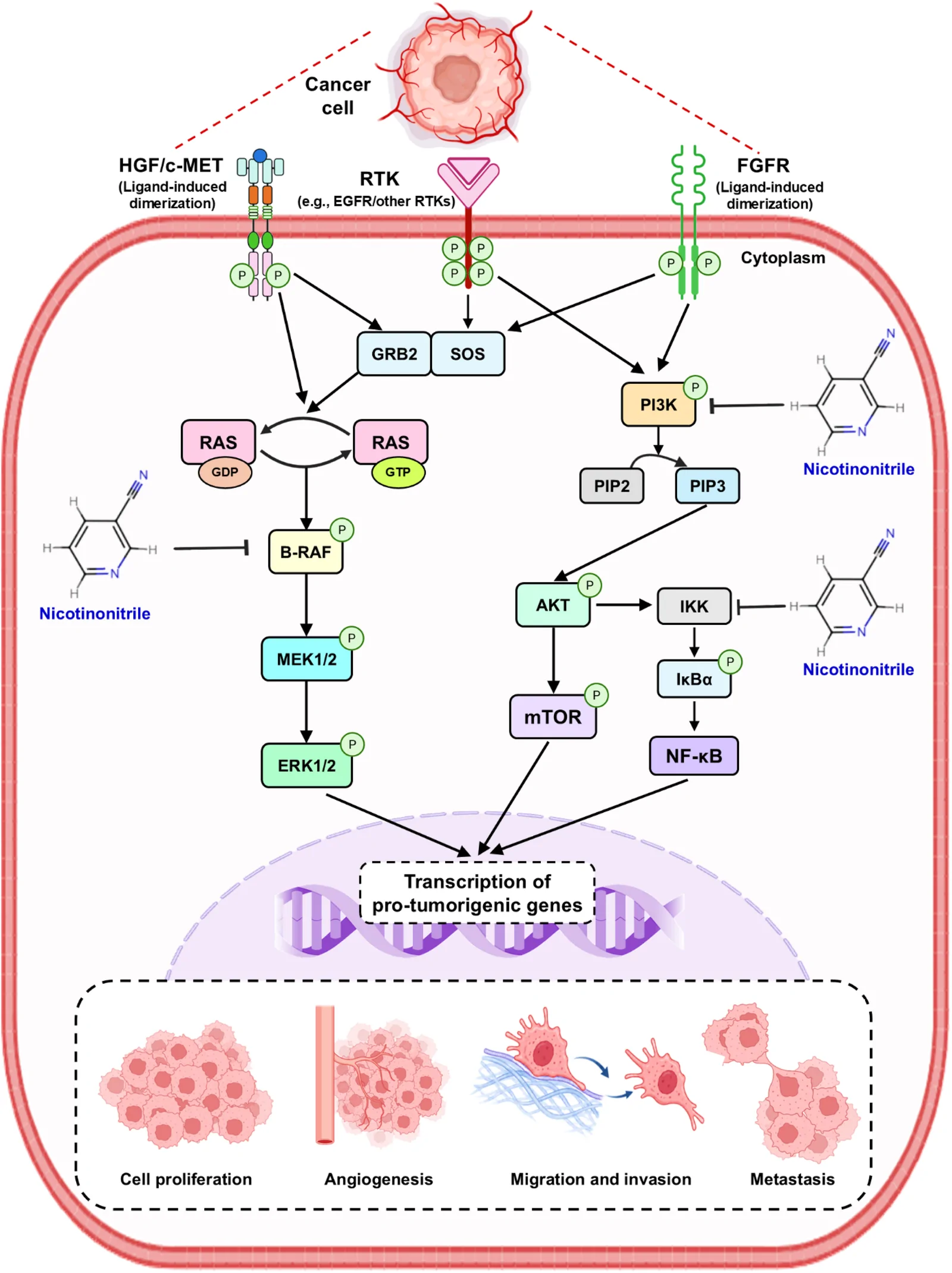
Mechanistic pathways of Nicotinonitrile inhibition for receptor tyrosine kinase (RTK) signaling, including HGF/c-MET, EGFR/other RTKs, and FGFR.

## 3. Enzyme-based nicotinonitrile inhibitors

### 3.1. Single target nicotinonitrile inhibitors

#### 3.1.1. Epidermal growth factor receptor (EGFR) inhibitors

Elsayed H. Eltamany and his group reported the synthesis of novel 3-cyanopyridine compounds as potential EGFR inhibitors. The synthetic route proposed to get the targeted compound 6 is illustrated in Scheme 1. The starting material ethyl 2-[(3-cyano-4,6-dimethylpyridin-2-yl)oxy]acetate 3 was prepared from the substituted 2-pyridine 1*via* the reaction with ethyl 2-chloroacetate 2 in DMF. Refluxing the ester 3 with hydrazine hydrate in EtOH yielded the corresponding hydrazide 4. Benzoylation of the latter 4 with benzoyl chloride 5 in pyridine yielded *N*′-{2-[(3-cyano-4,6-dimethylpyridin-2-yl)oxy]-acetyl}benzohydrazide 6 (yield: 90%).^31^

**Scheme 1:**
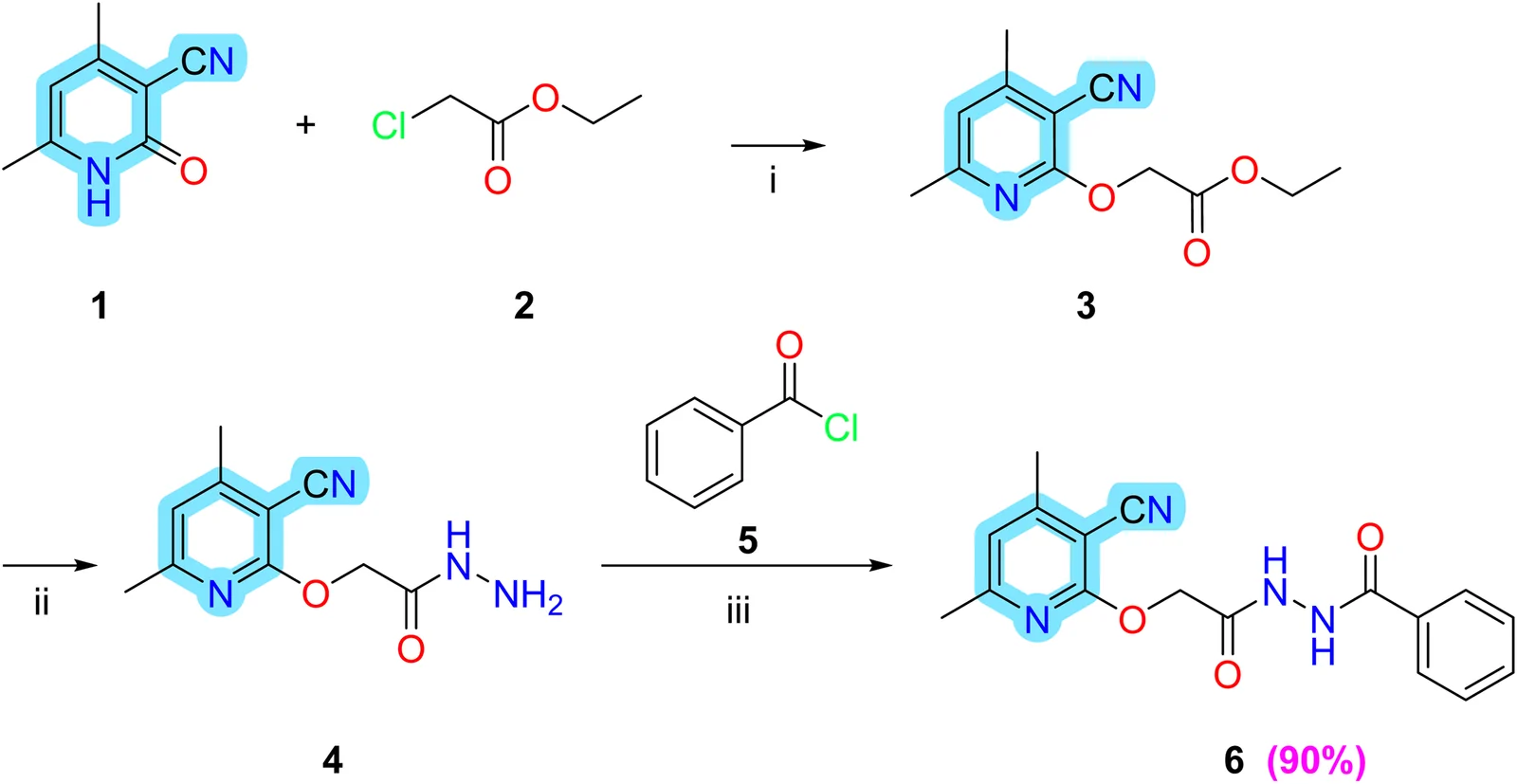
Synthesis of compound 6; reagents and conditions: (i) DMF, K_2_CO_3_, reflux, 8 h; (ii) N_2_H_4_·H_2_O (98%), EtOH, stirring, r.t, overnight; (iii) pyridine, stirring, r.t, overnight.

Compound 6 exhibited potent cytotoxicity against A549 cells with an IC_50_ value of 0.85 µM. It had an IC_50_ value of 3.6 µM against PC-3 cells, comparable to doxorubicin (DOX), which had IC_50_ values 3.07 and 2.23 µM against A549 and PC-3 cells, respectively. 6 showed lower cytotoxicity toward normal WI-38 cells. 6 suppressed EGFR with 96.6% potency and an IC_50_ value of 42.6 nM compared to erlotinib (93.7%, IC_50_ = 78.6 nM). Also, 6 displayed potent cytotoxicity against A549 cells by inhibiting EGFR and inducing apoptosis, and it has the potential to be developed as a chemotherapeutic anti-lung cancer medication.^31^

As potential EGFR inhibitors, Salma Fares *et al.* developed 3-cyanopyridine derivatives containing a benzofuran scaffold. The synthetic route for preparing the target compound 13 is shown in Scheme 2. Knoevenagel condensation of 2-acetyl benzofuran 7 in an ethanolic solution of malononitrile 8 afforded 2-[1-(benzofuran-2-yl)ethylidene]malononitrile 9, which reacted with *N*,*N*-dimethylformamide dimethyl acetal (DMFDMA) 10 to give (*E*)-2-(1-(benzofuran-2-yl)-3-(dimethylamino)allylidene)malononitrile 11. The subsequent condensation reaction of 11 with 2-phenylethanamine 12 yielded 4-(benzofuran-2-yl)-2-(phenethylamino)-nicotinonitrile 13 (yield: 56%).^72^

**Scheme 2:**
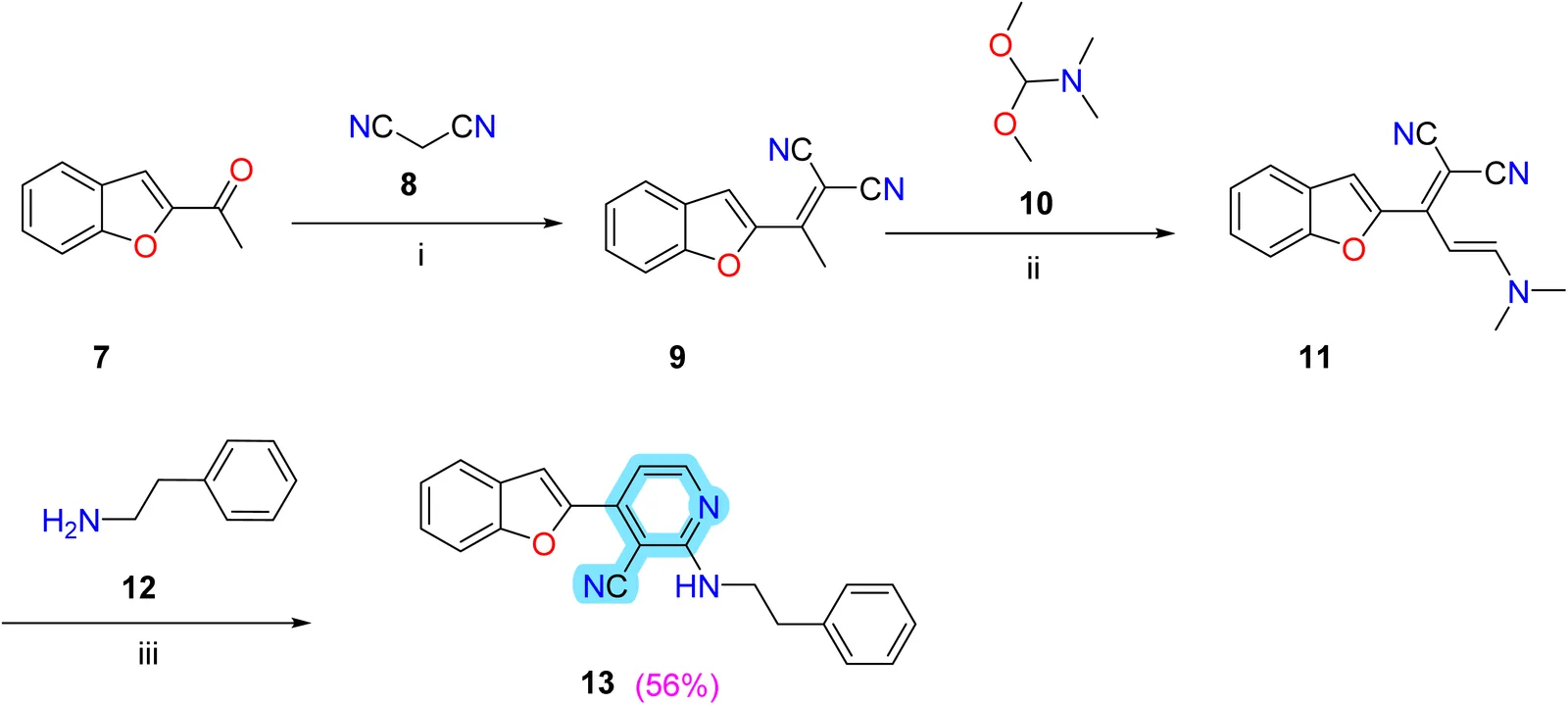
Synthesis of compound 13; reagents and conditions: (i) EtOH, reflux, 24 h; (ii) stirring, r.t, overnight; (iii) reflux, 1h then stirring, overnight.

Compound 13 was tested against five human cancer cell lines: HepG2, HCT-116, MCF-7, PC-3, and HeLa. 13 showed good antiproliferative activity against HePG2, MCF-7, and PC3 cell lines, with IC_50_ values of (20.43, 14.55, and 18.75 µM), respectively, using DOX and Afatinib as positive control. 13 displayed higher inhibitory activity against EGFR (IC_50_ values of 0.81 µM) than the reference drug gefitinib (IC_50_ value of 0.90 µM). The molecular docking analysis of the most active 13 showed a good fit and suitable interactions with key amino acid residues in the EGFR kinase binding sit.^72^

As potential EGFR^WT^ and EGFR^T790M^ inhibitors, M. A. A. Radwan *et al.* reported the synthesis and biological evaluation of 3-cyanopyridine derivatives. The synthesis of targeted compound 19 was achieved in two main steps, Scheme 3. The first step involved the preparation of 1-(furan-2-yl)-3-(thiophen-2-yl)chalcone 17 by the Claisen–Schmidt reaction of 2-acetylfuran 15 with 2-thiophenecarboxaldehyde 14 in the presence of catalytic KOH.^73^ Then, cyclisation of chalcone 17 with ethyl cyanoacetate 18 afforded the desired pyridine 19 (yield: 60%).^74^

**Scheme 3:**
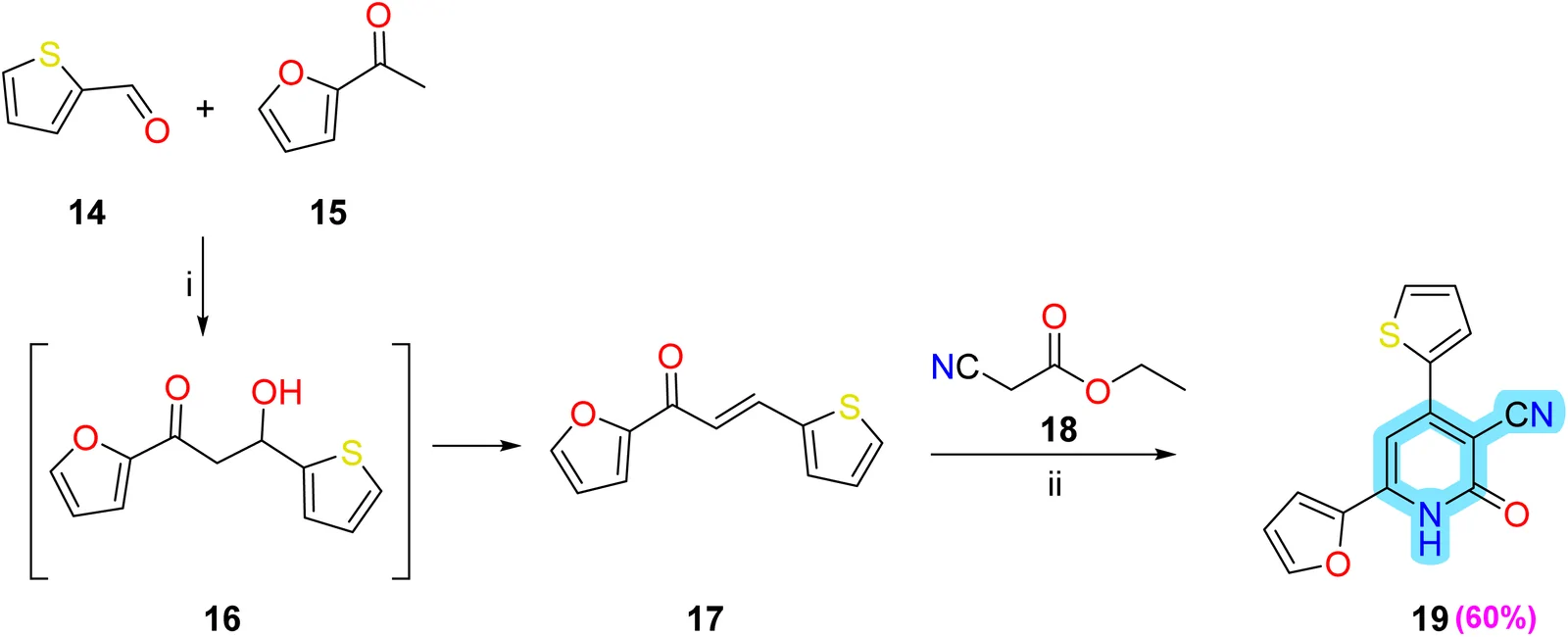
Synthesis of compound 19; reagents and conditions: (i) KOH, EtOH, stirring, 5 h; (ii) AcONH_4_, butanol, reflux, 5 h.

Compound 19 demonstrated significant cytotoxic activities with IC_50_ values of 3.7 µg mL^−1^, 3.8 µg mL^−1^, 4.7 µg mL^−1^, 3.9 µg mL^−1^, 4.1 µg mL^−1^, 4.7 µg mL^−1^, 3.9 µg mL^−1^, and 4.6 µg mL^−1^ against MCF-7, HepG2, HEP2, HCT116, Caco, T47D, FaDu and VERO cell lines compared to DOX as the reference drug. 19 also displayed inhibitory activities against both EGFR^WT^ and EGFR^T790M^ with IC_50_ values of 0.203 and 0.156 µM, respectively, than erlotinib (IC_50_ = 0.042 and 0.009 µM), respectively. Moreover, the molecular docking study demonstrated the outstanding anticancer activity of the investigated derivative by revealing its binding interactions with the EGF receptor.^74^

Fayza O. Ashmawy and her team reported the synthesis and *in vitro* evaluation of novel thiophenyl thiazolyl–pyridine hybrids as potential anticancer agents. The synthesis of targeted derivative 26 was carried out according to the synthetic pathways described in Scheme 4. To synthesize the key intermediate 24, firstly, condensation of ketone 20 with thiosemicarbazide 21 to afford thiosemicarbazones, Schiff's base, 22. The second step involved cyclizing the latter 22 in the same vessel with 3-chloropentane-2,4-dione 23. Finally, one-pot synthesis of compound 26*via* the reaction of intermediate 24, 4-chlorobenzaldehyde 25, and malononitrile 8 under reflux in the presence of ammonium acetate in glacial acetic acid led to the formation of the target 26 (yield: 84%).^75^

**Scheme 4:**
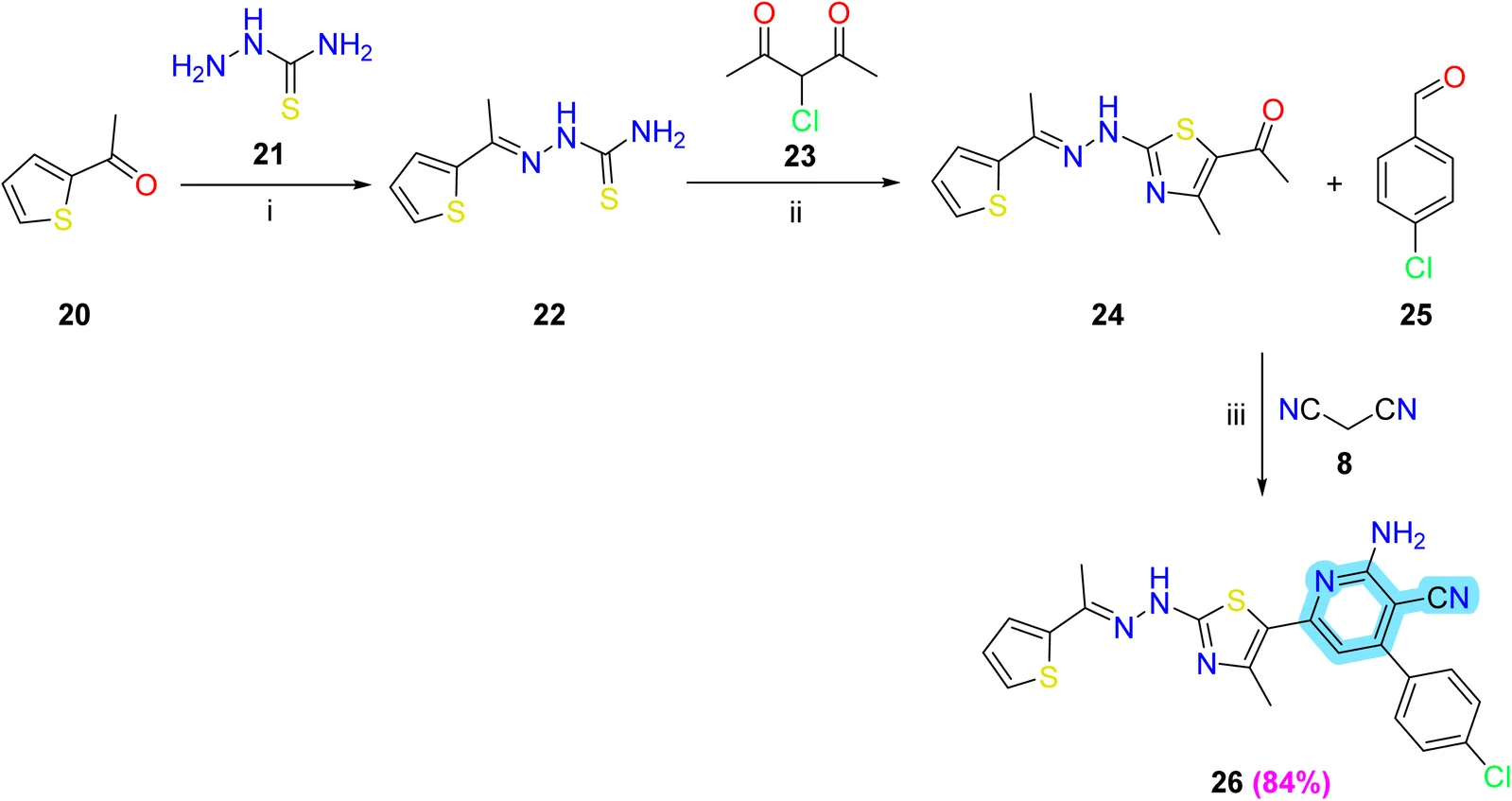
Synthesis of compound 26; reagents and conditions: (i) EtOH, HCl (cat.), reflux, 4 h; (ii) EtOH, reflux, 6 h; (iii) AcONH_4_, gl. AcOH, reflux, 4 h.

Compound 26 was investigated for its *in vitro* anticancer activity against A549 cell line. It showed promising cytotoxicity with an IC_50_ of 0.302 µM, compared to DOX (IC_50_ of 0.460 µM). The ability of 26 to interact with EGFR active sites and to inhibit its activity was also confirmed by docking studies.^75^

Amr S. Abouzied and his team reported the synthesis of some novel 1,3,4-thiadiazole derivatives bearing 3-cyanopyridine moiety. The synthesis of the target compound 31 is described in Scheme 5. 1-(3-Cyano-4,6-dimethyl-2-oxopyridin-1(2*H*)-yl)-3-phenylthiourea 29 was synthesized through the reaction of phenyl isothiocyanate 28 with 1-amino-4,6-dimethyl-2-oxo-1,2-dihydropyridine-3-carbonitrile 27 under the influence of a catalytic amount of KOH in DMF. Further, compound 29 was treated with hydrazonoyl chloride 30 in DMF in the presence of triethylamine (TEA), which afforded the desired product 31 (yield: 86%).^76^

**Scheme 5:**
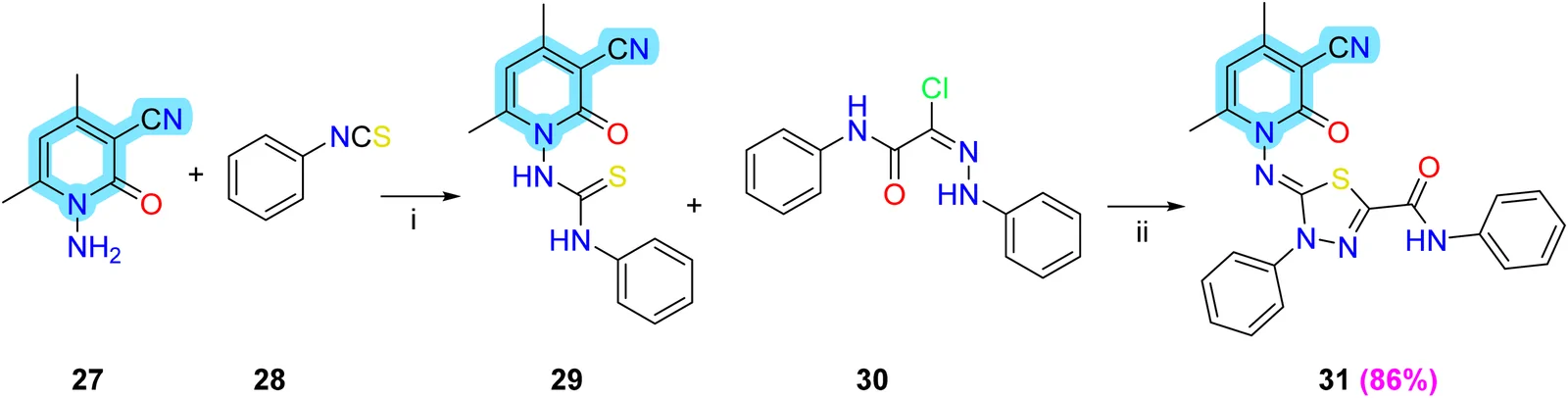
Synthesis of compound 31; reagents and conditions: (i) DMF, KOH, stirring, r.t, 8 h, (ii) DMF, Et_3_N, reflux, 3–6 h.

Compound 31 showed effectiveness towards the inhibition of HCT-116 and HepG2 cell lines with IC_50_ values of 2.03 ± 0.72 and 2.17 ± 0.83 µM, respectively, compared with Harmine as a reference (IC_50_ = 2.40 ± 0.12 and 2.54 ± 0.82 µM, respectively). Molecular docking studies were performed that established a binding site with EGFR TK. 31 showed the best binding affinity.^76^

Hanan A. Soliman *et al.* reported the synthesis and biological evaluation of 6-cyclohexyl-2-(2-oxopropoxy)nicotinonitriles. The target compound 36 was synthesized following the procedures in Scheme 6. The key starting material 34 was synthesized *via* one-pot multi-component reaction of benzaldehyde 33 with methyl cyclohexyl ketone 32, ethyl cyanoacetate 18, and ammonium acetate in ethanol. Finally, 3-cyano-2-pyridone derivative 34 was stirred with chloroacetone 35 in the presence of anhydrous K_2_CO_3_ in dry DMF, yielding the desired product 36 (yield: 88%).^44^

**Scheme 6:**
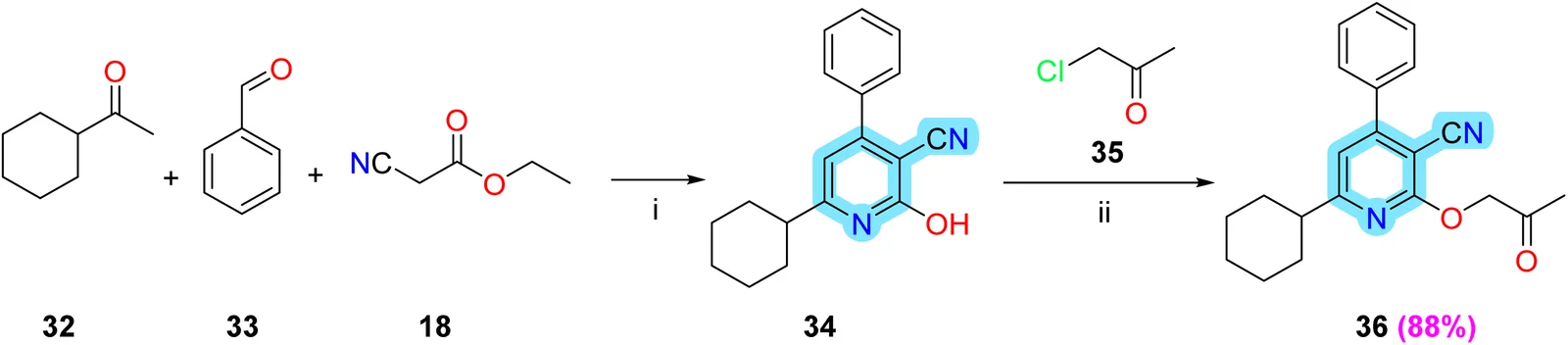
Synthesis of compound 36; reagents and conditions: (i) AcONH_4_, EtOH, reflux, 3–5 h, (ii) DMF, K_2_CO_3_, stirring, r.t, overnight.

Compound 36 exhibited notable cytotoxicity against cancer cell lines (A549 and PACA2) with IC_50_ values of 7.3 µM and 29.8 µM, respectively, compared to DOX, the reference drug (IC_50_ = 49.3 and 48.8 µM, respectively). Docking studies revealed a stable complex and strong binding affinity of 36 to EGFR.^44^

#### 3.1.2. Vascular epidermal growth factor receptor-2 (VEGFR-2) inhibitors

Mohamed G. Abouelenein and his colleagues reported the synthesis and anticancer assessment of novel polyfunctionalized 3-cyanopyridine congeners. The synthetic strategy deliberated for the synthesis of the target compounds 40 and 44 is illustrated in Scheme 7. A one-pot multi-component reaction was adopted to easily synthesize the key intermediate 38 by treating an ethanolic solution of 4-chlorobenzaldehyde 25 and 4-methylacetophenone 37 with an excess of malononitrile 8 and ammonium acetate. Condensation of d-xylose 39 with *O*-aminonitrile 38 in boiling acetic acid afforded the Schiff base 40 (yield: 83%). On the other hand, refluxing *O*-aminonitrile 38 with furfural 41 provided the *N*-condensation adduct, Schiff base 42, which underwent cyclization with chloroacetyl chloride 43, yielding 4-oxoazetidin-1-yl-nicotinonitrile derivative 44 (yield: 78%).^77^

**Scheme 7:**
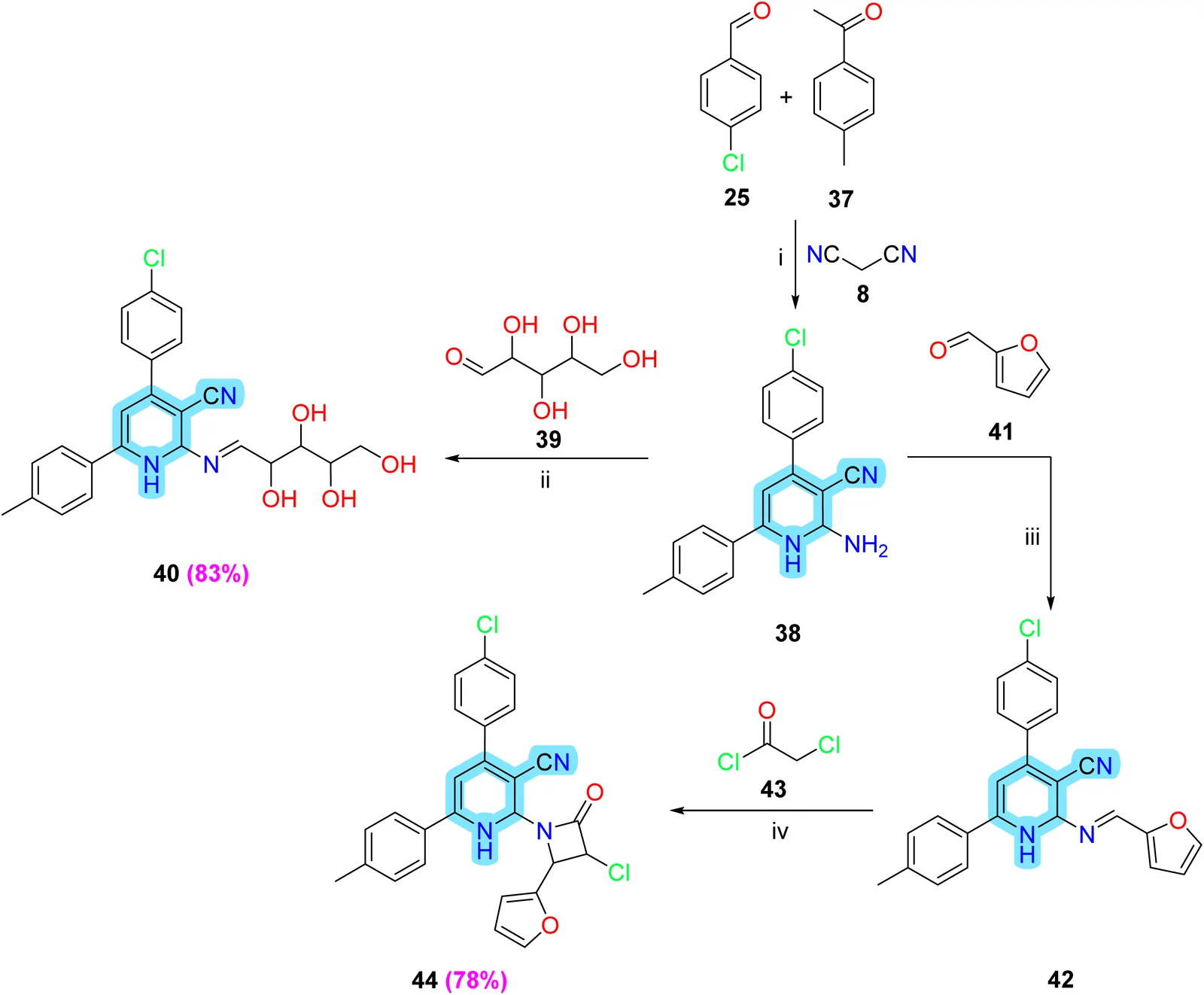
Synthesis of compounds 40 and 44; reagents and conditions: (i) AcONH_4_, EtOH, M.W, 6 min; (ii) AcOH, reflux, 8 h; (iii) EtOH, gl. AcOH, reflux, 8 h; (iv) dioxane, TEA, stirring, 0–5 °C, 10–12 h.

The antiproliferative activity of compounds 40 and 44 was evaluated *in vitro* against three human cancer cell lines; HCT-116, HepG-2, and MCF-7, and DOX was used as a reference drug. Compound 40 displayed significant cytotoxicity against the three cell lines with IC_50_ values of 4.1 µM, 3.6 µM, and 4.0 µM, respectively. Additionally, 44 showed a higher cytotoxic effect against MCF-7, with an IC_50_ of 3.9 µM, compared to 40 and the respective control. The ability of these novel derivatives to interact with EGFR active sites and to inhibit its activity was also confirmed by docking studies united with molecular dynamics simulation.^77^

Amal AbdelHaleema and her team reported the synthesis of novel 3-cyano-6-naphthylpyridine scaffold-based derivatives as selective VEGFR-2 inhibitors and cytotoxic agents. The general synthetic pathway adopted for the synthesis of the novel 3-cyano-6-naphthylpyridine derivative 49 is illustrated in Scheme 8. The synthesis started with the preparation of 4-aryl-3-cyano-6-(2-naphthyl)pyridine-2-one 47*via* one-pot multi-component reaction. Whereby, a mixture of 2-acetyl naphthalene 45, 4-hydroxy-3-methoxy benzaldehyde 46, ethyl cyanoacetate 18, and excess ammonium acetate was reacted in refluxing absolute ethanol. Subsequent chlorination of the pyridine-2-one derivative 47 was done by refluxing in phosphorous oxychloride, utilizing a catalytic amount of phosphorous pentachloride, yielding the 2-chloro pyridine derivative 48. Finally, reflux of the latter 48 with hydrazine hydrate in absolute ethanol afforded the target 2-hydrazino pyridine derivative 49 (yield: 59%).^78^

**Scheme 8:**
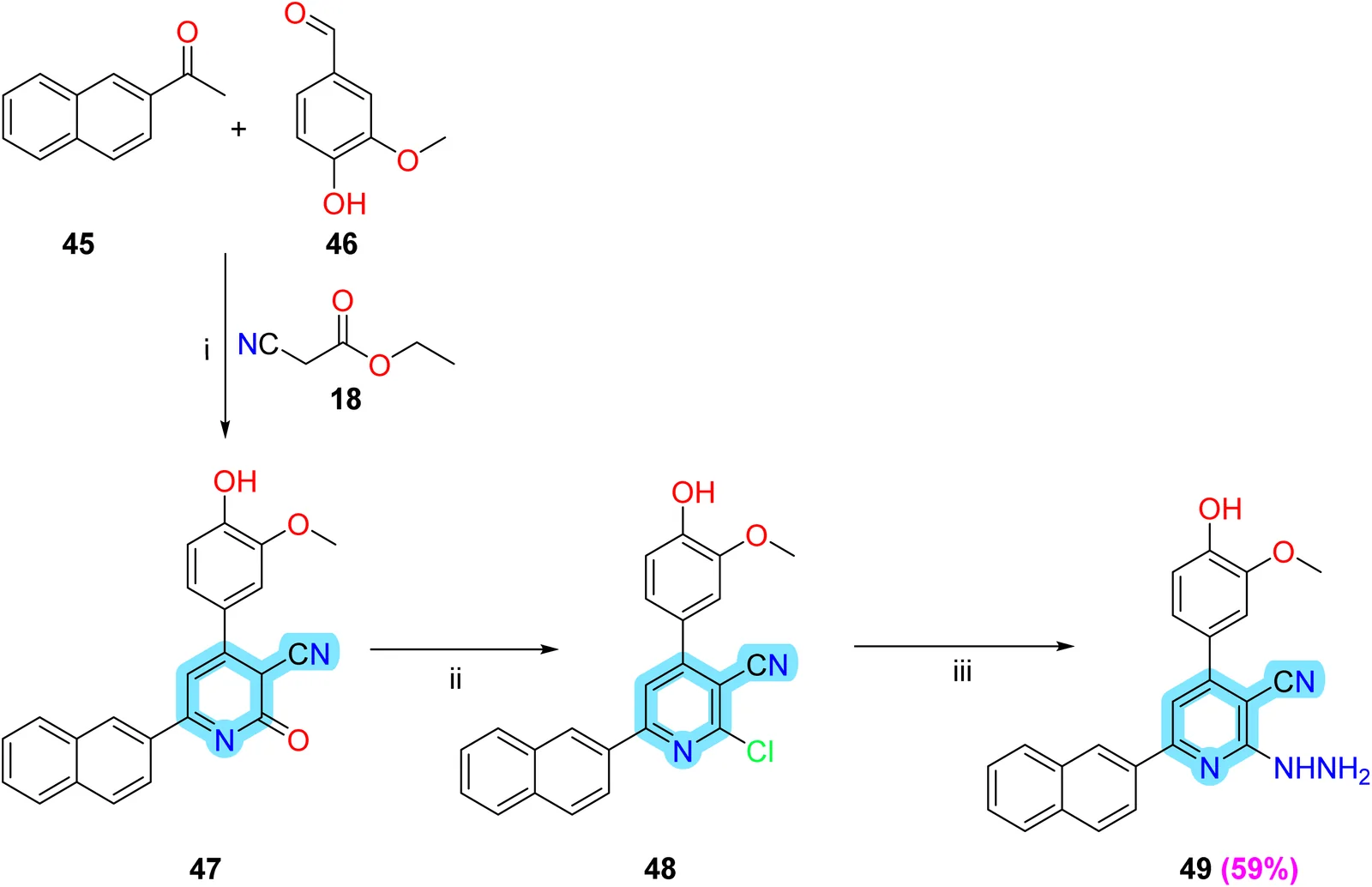
Synthesis of compound 49; reagents and conditions: (i) ACONH_4_, EtOH, reflux, 8 h; (ii) POCI_3_, PCl_5_, reflux, 5 h; (iii) NH_2_NH_2_·H_2_O, EtOH, reflux, 4 h.

Compound 49 was tested *in vitro* against two prostate cancer cell lines, PC-3 and DU145 and two breast cancer cell lines, MCF-7 and MDA-MB435. 49 showed the most potent cytotoxic activity against PC-3 and MCF-7, with IC_50_ values of 55 µM and 0.5 µM, respectively, compared to the standard DOX (IC_50_ = 59 µM and 11 µM, respectively). Also, 49 was found to be the most potent VEGFR-2 inhibitor among all the tested derivatives (IC_50_ = 0.19 nM), compared with delphinidin as the standard (IC_50_ = 5.09 nM). Docking studies showed that 49 recognized the ATP-binding site of the VEGFR-2 kinase and interacted with it.^78^

Nashwa M. Saleh and her researchers developed the design and synthesis of pyridine-derived VEGFR-2 inhibitors. The synthetic strategy for achieving the target compound 55 is shown in Scheme 9. The synthesis of Schiff's base 52 as a key intermediate was carried out by condensation of equimolar amounts of *O*-bromobenzaldehyde 50 with 2-cyanoacetohydrazide 51 in absolute ethanol with a few drops of glacial acetic acid. Further, the targeted 1,6-diamino-4-(2-bromophenyl)-2-oxo-1,2-dihydropyridine-3,5-dicarbonitrile 53 was synthesized by the reaction of 52 with malononitrile 8 in the presence of piperidine. Reaction of the product 53 with benzaldehyde 33 afforded the desired 54. Finally, the [1,2,4]triazolo[1,5-*a*]pyridine derivative 55 was obtained by stirring 54 with trifluoroacetic acid (yield: 35.7%).^79^

**Scheme 9:**
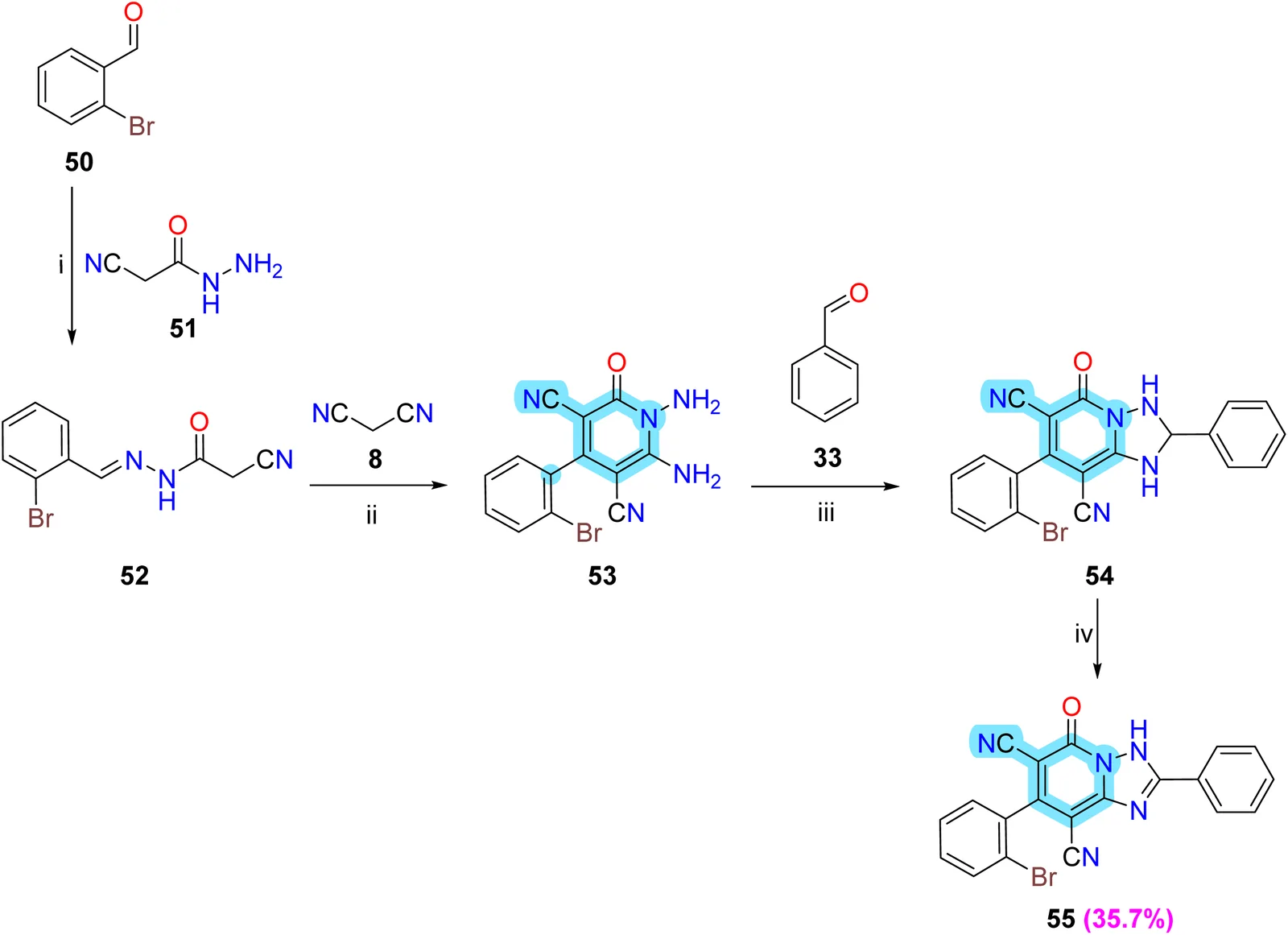
Synthesis of compound 55; reagents and conditions: (i) EtOH, gl. AcOH, stirring, r.t, 3 h; (ii) EtOH, piperidine, reflux, 5 h; (iii) dioxane, piperidine, reflux, 5 h; (iv) CF_3_COOH, stirring, r.t, 5 min.

Compound 55 was tested for its anticancer activity against two human tumor cell lines: HepG2 and MCF-7. 55 showed more potent cytotoxic activity against HepG2 and MCF-7, with IC_50_ = 4.25 and 6.08 µM, respectively, compared to sorafenib (IC_50_ = 9.18 and 5.47 µM, respectively) and DOX (IC_50_ = 7.94 and 8.07 µM, respectively). Moreover, 55 inhibited VEGFR-2 with an IC_50_ value of 0.12 ± 0.01 µM, like sorafenib's IC_50_ value of 0.10 ± 0.02 µM, making it the most effective derivative.^79^

Marwa F. Ahmed *et al.* reported the discovery of pyridine-sulfonamide hybrids as a new scaffold for the development of potential VEGFR-2 inhibitors. The synthetic pathway used for the preparation of the desired derivative 61 is represented in Scheme 10. The synthesis started with the reaction of *p*-toluenesulfonyl chloride 56 with 4-aminoacetophenone 57, affording the desired sulphonamide 58. Then, the Claisen–Schmidt condensation of sulphonamide derivative 58 with 3-fluorobenzaldehyde 59 in ethanolic sodium hydroxide solution gave the chalcone 60. Finally, the reaction of the latter chalcone 60 with malononitrile 8 in the presence of ammonium acetate afforded the desired pyridine derivative 61 (yield: 71%).^80^

**Scheme 10:**
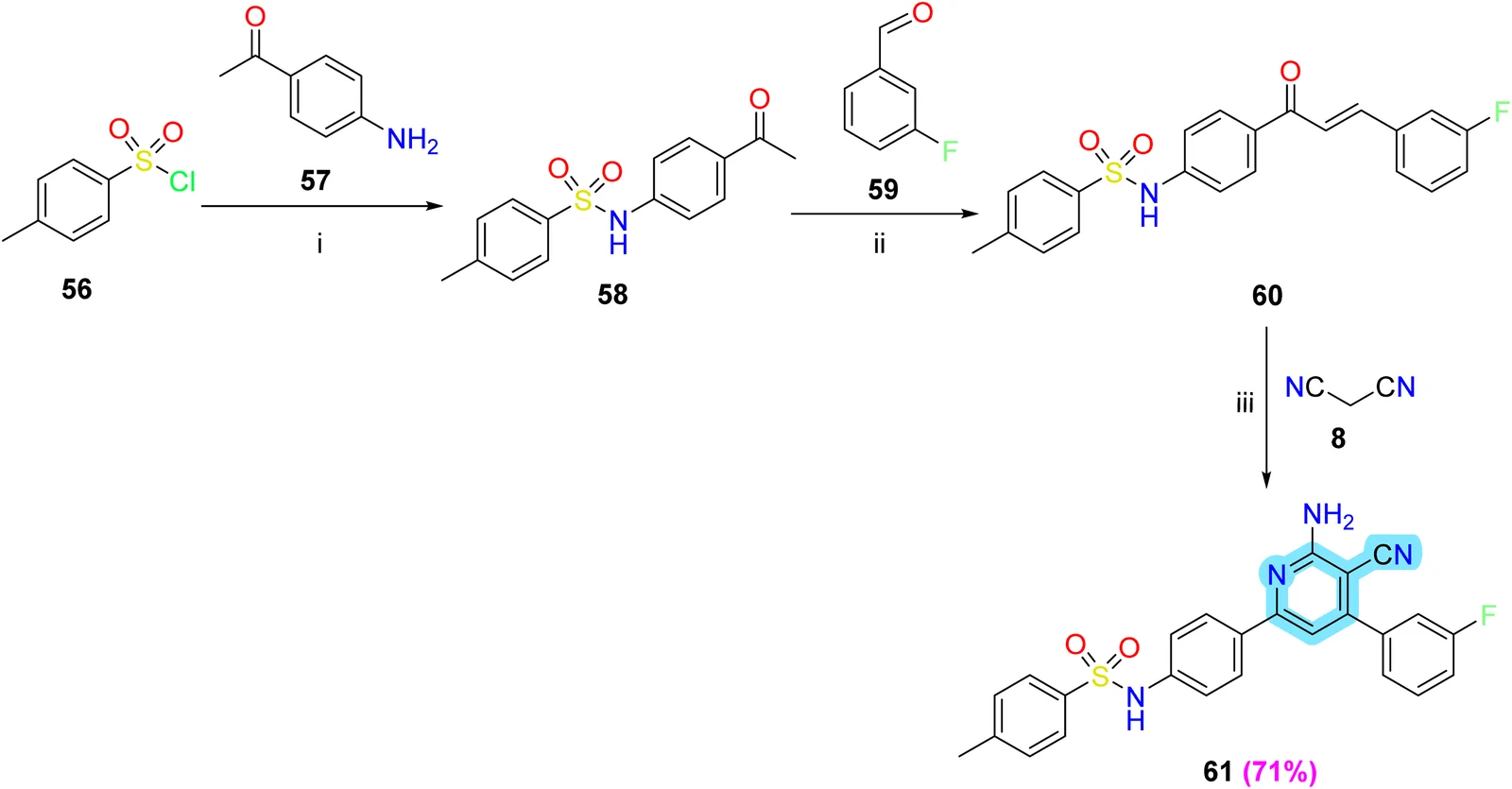
Synthesis of compound 61; reagents and conditions: (i) pyridine, reflux, 2 h; (ii) Alc. NaOH (40%), stirring, r.t, 5 h; (iii) AcONH_4_, EtOH, reflux, 12 h.

Compound 61 exhibited potent antiproliferative activity, with GI_50_ values ranging from 1.06 to 8.92 µM against most tested cancer cell lines. 61 showed a mean inhibition value of 91.67%. Furthermore, it showed inhibition percent more than 100% on leukemia (HL-60(TB)), (RPMI-8226), non-small cell lung cancer (HOP-92, NCI- H522), colon cancer (HCT-116, HCT-15 HT29, SW-620), CNS cancer (SNB-75), melanoma (M14, SK-MEL-28, UACC-62), ovarian cancer (OVCAR-3, OVCAR-4, IGROV1), breast cancer (T-47D, MDA-MB-468) and renal cancer (ACHN, RXF 393, TK-10, UO-31). Furthermore, 61 was tested for its enzyme inhibition activity against VEGFR-2, where VEGFR-2 was potently inhibited, with an IC_50_ of 3.6 µM, compared with sorafenib (IC_50_ = 4.8 µM).^80^

#### 3.1.3. Cyclin dependent kinase (CDK) inhibitors

Adel A.-H. Abdel-Rahman *et al.* reported the discovery of new 3-cyanopyridine derivatives as CDK2 inhibitors. For preparing the target hybrids 63 and 65, the synthetic route is clarified in Scheme 11. Chlorination of compound 62 with phosphorous oxychloride afforded the desired 63. On the other hand, 65 was obtained by reacting 64 with chloroacetyl chloride 43 in acetone and K_2_CO_3_ at room temperature (yield: 89%).^48^

**Scheme 11:**
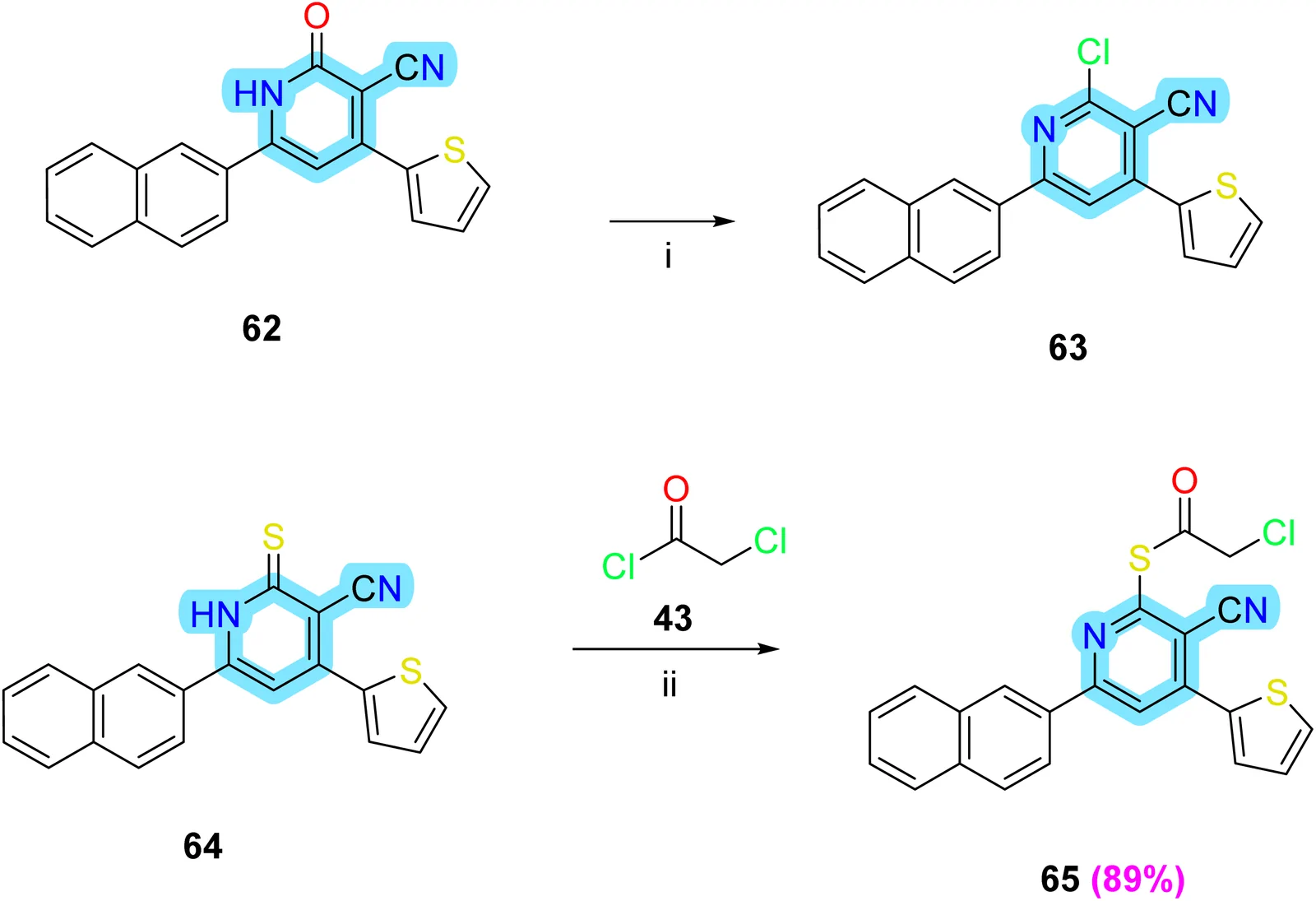
Synthesis of compounds 63 and 65; reagents and conditions: (i) POCl_3_, reflux, 24 h; (ii) K_2_CO_3_, acetone, stirring, r.t, 8 h.

Compounds 63 and 65 were tested *in vitro* for their activity against HCT-116, MCF-7, HepG2, and A549 human cancer cells. 63 exhibited cytotoxicity with IC_50_ values of 45.9, 46.2, 42.9, and 67.2 µM, respectively, while 65 exhibited cytotoxic activity with IC_50_ values of 31.7, 46.4, 27.6, and 56.9 µM, respectively, compared to DOX (IC_50_ = 40.0, 64.8, 24.7, and 58.1 µM, respectively). Compounds 63 and 65 were evaluated for their ability to inhibit the CDK2 enzyme. 63 was more effective than roscovitine, with an IC_50_ of 0.24 µM. 65 also demonstrated good inhibitory activity, with an IC_50_ of 0.50 µM. Furthermore, molecular docking studies were conducted to determine the binding mode, which was consistent with the *in vitro* CDK2 inhibitory activity results.^48^

Amr S Abu Lila *et al.* reported the design and synthesis of pyridine-3,5-dicarbonitrile derivatives. The synthesis of target 68 was straightforward, as illustrated in Scheme 12. 2-Benzylidenemalononitrile 66 reacted with aniline 67 in the presence of ZnCl_2_ in ethanol under reflux, afforded the desired target 68 (yield: 90%).^81^

**Scheme 12:**
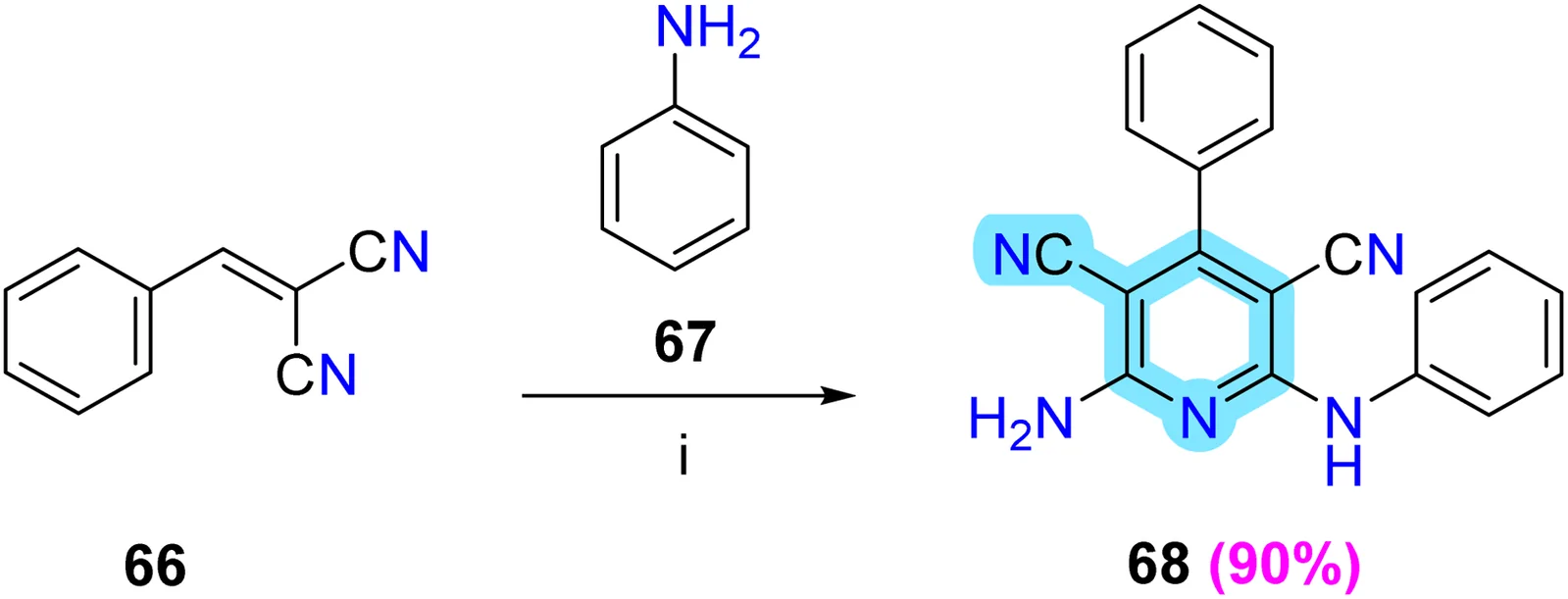
Synthesis of compound 68; reagents and conditions: (i) ZnCl_2_, EtOH, stirring, reflux, 24 h.

Compound 68 showed significant cytotoxic activity against 4 human cancer cell lines: HepG2, MDA-MB-231, PC-3 and HELA using 5-fluorouracil (5-FU), as a standard anticancer compound. 68 demonstrated the most potent IC_50_ values of 0.242 and 0.56 µM against HepG2 and PC-3, respectively. The ability of 68 to interact with CDK2 active sites and to inhibit its activity was also confirmed by docking studies.^81^

As CDK2 and/or CDK9 inhibitors, Basma S. Almansour *et al.*, prepared novel 4-aryl-3-(4-methoxyphenyl)-1-phenyl-1*H*-pyrazolo[3,4-*b*]pyridines. 3-(4-Methoxyphenyl)-3-oxopropane-nitrile 71 was prepared through the bromination of 4-methoxyacetophenone 69 to afford 70 followed by cyanation reaction. Then, the key intermediate 73 was prepared *via* the reaction of 71 with phenyl hydrazine 72 in refluxing ethanol. Moreover, one-step three-component reaction of pyrazol-5-amine 73, 3-oxo-3-phenylpropanenitrile 74 and 4-hydroxybenzaldehyde 75 gives the desired pyrazolo[3,4-*b*]pyridine-5-carbonitrile 79. The latter reaction proceeded *via* the condensation reaction between 3-oxo-3-phenylpropanenitrile 74 and 4-hydroxyaldehyde 75, then the addition of H4 of pyrazole to give intermediate 76. The intramolecular cyclocondensation of intermediate 76 gave the target 79 (yield: 77%) Scheme 13.^82^

**Scheme 13:**
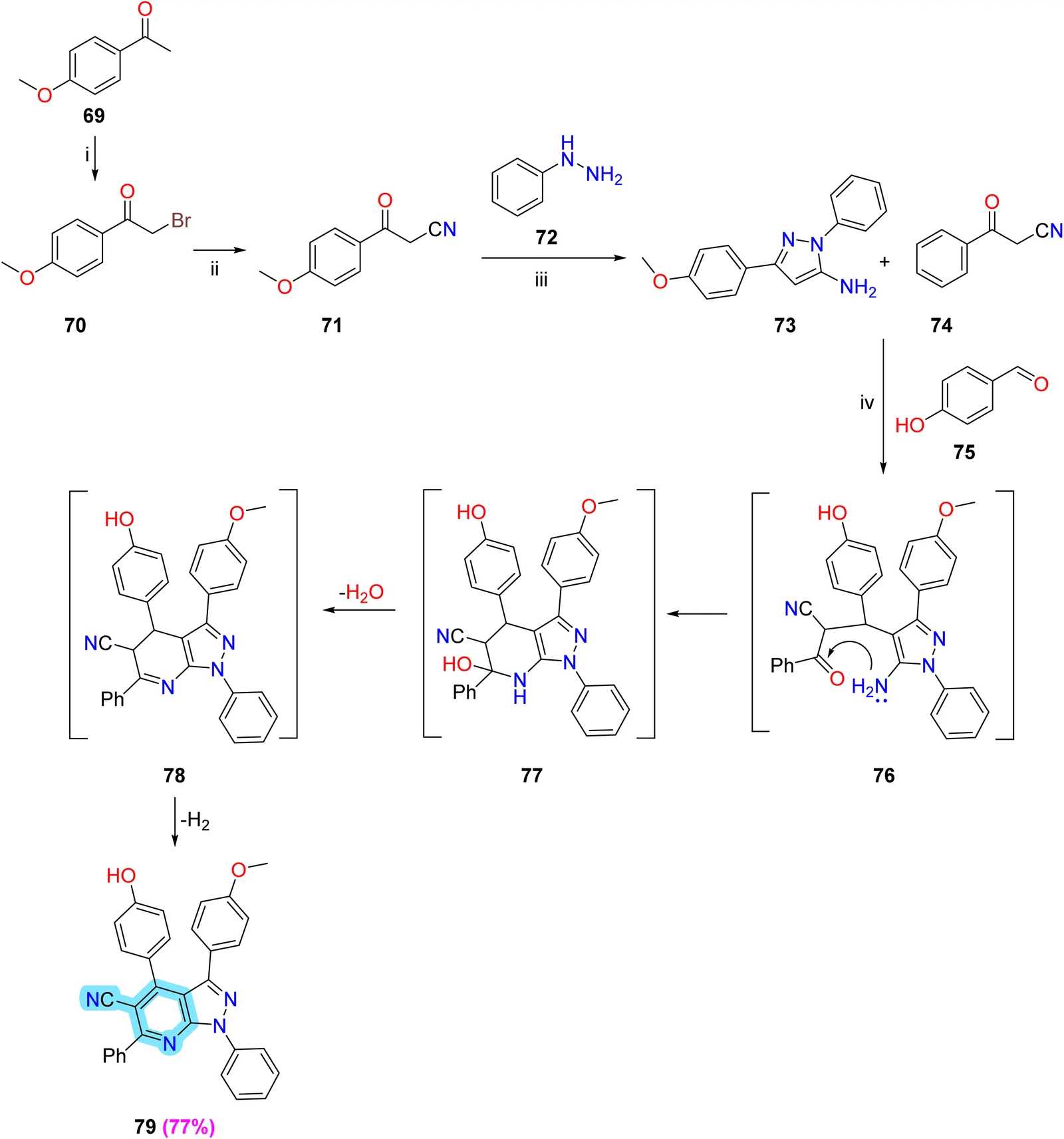
Synthesis of compound 79; reagents and conditions: (i) Br_2_, AcOH, r.t, 1 h; (ii) KCN, EtOH/H_2_O, r.t, 2 h; (iii) EtOH, reflux, 6 h; (iv) EtOH, TEA (cat.), reflux, 6–10 h.

The cytotoxicity of compound 79 against three cell lines; HeLa, MCF-7, and HCT-116, compared with DOX, was *in vitro* evaluated. 79 showed significant anticancer activity with IC_50_ values of 12.65, 4.66, and 1.98 µM, respectively, compared to DOX with IC_50_ = 2.35, 4.57, and 2.11 µM, respectively. In addition, 79 showed good inhibition activity towards CDK2 and CDK9 with IC_50_ = 0.460 ± 0.024 and 0.801 ± 0.041 µM, respectively, when compared with ribociclib (IC_50_ = 0.068 ± 0.004 and 0.050 ± 0.003 µM, respectively). Docking studies for 79 showed good fitting to the CDK2 and CDK9 active sites.^82^

Samir Bondock *et al.*, reported the synthesis of novel *N*-thiazolylpyridylamines targeting CDK2. Synthesis started with triethylamine-catalyzed cyclocondensation reaction of 1-cyanoacetylthiosemicarbazide 80 with 2,4-pentandione 81 in refluxing ethanol, yielding pyridyl thiourea 82. When pyridyl thiourea 82 was subjected to a Hantzsch reaction with α-bromoketone 83 in boiling ethanol and in the presence of TEA, the target thiazolyl pyridyl amine 84 was obtained (yield: 85%), Scheme 14.^83^

**Scheme 14:**
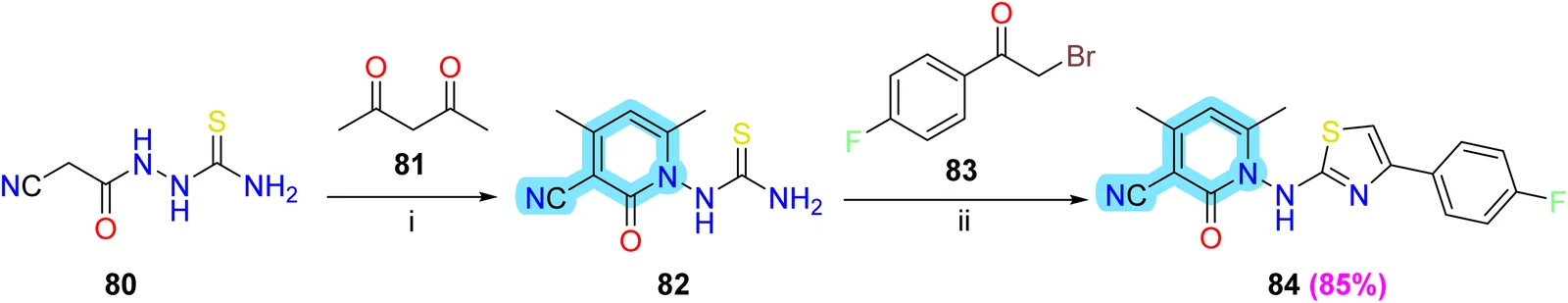
Synthesis of compound 84; reagents and conditions: (i) EtOH, TEA (cat.), reflux, 4 h; (ii) EtOH, TEA (cat.), reflux, 6 h.

Compound 84 demonstrated promising cytotoxic activities against MCF-7, HepG2, and HCT-116, with IC_50_ values of 10.9 ± 0.5, 6.98 ± 0.3, and 6.3 ± 0.2 µM, respectively. Results from molecular docking and dynamic simulations suggest that 84 is a promising cyclin-dependent kinase 2 (CDK2) inhibitor.^83^

Eman Mansour and her colleagues reported the synthesis and anticancer evaluation of new pyridine-3-carbonitrile derivatives. The synthesis of the target compound 91 is illustrated in Scheme 15. The starting 86 was prepared by one-pot multicomponent reaction of benzo[*d*][1,3]dioxole-5-carbaldehyde 85, 1-(naphthalen-2-yl)ethan-1-one 45, and ethyl cyanoacetate 18 in ethanol in the presence of ammonium acetate by stirring at room temperature. Moreover, the reaction of 86 with ethyl bromoacetate 87 by refluxing in dry acetone and K_2_CO_3_ yielded the ethyl acetate ester derivative 88. The latter 88 was allowed to react with hydrazine hydrate in boiling absolute ethanol, which afforded the acetohydrazide derivative 89. Then, refluxing 89 with 4-chlorobenzoic acid 90 in phosphorous oxychloride and ethanol solution yielded 2,5-disubstituted-1,3,4-oxadiazole 91 (yield: 60%).^84^

**Scheme 15:**
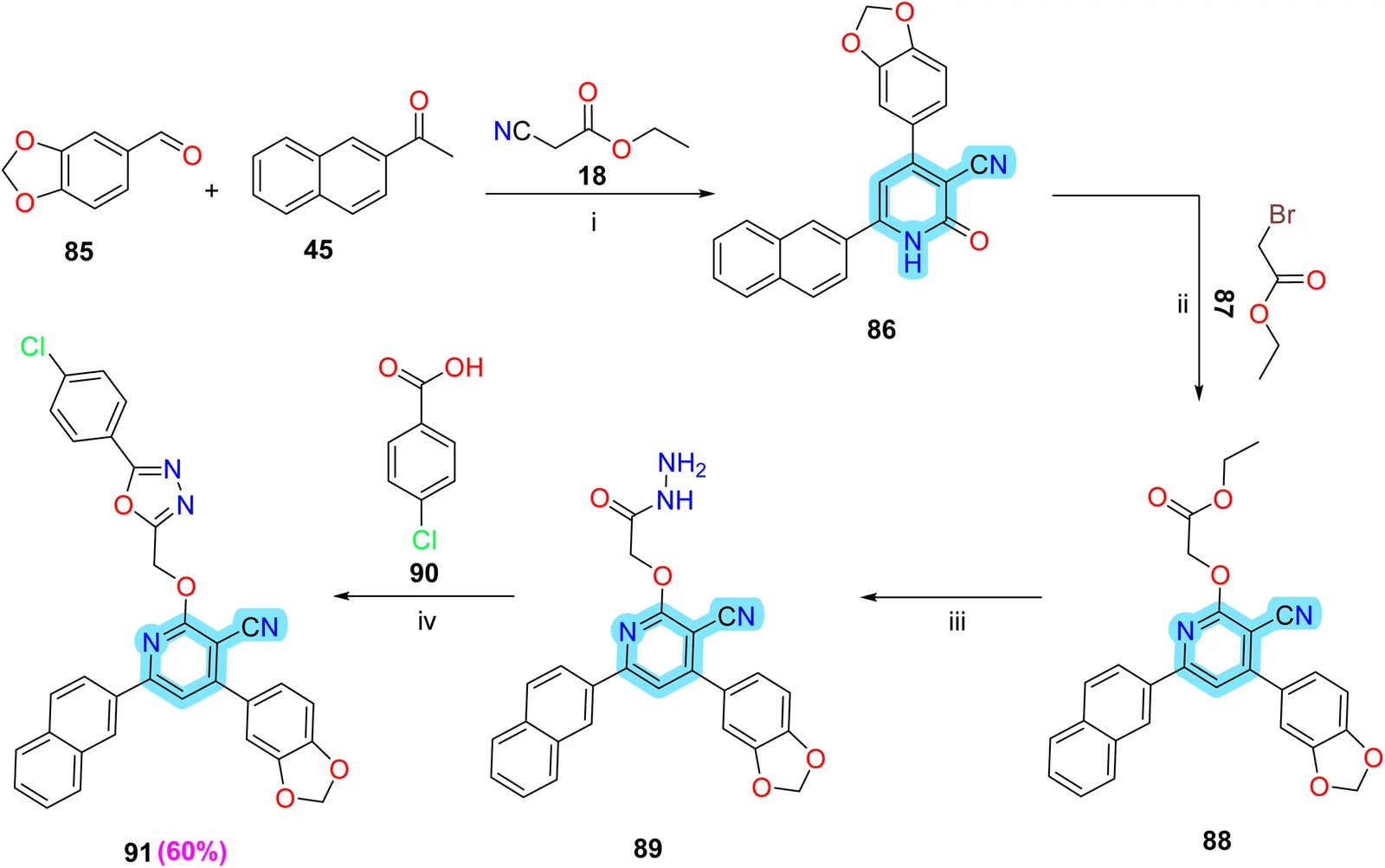
Synthesis of compound 91; reagents and conditions: (i) AcONH_4_, EtOH, stirring, 12 h.; (ii) K_2_CO_3_, DMF, reflux, 20 h; (iii) N_2_H_4_·H_2_O, EtOH, reflux, 6 h; (iv) POCl_3_, EtOH, reflux, 6 h.

The cytotoxicity of compound 91 was investigated against human breast cancer MCF-7 and MDA-MB-231 cell lines. 91 showed the lowest IC_50_ values of 26.95 and 29.08 µg mL^−1^, respectively compared to reference drug 5-FU. Docking studies revealed a stable complex and strong binding affinity of 91 to CDK2.^84^

#### 3.1.4. PIM kinase inhibitors

Shaimaa M. Aboukhatwa and her team reported the design and synthesis of nicotinonitrile-derived apoptotic inducers. Chemical synthesis of the target 94 was done following the procedure outlined in Scheme 16. One-pot condensation reaction of *p*-dimethylamino benzaldehyde 92, 4-methoxyacetophenone 69, ethyl cyanoacetate 18, and ammonium acetate in anhydrous ethanol, yielded the 3-cyanopyridin-2-one derivative 93. Refluxing compound 93 with POCl_3_ gave the 2-chloronicotinonitrile 94 (yield:70%).^27^

**Scheme 16:**
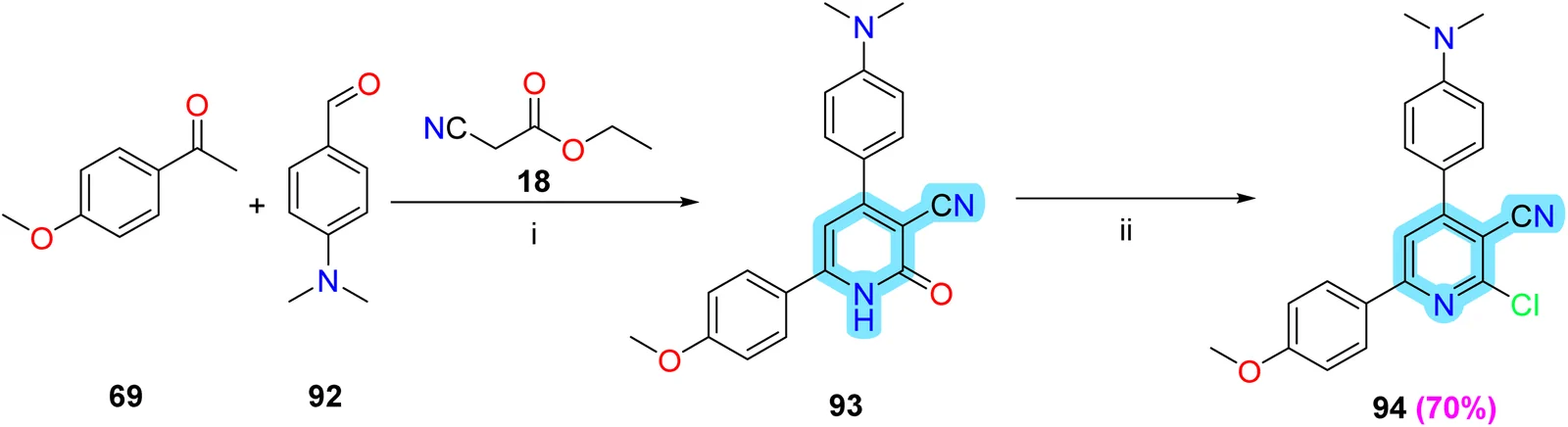
Synthesis of compound 94; reagents and conditions: (i) AcONH_4_, EtOH, reflux, 10 h; (ii) POCl_3_, reflux, 24 h.

Compound 94 demonstrated impressive cytotoxicity, showing IC_50_ values of 0.35 µM for HepG2 and 18.24 µM for MCF-7 cells. Additionally, 94 significantly inhibited PIM-1 kinase with an IC_50_ of 0.28 µM, compared to the reference drug Staurosporine, which had an IC_50_ of 0.46 µM. Docking studies, along with molecular dynamic simulations, revealed a stable complex and strong binding affinity of 94 to PIM-1 kinase.^27^

Basem Mansour *et al.* reported the design and synthesis of cyanopyridinone and cyanopyridine-based cancer cell PIM-1inhibitors. The reaction sequences employed for the synthesis of the targeted compound 99 are illustrated in Scheme 17. The synthesis started with the preparation of intermediate 2-chloroquinoline-3-carbaldehyde 96 by cyclization of *N*-phenylacetamide 95 with Vilsmeier's reagent DMF/POCl_3_.^85^ Then, a one-pot four-component reaction of intermediate 96, 4-bromoacetophenone 97, ethyl cyanoacetate 18, and ammonium acetate in refluxing ethanol was adopted to afford the targeted 98. Finally, deoxychlorination of the cyanopyridinone derivative 98 was achieved by the reaction with POCl_3_ under solvent-free and reflux conditions to afford the corresponding 2-chlorocyanopyridine derivative 99 (yield: 85%).^65^

**Scheme 17:**
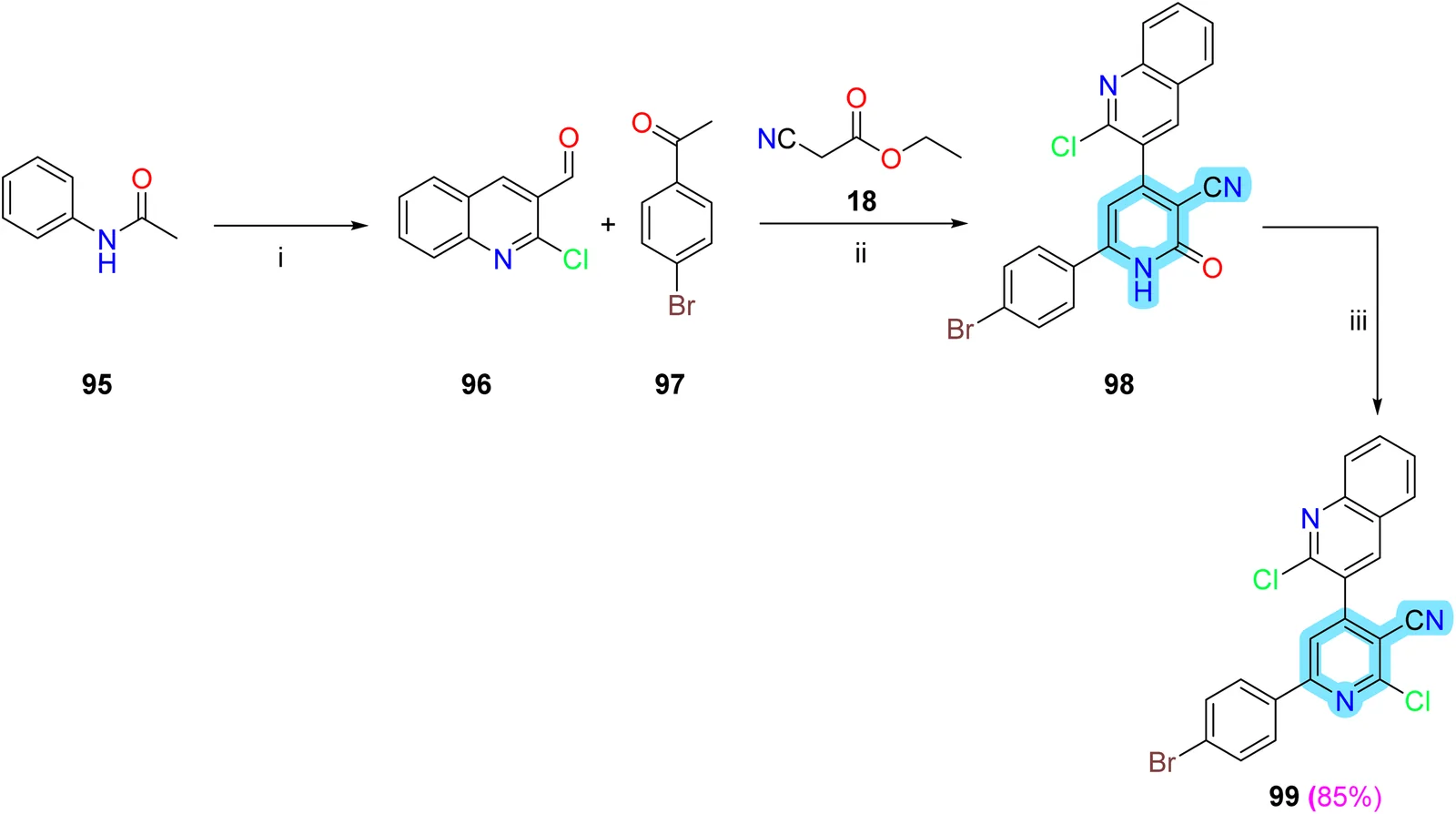
Synthesis of compound 99; reagents and conditions: (i) PC1_5_, DMF, reflux, 4–16 h; (ii) AcONH_4_, EtOH, reflux, 12 h; (iii) POCl_3_, reflux, overnight.

Compound 99 showed promising cytotoxicity against HepG-2, HCT-116, MCF-7, and PC-3 cell lines with IC_50_ values of 6.95, 8.35, 8.50, and 14.08 µM, respectively, compared to 5-FU (IC_50_ = 9.42, 8.01, 7.75, and 10.34 µM, respectively). 99 had the highest PIM-1 kinase inhibitory activity (IC_50_ = 0.46 µM), more effectively than quercetagetin (IC_50_ = 0.56 µM) and being twice as effective as PIM-1 kinase inhibitor A (IC_50_ = 0.93 µM). The ability of 99 to interact with PIM-1 kinase was also confirmed by docking studies.^65^

Mohamed S. Nafie and his colleagues reported the synthesis of novel pyridine conjugates as PIM-1 kinase inhibitors and caspase activators. The synthesis started with one-pot condensation of vanillin 46, 4-chloroacetophenone 100, ethyl cyanoacetate 18 in the presence of ammonium acetate in refluxing acetic acid. Then, the obtained nicotinonitrile 101 was subjected to an alkylation reaction with ethyl bromoacetate 87 in the presence of K_2_CO_3_ to afford the respective *N*-alkylated analogue 102. Finally, hydrazinolysis of ester 102 with hydrazine hydrate in refluxing ethanol afforded the desired product 103 (yield: 90%) Scheme 18.^86^

**Scheme 18:**
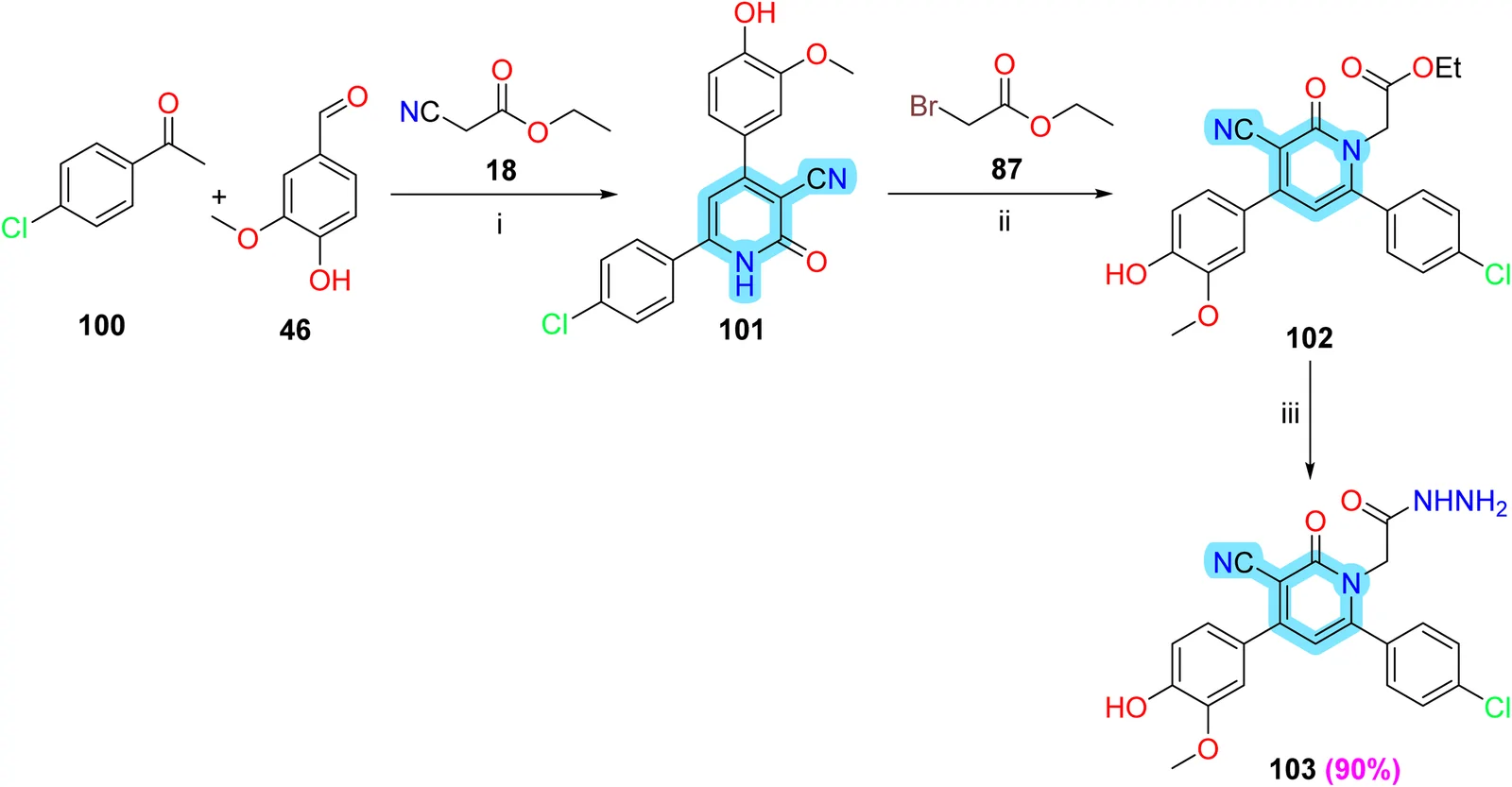
Synthesis of compound 103; reagents and conditions: (i) AcONH_4_, EtOH, reflux, 6 h; (ii) K_2_CO_3_, DMF, reflux, 2 h; (iii) N_2_H_4_·H_2_O, EtOH, reflux, 4 h.

Compound 103 exhibited notable cytotoxicity against the tested cell lines; HepG2 and MCF-7 with IC_50_ values of 0.28 and 0.44 µM, respectively. The inhibitory effects of 103 against PIM-1 kinase were also significant, with an IC_50_ of 8.4 nM, resulting in a 98.8% inhibition, compared to the reference drug staurosporine (IC_50_ = 16.7 nM) and a percent inhibition of 95.6%. The ability of 103 to interact with PIM-1 kinase was also confirmed by docking studies.^86^

Shrouk M. Shaban and her team reported the design and synthesis of novel pyridine-based compounds as potential PIM-1 kinase inhibitors. The synthetic strategy for the desired target 107 is straightforward and is illustrated in Scheme 19. First, 2-[(3-cyano-4,6-dimethylpyridin-2-yl)oxy]-acetohydrazide 104 is converted to azide 105 by the reaction with sodium nitrite and HCl under low temperature (−5 °C). Then, the azide 105 is coupled with morpholine 106 in the presence of Et_3_N to yield the desired product 107 (yield: 66%).^87^

**Scheme 19:**
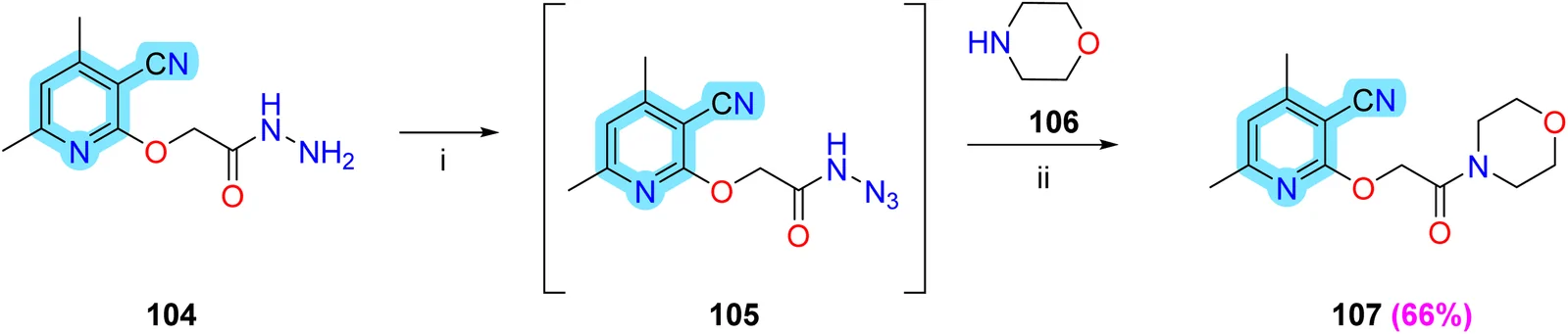
Synthesis of compound 107; reagents and conditions: (i) NaNO_2_/HCl, stirring, −5 °C, 30 min: (ii) Et_3_N, stirring, 0 °C, 20 min.

Compound 107 showed potent cytotoxic activity against MCF-7 and HepG2 cell lines, with IC_50_ values of 0.51 and 5.27 µM, respectively, in contrast to DOX, which had IC_50_ values of 2.14 and 2.48 µM, respectively. The ability of 107 to inhibit PIM-1 kinase was evaluated and showed significant activity with IC_50_ value of 14.3 nM, with percentage inhibiting activity of 97.5% in comparison to staurosporine (IC_50_ = 16.7 nM, 96.8%). Docking studies revealed a stable complex and strong binding affinity of 107 to PIM-1 kinase.^87^

Amel M. Farrag *et al.* developed a new cyanopyridine-based scaffold as PIM-1 inhibitors and apoptotic inducers. Synthesis of the target cyanopyridine 110 was accomplished in Scheme 20. First, nucleophilic substitution reaction of 4-amino-acetophenone 57 and phthalic anhydride 108 yielded the intermediate, 2-(4-acetylphenyl)isoindoline-1,3-dione 109. Then, the acetyl derivative 109 was condensed with furfural 41 and malononitrile 8 in the presence of excess ammonium acetate in acetic acid to give the corresponding cyanopyridone 110 in a one-pot reaction (yield: 75%).^30^

**Scheme 20:**
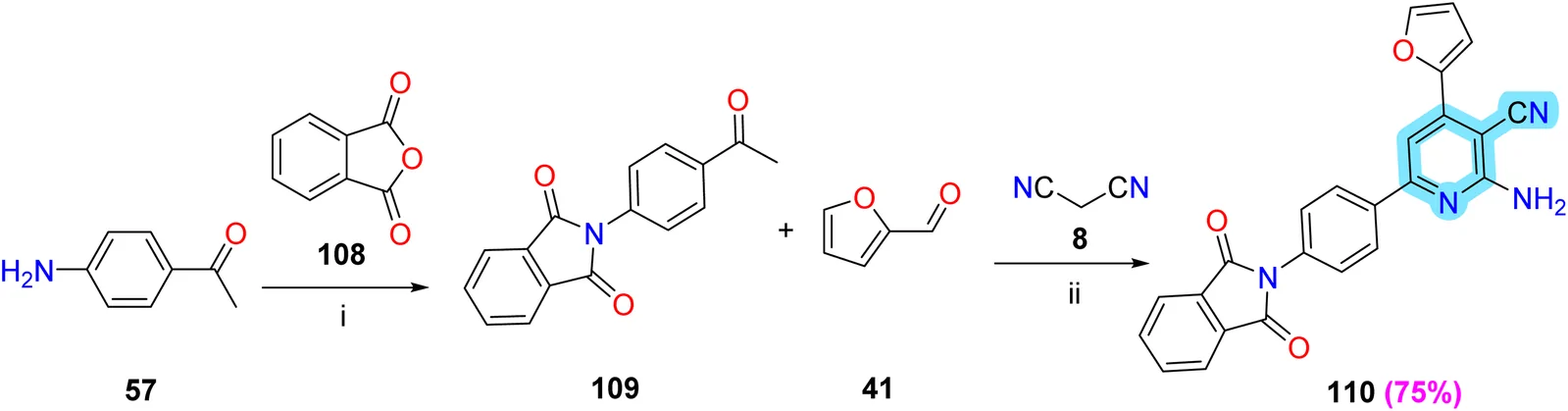
Synthesis of compound 110; reagents and conditions: (i) AcOH, reflux, 4 h; (ii) AcONH_4_, gl. AcOH, reflux, 12 h.

Compound 110 was examined for its antiproliferative activity against three human cancer cell lines PC-3, HepG2, and MCF-7. 110 showed significant anticancer activity against the tested cancer cell lines PC-3, HepG2, and MCF-7, with IC_50_ values of 4.82, 5.19, and 8.17 µM, respectively, compared with 5-FU, with IC_50_ values of 7.53, 8.15, and 7.76 µM, respectively. The inhibitory effect of 110 against PIM-1 kinase was also significant, with IC_50_ of 0.63 µM and a percent inhibition of 76.43%, compared to the reference standard colchicine (IC_50_ = 3.98 µM, 64.15%). Molecular modeling studies were carried out on 110 and showed perfect fitting in the PIM-1 active site.^30^

Mona H. Ibrahim *et al.* reported the development of novel cyanopyridines as PIM-1 kinase inhibitors. The targeted compounds' synthesis is highlighted in Scheme 21. Initially, a mixture of 4-hydroxyacetophenone 111, benzyl chloride 112, and KOH in ethanol was refluxed for 3 h. Additionally, Knoevenagel condensation of 113 with 4-*N*-dimethylamino benzaldehyde 92 in MeOH and in the presence of KOH, produced chalcone 114. Furthermore, chalcone 114 reacted in methanol with malononitrile 8 in the presence of fresh sodium methoxide, yielding pyridine derivative 115 (yield: 96%).^88^

**Scheme 21:**
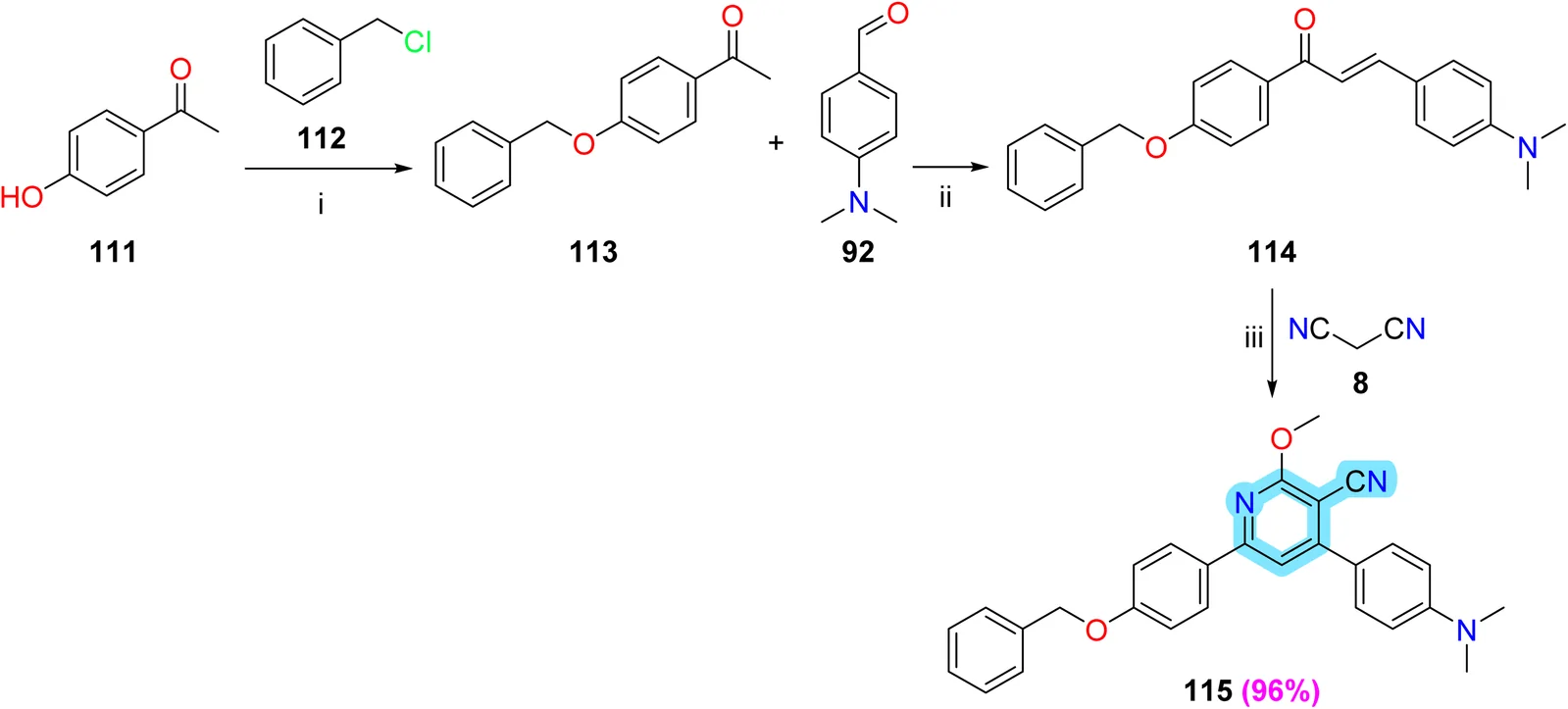
Synthesis of compound 115; reagents and conditions: (i) KOH, EtOH, reflux, 3 h; (ii) 10% KOH, MeOH, stirring, 2 h; (iii) NaOMe, MeOH, reflux, 4 h.

Cyanopyridine 115 was evaluated for its cytotoxicity against two prostatic cancerous cell lines PC-3 and DU-145. 115 showed cytotoxic activity with IC_50_ values of 0.99 and 8.03 µM, against PC-3 and DU-145 cell lines, respectively, compared to the reference drug staurosporine (IC_50_ = 2.08 µM and 13.4 µM, respectively). 115 showed lower cytotoxicity toward normal WI-38 cells. Compound 115 exhibited the most potent inhibitory effect on PIM-1 kinase, having IC_50_ = 0.13 µM compared to staurosporine (IC_50_ = 0.294 µM). Molecular docking revealed the ability of 115 to well accommodate the PIM-1 kinase active site, forming stable complexes.^88^

As PIM-1 inhibitors, Bahgat R. M. Hussein *et al.* reported the design and synthesis of novel 3-cyanopyridine derivatives. Among them, 121 was the most active. The synthesis started with *O*-alkylation of vanillin 46 by stirring it with ethanolic NaOEt to give vanillin sodium salt 116, which was then reacted with 2-chloro-*N*-phenylacetamide 117 in dry DMF at 70 °C. The synthetic *O*-alkyl vanillin 118 then underwent Knoevenagel condensation reaction by reaction with ethyl cyanoacetate 18 in the presence of TEA to afford the arylidene 119 in ethanol. Finally, one-pot multi-component reaction of arylidene 119 with 3-acetylpyridine 120 and ammonium acetate in ethanol afforded the desired 121 (yield: 85%)^89^Scheme 22.

**Scheme 22:**
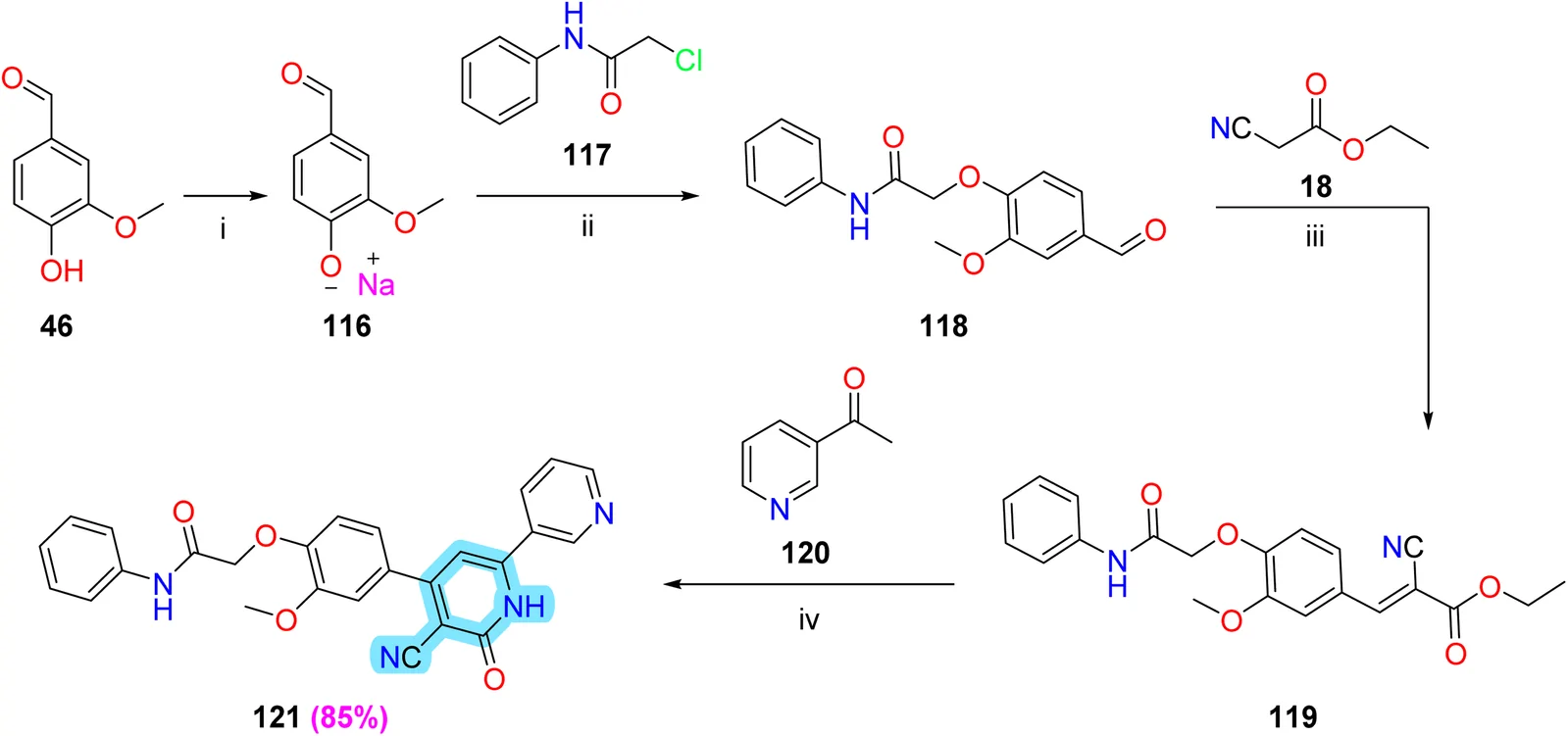
Synthesis of compound 121; reagents and conditions: (i) NaOEt, EtOH, stirring, 0 °C, 1 h; (ii) DMF, 70 °C, 6 h; (iii) Et_3_N, EtOH, reflux., 1 h; (iv) AcONH_4_, EtOH, reflux, 6–8 h.

Compound 121 demonstrated the highest anticancer activity against the MCF-7 cell line with IC_50_ value of 1.89 µM in comparison with DOX (IC_50_ = 11.49 µM). The inhibition activity of 121 against PIM-1 kinase was evaluated and showed an IC_50_ of 0.281 µM compared to the reference drug staurosporine (IC_50_ = 0.223 µM). Molecular docking studies against PIM-1 revealed a good binding mode of the synthesized 121 and showed agreement with the biological results.^89^

Ahmed T. A. Boraei and his researchers developed new substituted 3-cyanopyridine derivatives as potent anti-liver cancer agents. The synthetic route of the target compound 125 is outlined in Scheme 23. Initially, a four-component one-pot synthesis of 4,6-diphenyl-3-cyano-2-pyridone 123 was performed through the reflux of benzaldehyde 33, acetophenone 122, ethyl cyanoacetate 18, and ammonium acetate in *n*-butanol. Additionally, alkylation of 123 with ethyl chloroacetate 2 was done using K_2_CO_3_ in acetone and DMF, which afforded the *O*-alkylated product 124. Finally, the hydrazinolysis of the ester 124 by the reaction with hydrazine hydrate afforded the target hydrazide 125 (yield: 50%).^90^

**Scheme 23:**
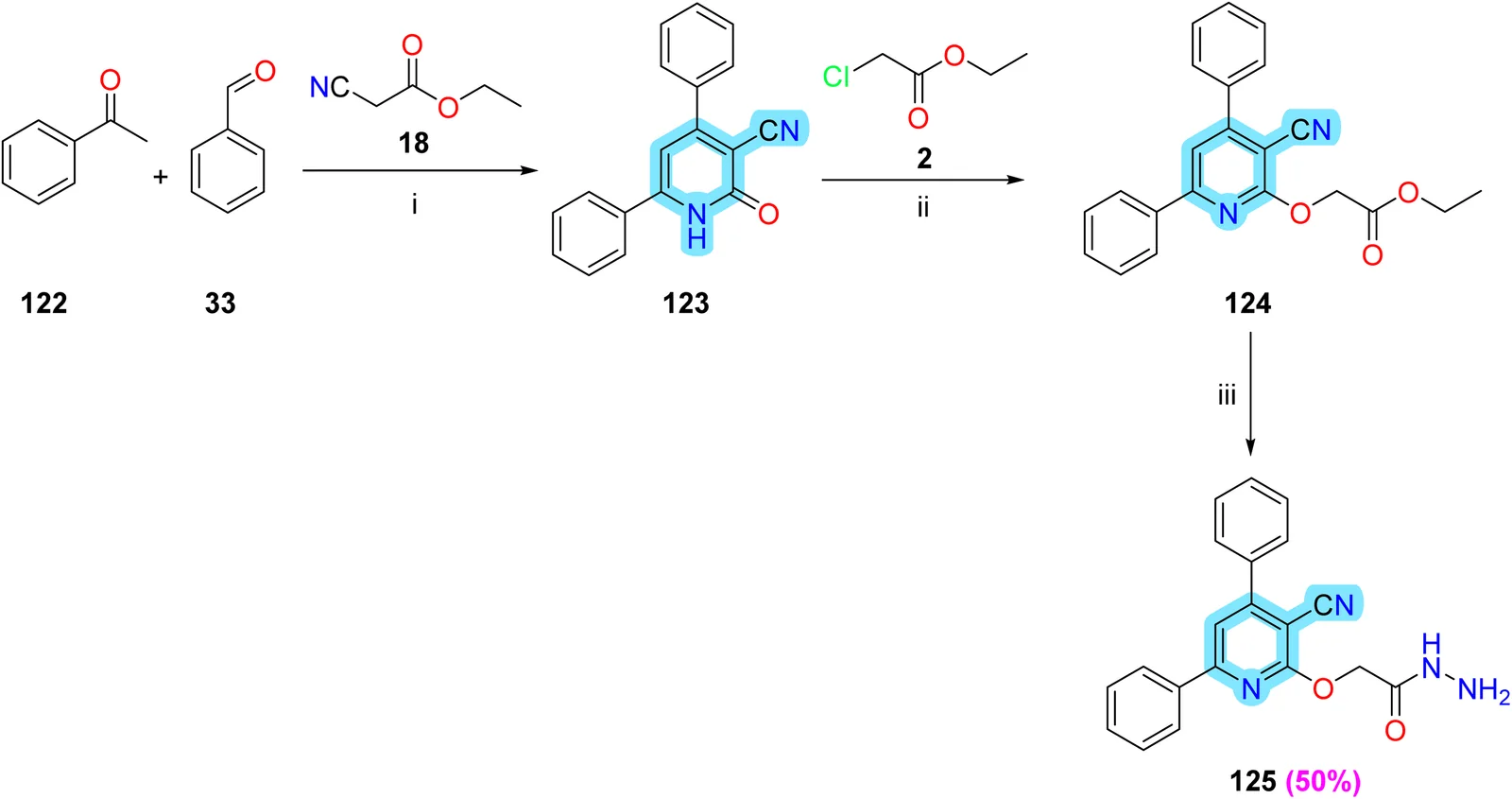
Synthesis of compound 125; reagents and conditions: (i) AcONH_4_, *n*-BuOH, reflux, 1–3 h; (ii) K_2_CO_3_, acetone, DMF, reflux, 5 h; (iii) N_2_H_4_·H_2_O, MeOH, reflux, 3.5 h.

Compound 125 demonstrated cytotoxic action against HepG2 with an IC_50_ value of 7.26 µM compared to 5-FU (IC_50_ = 6.98 µM). 125 did not harm normal liver cells. 125 suppressed PIM-1 kinase gene expression by 0.45-fold. Docking studies revealed that 125 had shown potential binding ability as a PIM-1 inhibitor, which could be the proposed method of action for anti-liver cancer activity.^90^

Magda M. F. Ismail *et al.* reported the synthesis of novel 3-cyanopyridines as a target for anticancer. The synthetic procedure was the same as that adopted for the synthesis of 115 in Scheme 21, except in the final step. Where chalcone 114 reacted with malononitrile/AcONH_4_/EtOH, it yielded the pyridine derivative 126 (yield: 47%) (Scheme 24).^26^

**Scheme 24:**
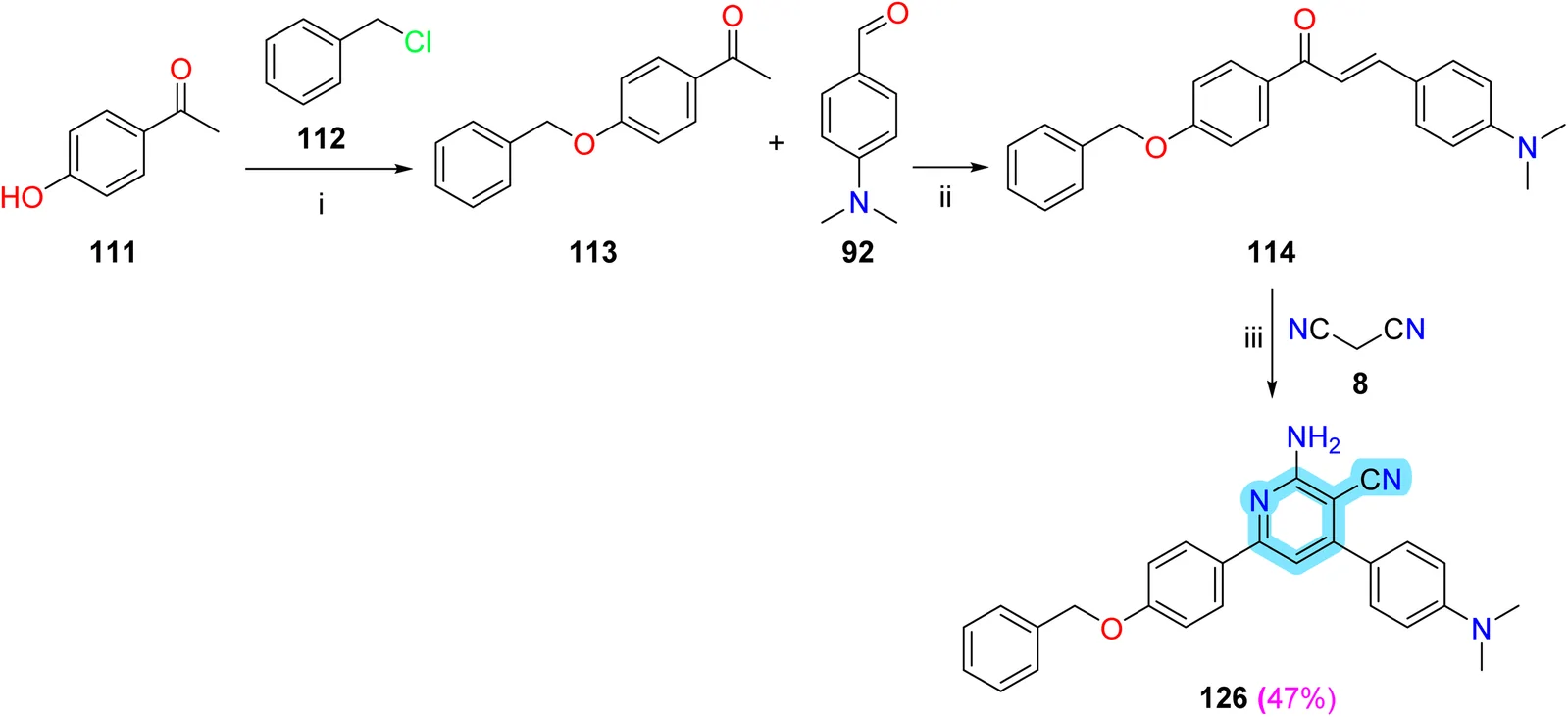
Synthesis of compound 126; reagents and conditions: (i) KOH, EtOH, reflux 3 h; (ii) 10% KOH, MeOH, stirring, 2 h; (iii) AcONH_4_, EtOH, reflux, 9–12 h.

Compound 126 inhibited the development of PC-3, HepG-2, and MCF-7 cancer cell lines with IC_50_ values of 2.04, 3.73, and 8.66 µM, respectively, compared to the reference drug 5-FU (IC_50_ = 7.53, 8.15, and 7.76 µM, respectively). 126 was found to be lower cytotoxicity against the proliferation of normal human cell line (WI-38), with an IC_50_ value of 160.04 µM. Additionally, compound 126, the most effective PIM-1 kinase inhibitor, demonstrated 80.22% suppression of PIM-1 expression at 10 µM with IC_50_ = 0.47 µM, compared to colchicine (64.22%, IC_50_ = 3.98 µM).^26^

Nafie *et al.* 2020 reported the discovery of novel pyrazolo[3,4-*b*]pyridine scaffold-based derivatives as potential PIM-1 kinase inhibitors. Initially, the reaction of compound 127 with hydrazine hydrate afforded 3,6-diamino-4-(*o*-chlorophenyl)-pyrazolo[3,4-*b*]pyridine-5-carbonitrile 128. Treatment of the latter 128 with NaNO_2_ and Conc. HNO_3_ afforded the diazonium salt 129. Finally, the reaction of the diazonium salt 129 with diethylmalonate 130 was carried out in the presence of sodium acetate to give the corresponding cyclic product 131 (yield: 62%)^91^Scheme 25.

**Scheme 25:**
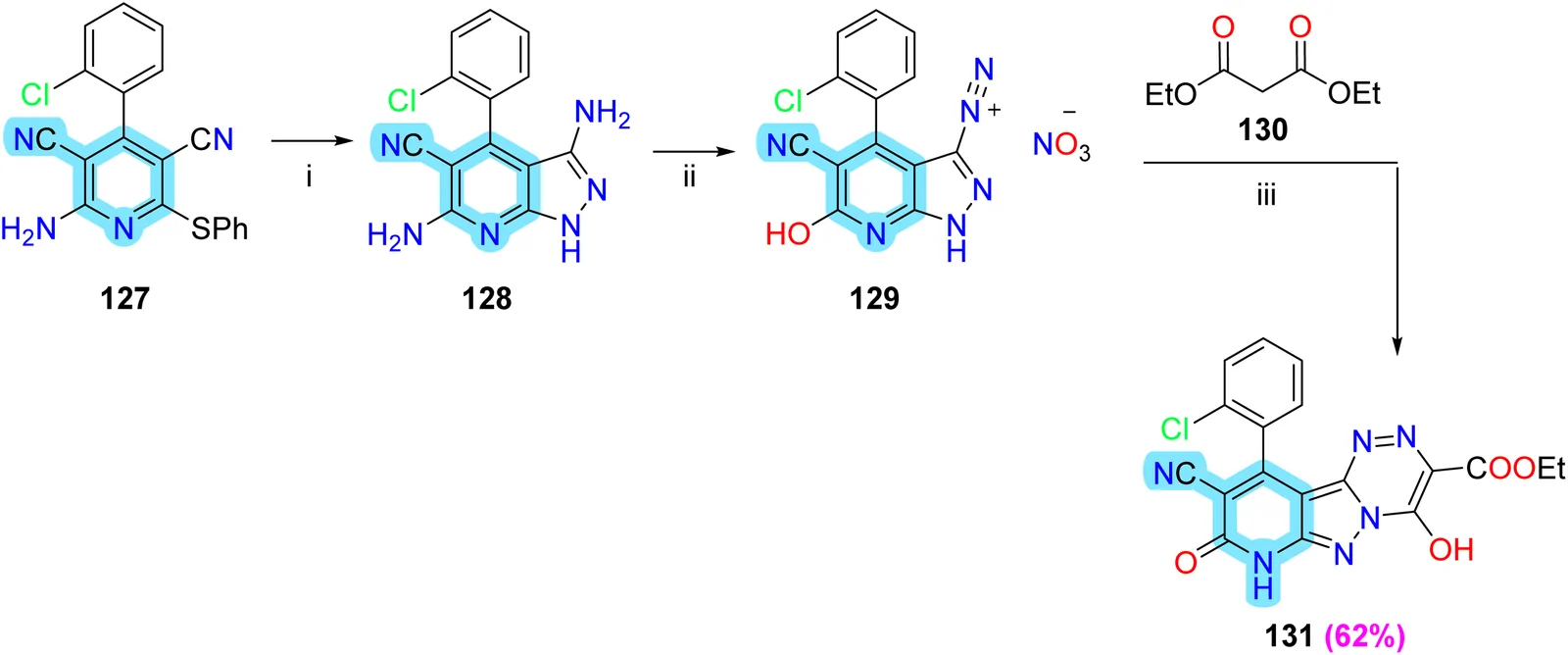
Synthesis of compound 131; reagents and conditions: (i) N_2_H_4_·H_2_O, *n*-BuOH, reflux, 2 h; (ii) 30% NaNO_2_, 63% conc. HNO_3_, stirring, 0–5 °C, 1 h; (iii) CH_3_COONa, EtOH, HCl, stirring, r.t, 3 h.

The antiproliferative activity of compound 131 was evaluated against MCF-7 and MDA-MB-231 cancer cell lines. It showed IC_50_ values of 5.61 µM and 38.7 µM, respectively, compared to 5-FU (IC_50_ values of 5.80 µM and 15.74 µM, respectively). Moreover, 131 showed the highest inhibitory activity with an IC_50_ value of 26 nM, compared to staurosporin (IC_50_ = 17 nM). The docking studies with the tested protein indicated that 131 showed promising binding activity as a PIM-1 kinase inhibitor.^91^

M. M. M. El-Miligy *et al.* reported the design and synthesis of new quinoline–pyridine hybrids. The target compounds 135 and 137 were synthesized according to the steps outlined in Scheme 26. 2,6-Dichloro-3-formylquinoline 132 was reacted with morpholine 106 to give 2-(morpholin-4-yl)quinoline-3-carbaldehyde 133. Then, 133 was subjected to a one-pot reaction with the 3-hydroxyacetophenone 134 and ethyl cyanoacetate 18 in the presence of excess ammonium acetate to give the corresponding 3-cyanopyridone 135 (yield: 40%). On the other hand, for the synthesis of the target 137, hydrolysis of 2-chloro-quinoline-3-carbaldehyde 96 was done to prepare quinolone aldehyde 136. Moreover, the latter quinolone aldehyde 136 reacted with 3-hydroxyacetophenone 134, ethyl cyanoacetate 18, and ammonium acetate to give the corresponding 3-cyanopyridone 137 (yield: 45%).^92^

**Scheme 26:**
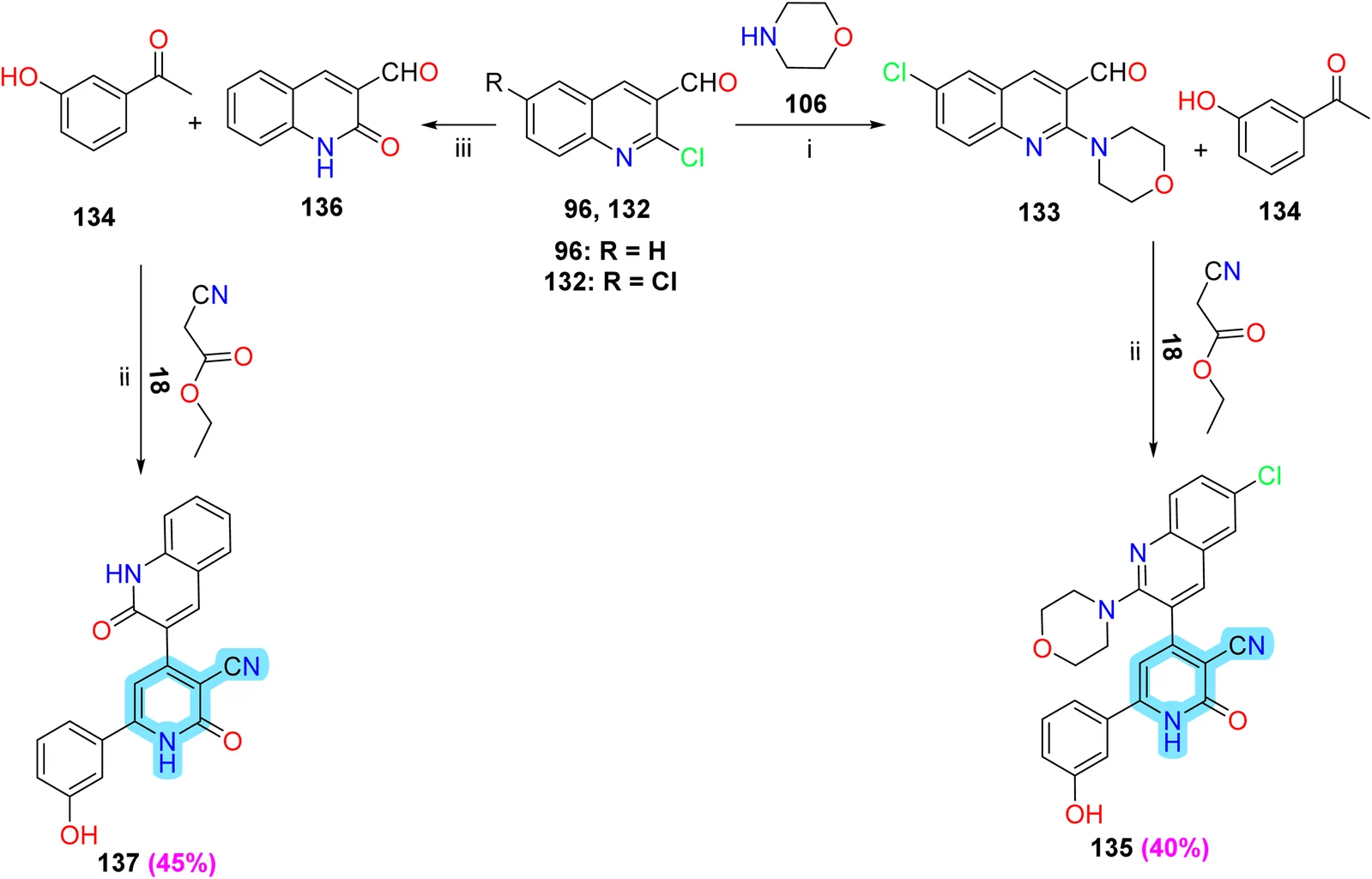
Synthesis of compounds 135 and 137; reagents and conditions: (i) K_2_CO_3_, DMF, reflux, 4 h; (ii) ACONH_4_, EtOH, reflux, 4 h; (iii) AcOH, reflux.

Compounds 135 and 137 demonstrated potent anticancer activity *in vitro* against NFS-60, HepG-2, PC-3, and Caco-2 cell lines while exhibiting low toxicity against the normal human WI-38 cell line. 135 showed anticancer activity with IC_50_ values of 0.00932, 0.00528, 0.0261, and 0.0453 µM, against NFS-60, HepG-2, PC-3, and Caco-2 cell lines respectively, compared to the reference standard. 137 demonstrated an exceptional increase in its anti-tumor activity. It was 21-fold more active against the HepG-2 cell line (IC_50_ = 0.00238 µM) and three-fold more active against the NFS-60 cell line (IC_50_ = 0.009 µM) than the reference (IC_50_ = 0.0504 and 0.0332 µM, respectively). 135 had the highest PIM-1 kinase inhibitory activity (IC_50_ = 1.31 µM) and pim-2 kinase inhibitory activity (IC_50_ = 0.67 µM) compared to quercetin (IC_50_ = 1.17 and 0.61 µM, respectively). 137 displayed inhibitory activity against PIM-1 kinase (IC_50_ = 1.27 µM). Molecular docking investigations revealed that the *in silico* affinity *was* consistent with the observed *in vitro* inhibitory activity against PIM-1/2 kinases.^92^

Marwa E. Abdelaziz and her team developed new pyridine–quinoline hybrids as competitive and non-competitive PIM-1 kinase inhibitors. The target compounds 142a and 142b were synthesized according to the steps outlined in Scheme 27. Initially, 139, using the same procedure as that described for compound 135 in Scheme 26, was subjected to a one-pot reaction with the 3-hydroxyacetophenone 134 and ethyl cyanoacetate 18 in the presence of excess ammonium acetate to give the corresponding 3-cyanopyridone 139. Additionally, synthesis of *O*-substituted pyridine derivatives 141a and 141b were performed *via* treatment of intermediate 140 with alkyl halides, namely ethyl chloride and propyl chloride, in the presence of K_2_CO_3_ to give 141a and 141b. Compounds 141a and 141b were hydrolyzed using sodium bicarbonate in equivalent amounts of methanol and water to prepare the final compounds 142a and 142b (yield: 90% and 80%, respectively).^93^

**Scheme 27:**
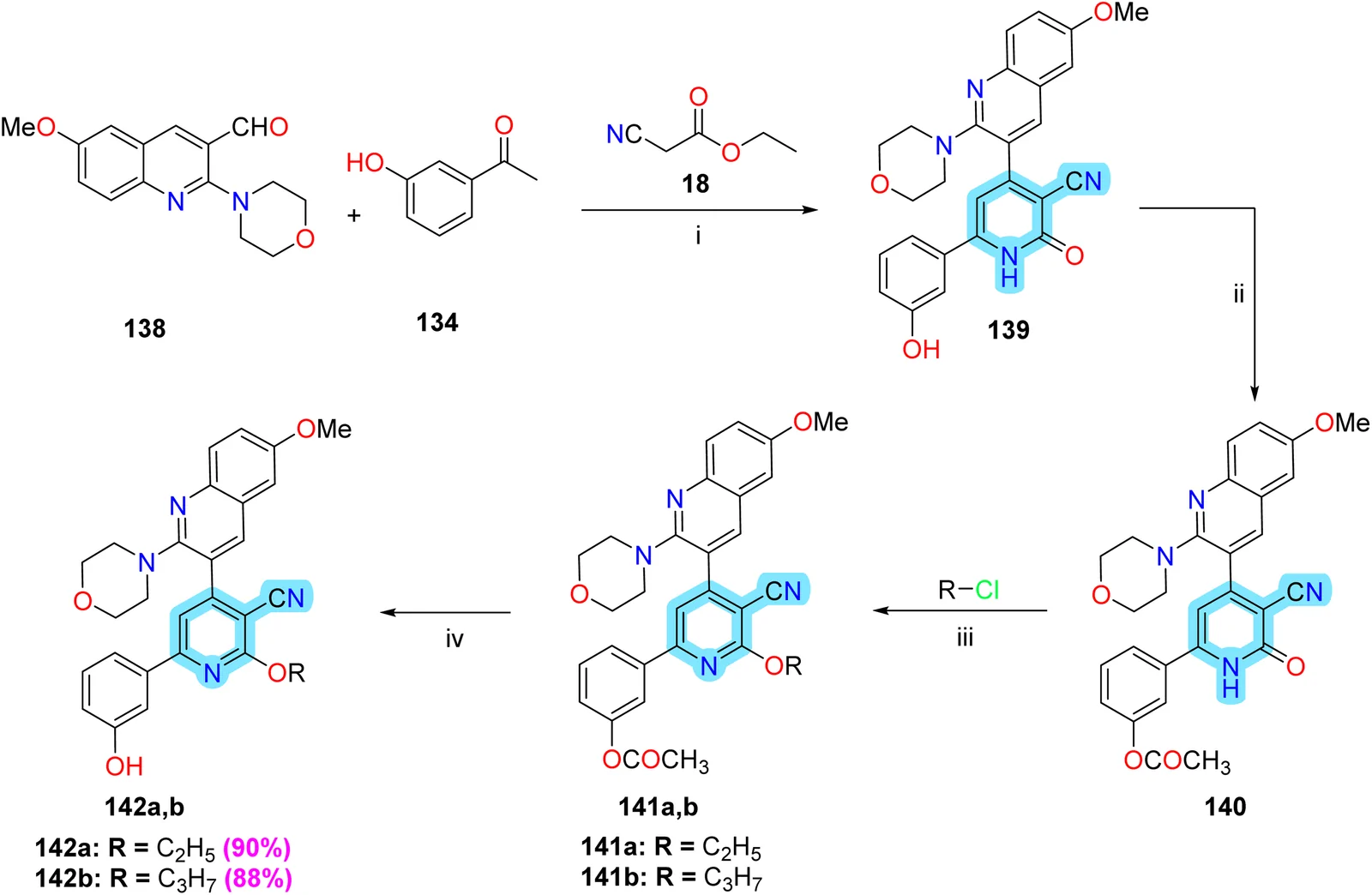
Synthesis of compounds 142a, 142b; reagents and conditions: (i) ACONH_4_, EtOH, reflux, 4 h.; (ii) (CH_3_CO)_2_O, AcOH, reflux, 3–5 h; (iii) K_2_CO_3_, acetone, reflux, 4 h; (iv) NaHCO_3_, MeOH/H_2_O (1 : 1), reflux, 1 h.

Compounds 142a and 142b demonstrated potent *in vitro* anticancer activity against NFS-60, HepG-2, PC-3, and Caco-2 cell lines while exhibiting low toxicity against the normal human WI-38 cell line. 142a outperformed the reference drug Dox in potency against HepG-2, Caco-2, PC-3, and NFS-60 cell lines (IC_50_ = 0.0132, 0.007, 0.005, and 0.006 µM, respectively). 142b significantly improved anticancer activity against HepG-2 cells (IC_50_ = 0.0164 µM) and exhibited potential action against Caco-2, PC-3, and NFS-60 cells with an IC_50_ values of 0.0422, 0.0639, and 0.0646 µM, respectively. The inhibitory effect of compounds 142a and 142b against PIM-1 kinase was also significant with IC_50_ values of 0.110 and 0.095 µM, respectively, compared to the reference standard Quercetin (IC_50_ = 1.17 µM). Docking studies revealed a stable complex and strong binding affinity of compounds 142a and 142b to PIM-1 kinase.^93^

### 3.2. Dual-target compounds

Samy Mohamady *et al.* reported the design and synthesis of a nicotinonitrile scaffold as a promising dual inhibitor of c-Met and PIM-1. Chemical preparation of the target compound 145 was achieved by following the steps described in Scheme 28. Briefly, acetyl phenylpyrazole 143 was prepared by a three-component one-pot reaction using a mixture of phenylhydrazine 72, *N*,*N*-dimethylformamide-dimethyl acetal (DMF-DMA) 10, and 2,4-pentanedione 81. Finally, the targeted nicotinonitrile 145 was obtained by the one-pot condensation reaction of acetyl phenylpyrazole 143, 4-methylbenzaldehyde 144, and ethyl cyanoacetate 18 using anhydrous ammonium acetate, in absolute ethanol (yield: 82%).^25^

**Scheme 28:**
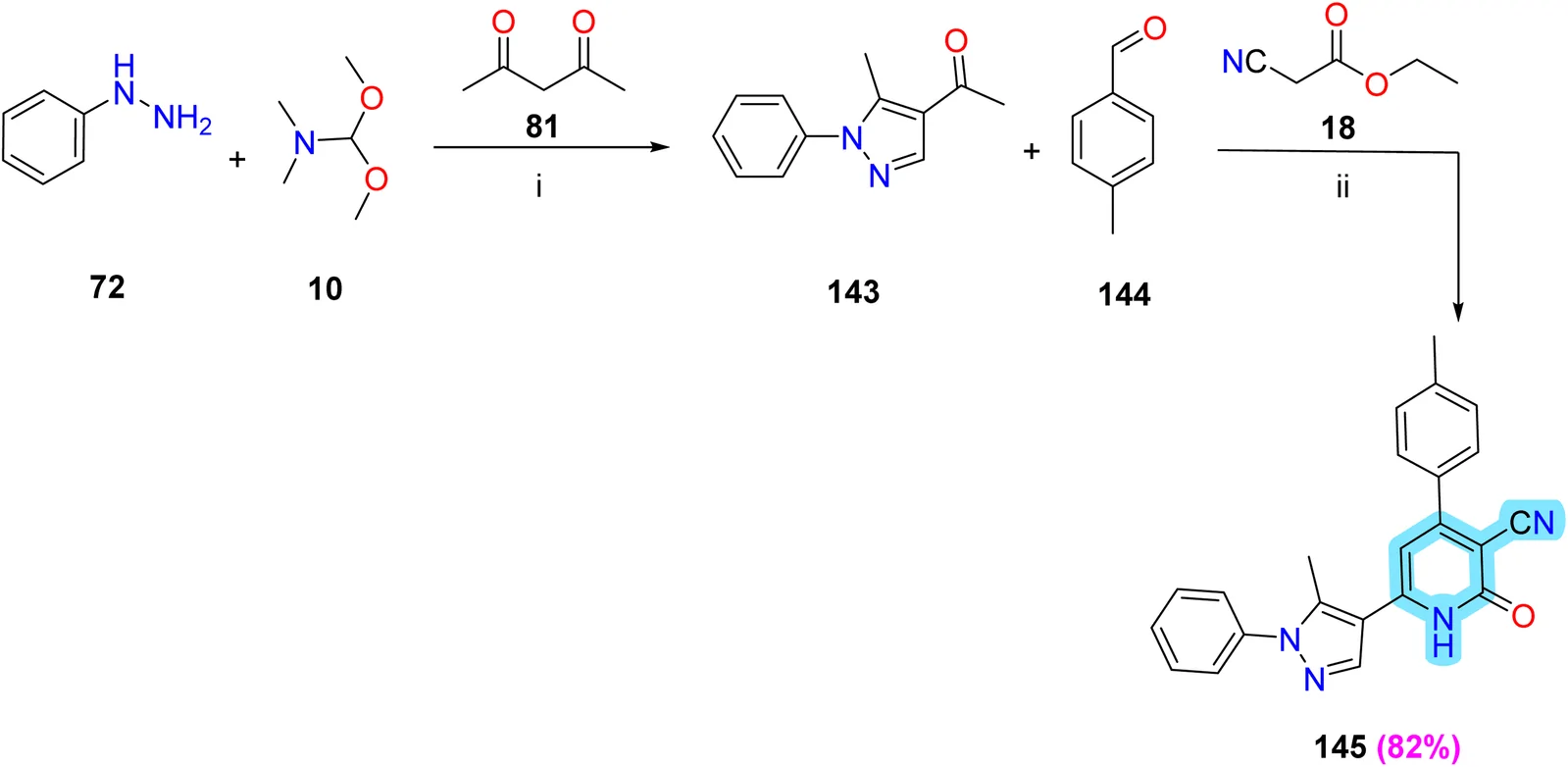
Synthesis of compound 145; reagents and conditions: (i) neat, reflux, 24 h; (ii) AcONH_4_, EtOH, reflux, 24 h.

Compound 145 demonstrated a significant anticancer activity against PC-3, HCT-116, and MDA-MB-231 cancer cell lines, with IC_50_ values of 5.24, 2.79, and 3.71 µM, respectively, compared with tivantinib, the reference drug. Further investigations have confirmed that 145 effectively induces apoptosis through a dual inhibitory pathway by decreasing the production of c-Met and PIM-1 kinase proteins with an IC_50_ of 151 nM compared to tivantinib IC_50_ = 146 nM for c-Met and IC_50_ of 110 nM compared to staurosporine IC_50_ = 276 nM for PIM-1 kinase. Results from molecular docking and dynamic simulations suggest that 145 is a promising candidate for inhibiting both c-Met and PIM-1 kinases.^25^

Samia S. Hawas *et al.* developed new 2-alkoxy-3-cyanopyridine derivatives as inhibitors of EGFR, HER2, and DHFR. The desired 2-alkoxy-3-cyano-4-aryl-6-(*p*-tolyl)pyridine derivatives 148a and 148b were synthesized in two-step reactions (Scheme 29). First, 1-(*p*-tolyl)ethenone 37 was reacted with different substituted aromatic aldehydes, namely, 4-bromobenzaldehyde 146a and 4-nitrobenzaldehyde 146b, in ethanolic NaOH solution *via* Claisen–Schmidt condensation to afford chalcones 147a and 147b. The isolated α,β-unsaturated ketone intermediates 147a and 147b were cyclized with malononitrile 8 in sodium propoxide^94^ to afford the target cyanopyridines, 148a and 148b (yield: 67% and 69%, respectively).^61^

**Scheme 29:**
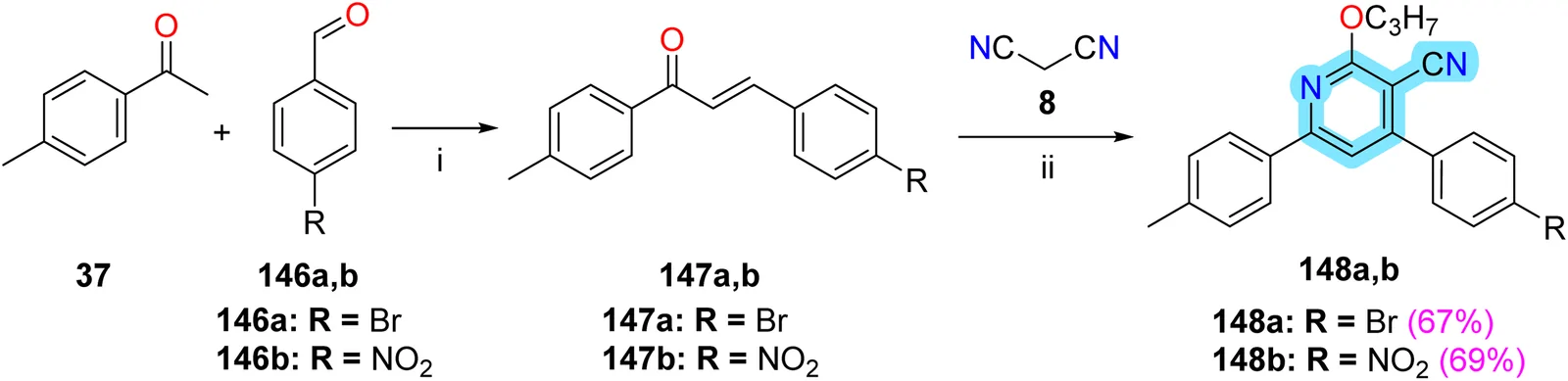
Synthesis of compounds 148a, 148b; reagents and conditions: (i) 3% NaOH, EtOH, r.t., 12 h; (ii) NaOC_3_H_7_, EtOH, r.t., 3 h.

Compounds 148a and 148b were tested against cancer cell lines HepG2, HeLa, HEp-2, HCT-116, PC-3, and MCF-7 for their cytotoxicity. The molecules 148a and 148b had the highest potency against the MCF-7 cell line, with IC_50_ values of 6.16 and 3.84 µM, respectively, compared to sorafenib and DOX as reference drugs. In addition, they were the most efficient dual EGFR/HER2 kinase inhibitors, with IC_50_ values of 0.248 (EGFR), 0.156 (HER2), and 0.138 (EGFR), 0.092 (HER2) µM, respectively. Compounds 148a and 148b had the strongest DHFR inhibitory activity, comparable to methotrexate (MTX), with IC_50_ values of 0.138 and 0.193 µM, respectively. Modeling studies indicated that 148a and 148b showed promising anticancer activity.^61^

Mohamed M. Saleh and his team reported the design and synthesis of 6-indolylpyridone-3-carbonitrile derivatives as “all for one” competitive reversible inhibitors of the MAPK signaling pathway. The target compound 151 was synthesized *via* a four-component one-pot reaction of an ethanolic solution containing equivalent amounts of 3-acetylindole 150, 2,4-dichloro benzaldehyde 149, ethyl cyano acetate 18, and ammonium acetate in excess, as seen in Scheme 30, (yield: 55%).^95^

**Scheme 30:**
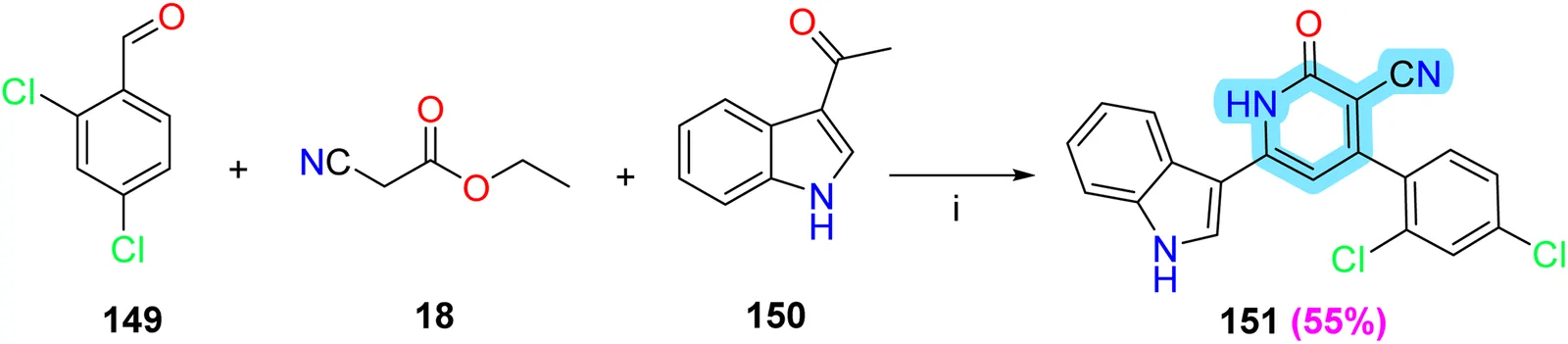
Synthesis of compound 151; reagents and conditions: (i) ACONH_4_, EtOH, reflux, 12 h.

The antiproliferative activity of 151 was evaluated *in vitro* against four human cancer cell lines: SR, HOP-92, RXF 393, and HCT-116. 151 exhibited GI_50_ values of 2.77, 0.75, 3.12, and 2.53 µM against SR, HOP-92, RXF 393, and HCT-116 cell lines, respectively, comparable to staurosporine with GI_50_ = 6.29, 9.99, 0.95, and 6.31 µM. Furthermore, 151 was chosen for extensive mechanistic research, which demonstrated that it blocks many keystones of the MAPK pathway with nanomolar potency (EGFR^WT^ IC_50_ = 281 nM, c-MET IC_50_ = 205 nM, B-RAF^WT^ IC_50_ = 112 nM, and CDK4/6 IC_50_ = 95 and 184 nM). Remarkably, 151 demonstrated significant activity against mutated EGFR and B-RAF, being 4 and 1.3 times more selective to the mutant enzymes than the wild-type versions (EGFR^T790M^ IC_50_ = 69 nM and B-RAF^V600E^ IC_50_ = 83 nM).^95^

Tarfah Al-Warhi *et al.* investigated novel cyanopyridines as anticancer agents with dual VEGFR-2/HER-2 inhibitory actions. The synthetic route for the target molecule 154 is shown in Scheme 31. Through a one-pot reaction of cyanoacetanilide 153, prepared by the reaction 3-trifluoroaniline 152 with ethyl cyanoacetate 18, with 2,4-dichlorobenzaldehyde 149 and malononitrile 8 in EtOH using a catalytic amount of piperidine, yielded the target 154 (yield: 80%).^43^

**Scheme 31:**
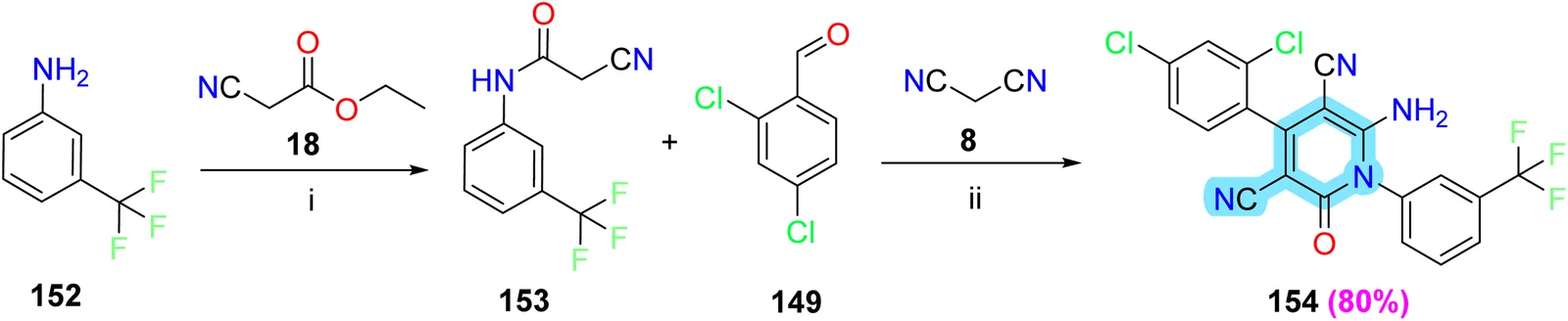
Synthesis of compound 154; reagents and conditions: (i) heating, 180–190 °C, 3 h; (ii) EtOH, piperidine, reflux, 6 h.

Compound 154 was more effective towards the cancer cell lines than the standard treatment taxol. The best antiproliferative activity was demonstrated by cyanopyridone 154 against the MCF-7 and HepG2 cell lines (IC_50_ = 1.39 and 10.70 µM, respectively), compared to standard taxol (IC_50_ = 8.48 and 14.6 µM, respectively). *In vitro* inhibitory activities against VEGFR-2 and HER-2 enzymes were examined for 154. Compound 154 showed the best inhibitory effect (IC_50_ = 0.124 µM) on the VEGFR-2 enzyme in comparison to the standard Lapatinib (IC_50_ = 0.182 µM) and displayed more potent inhibitory effects on HER-2 kinase (IC_50_ = 0.077 µM) than Lapatinib (IC_50_ = 0.131 µM). The results of the docking analysis showed excellent docking scores and interesting binding modes and interactions of 154 with both VEGFR-2 and HER-2 targets, in agreement with their *in vitro* kinase inhibitory activities.^43^

Reham R. Raslan *et al.* reported the synthesis and biological evaluation of 2-oxo-pyridine and 1*H*-spiro-pyridine derivatives as EGFR^Wt^ and VEGFR-2 inhibitors. Initially, ethyl 4-(2-cyanoacetamido)benzoate 156 was prepared by reacting ethyl 4-aminobenzoate 155 with ethyl cyanoacetate 18.^96^ Then, *via* the Michael addition reaction between cyanoacetamide derivative 156 and 2-(2-oxoindolin-3-ylidene)malononitrile 157 in an ethanolic solution catalyzed with piperidine, afforded 158 (yield: 80%)^97^Scheme 32.

**Scheme 32:**
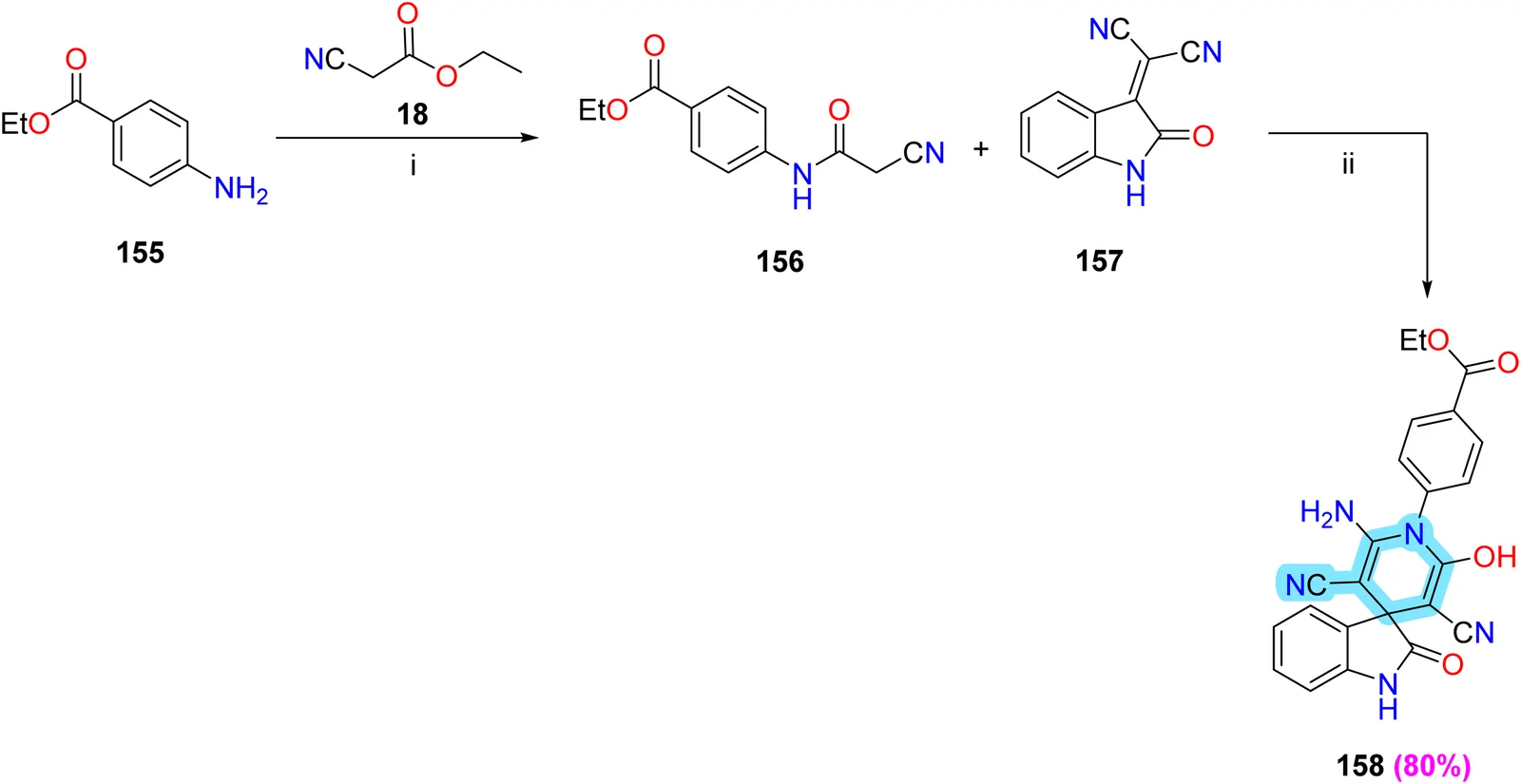
Synthesis of compound 158; reagents and conditions: (i) DMF, (ii) EtOH, piperidine, reflux, 4–6 h.

The antiproliferative activity of compound 158 was *in vitro* evaluated against two human cancer cell lines: HepG-2 and Caco-2. 158 showed the lowest cytotoxicity value against Caco-2 cells (IC_50_ = 7.83 µM) compared with DOX (IC_50_ = 12.49 µM), and on the HepG-2 cell line (IC_50_ = 8.90 µM). Moreover, 158 could inhibit EGFR and VEGFR-2 with sub-micromolar IC_50_ values of 0.124 and 0.221 µM, respectively, compared with erlotinib (IC_50_ = 0.033 µM) and sorafenib (IC_50_ = 0.043 µM). 158 demonstrated good drug-likeness and toxicity profiles. Furthermore, molecular docking studies were conducted to determine the binding mode, which was consistent with the *in vitro* results.^97^

Lamya H. Al-Wahaibi *et al.*, reported the synthesis and biological evaluation of novel 3-cyanopyridone/pyrazoline hybrids as potential EGFR/BRAFV600E inhibitors. The synthetic procedures for the target hybrid 166 are shown in Scheme 33. Initially, 160 was synthesized through a base-catalyzed Claisen–Schmidt condensation of 4-aminoacetophenone 57 with 3,4-dimethoxybenzaldehyde 159. Additionally, Chalcone 160 was reacted with bromoacetyl bromide 161 in K_2_CO_3_ solution to produce the acetylated chalcone 162. On the other hand, the key intermediate, 3-cyano-4,6-bis(phenyl)-pyridone, 164 was efficiently synthesized using a one-pot four-component reaction of 3,4-dimethoxybenzaldehyde 159, 3,4-dimethoxy acetophenone 163, ethyl cyanoacetate 18 and ammonium acetate. Subsequently, the synthesis of *N*-alkylated hybrid 165 was achieved by alkylating 3-cyanopyridines 164 with acetylated chalcones 162. Finally, the target 3-cyanopyridone–pyrazoline hybrid 166 was obtained by refluxing the *N*-alkylated cyanopyridone 165 with hydrazine monohydrate in absolute ethanol for 12 h.^98^

**Scheme 33:**
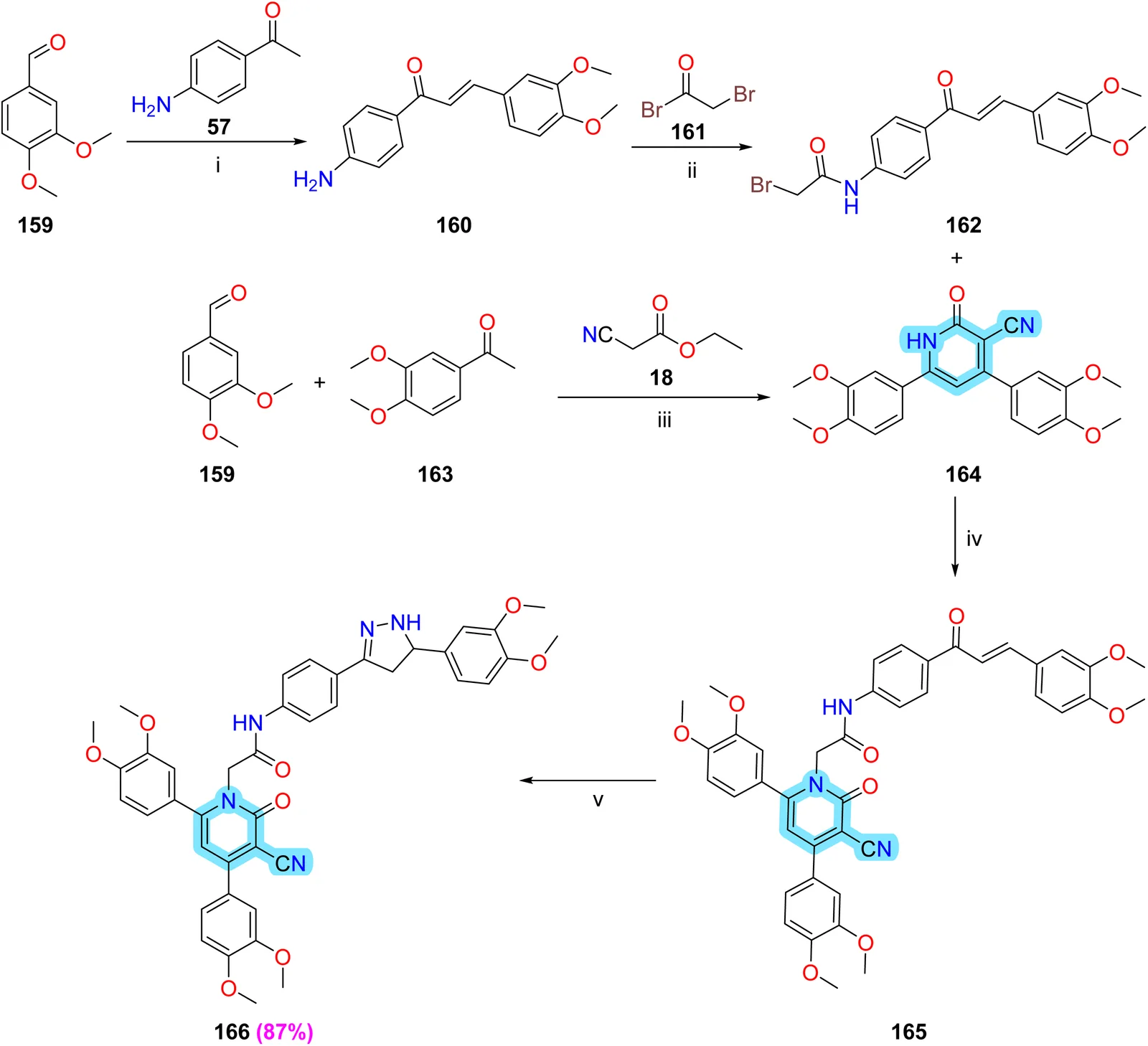
Synthesis of compounds 166; reagents and conditions: (i) 60% NaOH, EtOH, stirring, r.t. 4 h.; (ii) K_2_CO_3_, CH_2_Cl_2_, 0 °C, 2 h; (iii) AcONH_4_, fusion, 15 min; (iv) (a) EtONa, EtOH, r.t. 30 min., (b) DMSO, 80 °C, 4 h; (v) N_2_H_4_·H_2_O, EtOH, reflux, 12 h.

Compound 166 was tested against four human cancer cell lines: HT-29, Panc-1, A-549, and MCF-7 cell lines. It revealed potent antiproliferative activity with IC_50_ values of 24 nM for each HT-29 and Panc-1 cell lines and demonstrated antiproliferative activity against A-549 and MCF-7 with IC_50_ values of 23 and 28 nM, respectively, compared to control erlotinib. 166 had significant EGFR inhibitory effect, with IC_50_ value of 68 nM compared to erlotinib (IC_50_ = 80 nM). Additionally, 166 displayed promising BRAFV600E-suppressive activity, with an IC_50_ of 65 nM, compared to erlotinib's IC_50_ of 60 nM. The docking studies indicated that 166 exhibited substantial antiproliferative action as a dual EGFR/BRAFV600E inhibitor.^98^

Lamya H. Al-Wahaibi *et al.* developed quinazolin-4-one hybridized with 3-cyanopyridin-2-one as dual inhibitors of EGFR and BRAFV600E. The reaction sequences shown in Scheme 34 were used to prepare the desired target 172. Initially, anthranilic acid 167 was reacted with allyl isothiocyanate 168 in refluxing ethanol in the presence of TEA to form 2-mercapto-3-allyl-3*H*-quinazolin-4-ones 169. Subsequently, *N*-(4-acetylphenyl)-2-bromoacetamide 170 was synthesized by treating *p*-aminoacetophenone 57 with bromoacetyl bromide 161 in water and methylene chloride bilayer solvent in the presence of K_2_CO_3_. Consequently, refluxing compounds 169 with 170 in acetonitrile in the presence of TEA afforded the intermediate 171. Finally, one-pot, four-component reaction of equal amounts of acylated quinazolin-4(3*H*)-one 171, ethyl cyanoacetate 18, 4-methoxy benzaldehyde 69, and an excess of ammonium acetate in absolute ethanol yielded the final conjugate 172 (yield: 80%).^99^

**Scheme 34:**
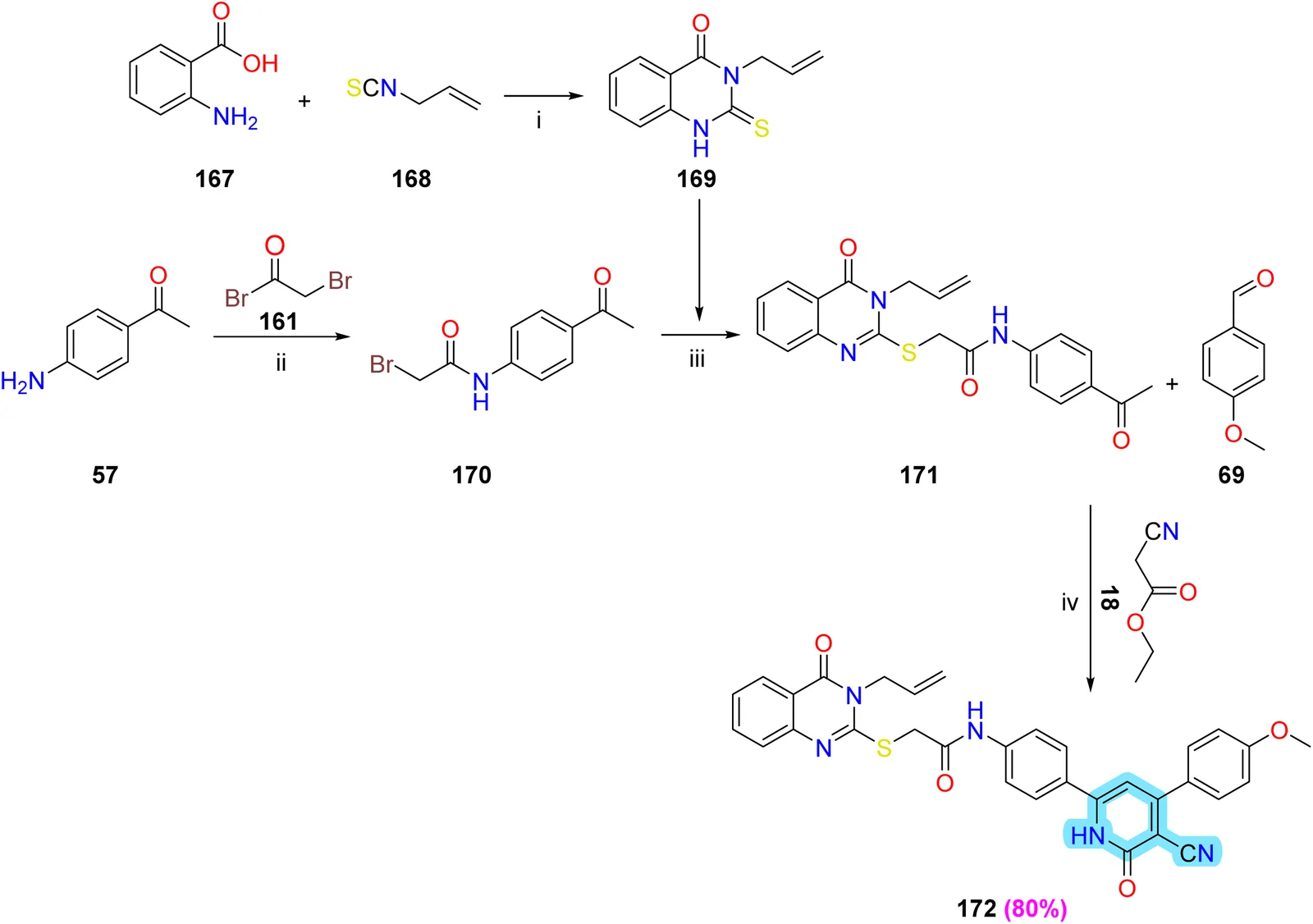
Synthesis of compounds 172; reagents and conditions: (i) TEA/EtOH, reflux, 1.5 h.; (ii) K_2_CO_3_, CH_2_Cl_2_, 0 °C, 2 h; (iii) CH_3_CN, TEA, reflux, 8 h; (iv) AcONH_4_, fusion, 120–130 °C, 1.5 h.

The antiproliferative activity of compound 172 was *in vitro* evaluated against four human cancer cell lines, A-549, MCF-7, Panc-1, and HT-29 with IC_50_ values of 1.1, 1.0, 1.30, 1.30 µM, respectively, compared to DOX (IC_50_ = 1.2, 0.90, 1.40, 1.0 µM, respectively). The most effective antiproliferative 172 was evaluated for inhibition of EGFR as a possible target for its antiproliferative action. 172 had significant EGFR inhibitory effect, with IC_50_ value of 110 nM compared to the erlotinib (IC_50_ value of 80 nM). Additionally, 172 displayed a promising BRAFV600E suppressive activity with IC_50_ values of 140 nM, compared to erlotinib (IC_50_ values of 60 nM). The docking studies indicated that 172 exhibited substantial antiproliferative action as a dual EGFR/BRAFV600E inhibitor.^99^

### 3.3. Cell-based assay-driven compounds

Mohamed R. Elmorsy *et al.* reported the synthesis and biological evaluation of new triphenylamine-linked pyridine analogues as anticancer agents. The synthetic pathway of the target 2-pyridone derivatives 179 is shown in Scheme 35. Firstly, the synthesis of 4-diphenylamino benzaldehyde 174 was done by formylation of triphenylamine 173 according to the modified conditions of Vilsmeier reaction.^100^ Additionally, Knoevenagel condensation of 4-diphenylamino benzaldehyde 174 with the cyanoacetanilide derivative 175 in dry ethanol and piperidine produced the *N*-4-methylphenyl-2-cyano-3-[4-(diphenylamino)phenyl] acrylamide 176. Finally, the targeted 2-pyridone analogue 179 was prepared by the reaction of cyanoacrylamide 176 with malononitrile 8 in ethanol and drops of piperidine (yield: 49%).^101^

**Scheme 35:**
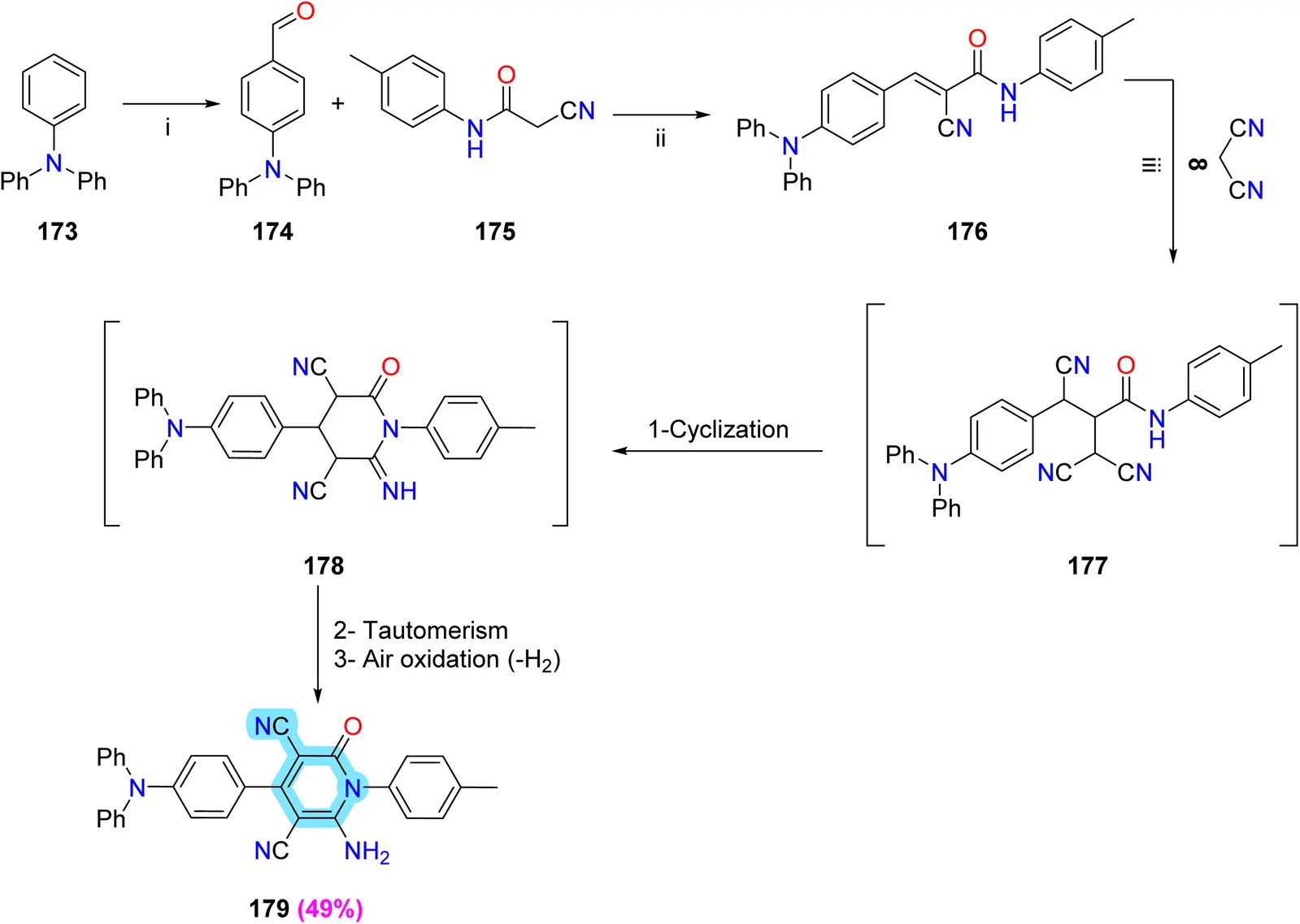
Synthesis of compounds 179; reagents and conditions: (i) DMF (25 eq.), POCl_3_ (21 eq.), r.t; (ii) EtOH, piperidine, reflux, 2 h.; (iii) EtOH, piperidine, reflux, 6 h.

Compound 179 was tested against two cell lines: A-549 and MDA-MB-231. 179 displayed the highest values of IC_50_ (0.00803 and 0.0103 µM), respectively, in comparison with cisplatin as the reference drug (IC_50_ = 0.0244 and 0.012 µM, respectively). Moreover, the molecular docking study performed ensured the outstanding anticancer activities of 179.^101^

S. Y. Mansour *et al.*, investigated the synthesis and anticancer assessment of some new 2-amino-3-cyanopyridine derivatives. Initially, the reaction of acetophenone 122, anisaldehyde 180, and malononitrile 8 in the presence of ammonium acetate under microwave conditions using one-pot reaction produced 2-amino-4-(4-methoxyphenyl)-6-phenylnicotinonitrile 181. Subsequently, the latter 181 was reacted with acetic anhydride 182, giving the monoacylated product 183, which was then subjected to react with *o*-phenylene diamine 184 to afford the desired imidazolo nicotinonitrile derivative 185 (yield: 62%)^102^Scheme 36.

**Scheme 36:**
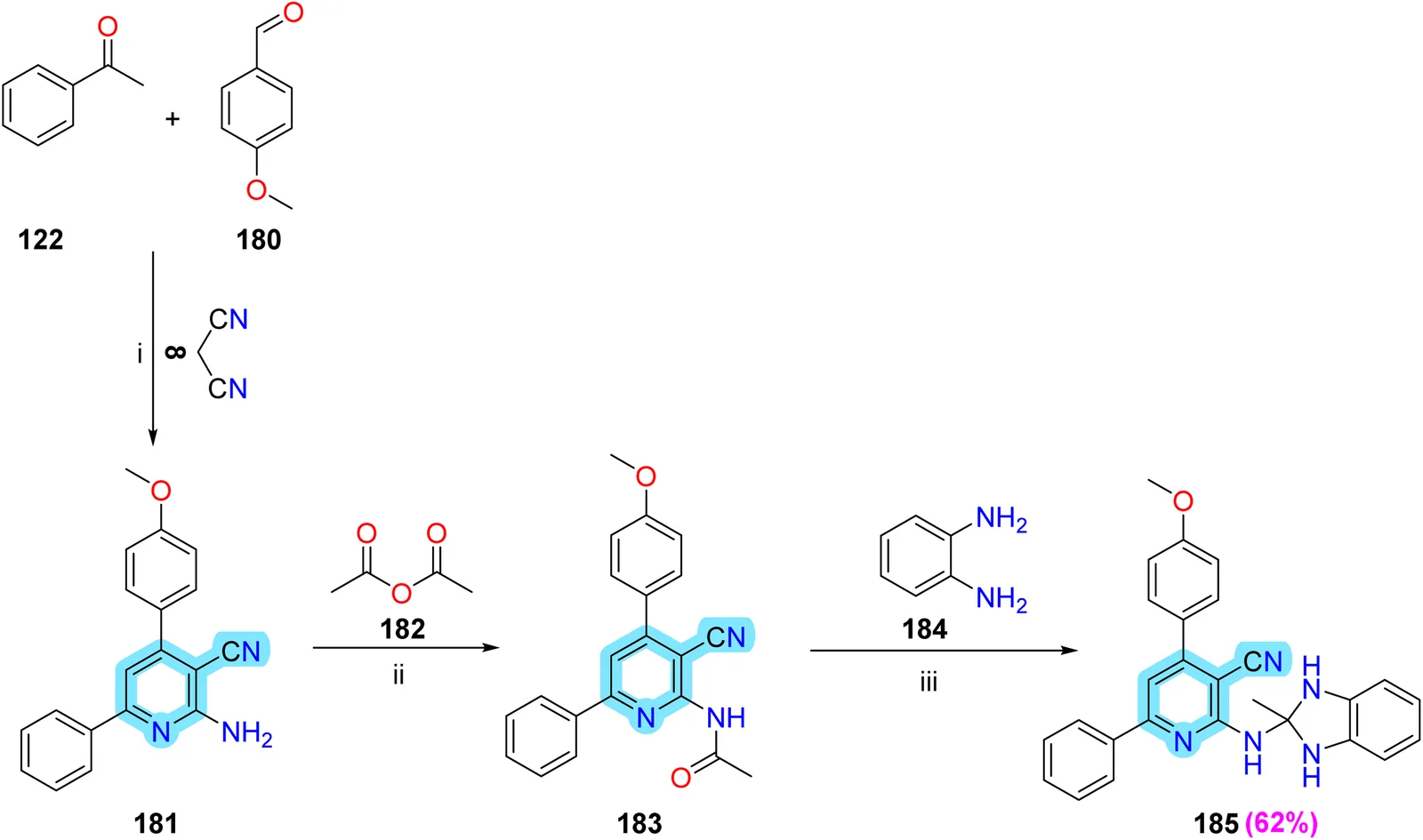
Synthesis of compounds 185; reagents and conditions: (i) AcONH_4_, M.W, 7 min.; (ii) Method (a) reflux, 9 h, Method (b) M.W, 3 min.; (iii) EtOH, reflux, 6 h.

Compound 185 was evaluated for its *in vitro* cytotoxic effect against HepG2 and MCF-7 cancer cell lines. 185 showed very strong cytotoxic activities against HepG-2 and MCF-7 cell lines with IC_50_ of 7.48 ± 0.6 and 5.69 ± 0.4 µM, respectively.^102^

Safaa I. Elewa *et al.* reported the synthesis and anticancer activity of new pyridine-3-carbonitrile derivatives. First, pyridine-3-carbonitrile derivative 186 was synthesized by the condensation reaction of a mixture of benzaldehyde 33, 2-acetylthiophene 20, and ethyl cyanoacetate 18 in boiling ethanol in the presence of ammonium acetate. Then, the reaction of 186 with ethyl chloroacetate 2 in ethanol afforded 2-[(ethoxycarbonyl)methyloxy]-4-phenyl-6-(2-thienyl)pyridine-3-carbonitrile 187. Additionally, the reaction of 187 with hydrazine hydrate gave pyridine-3-carbonitrile derivative 188. Finally, 188 was reacted with nitrous acid to give the target nicotinonitrile derivative 189 (yield: 80%)^103^Scheme 37.

**Scheme 37:**
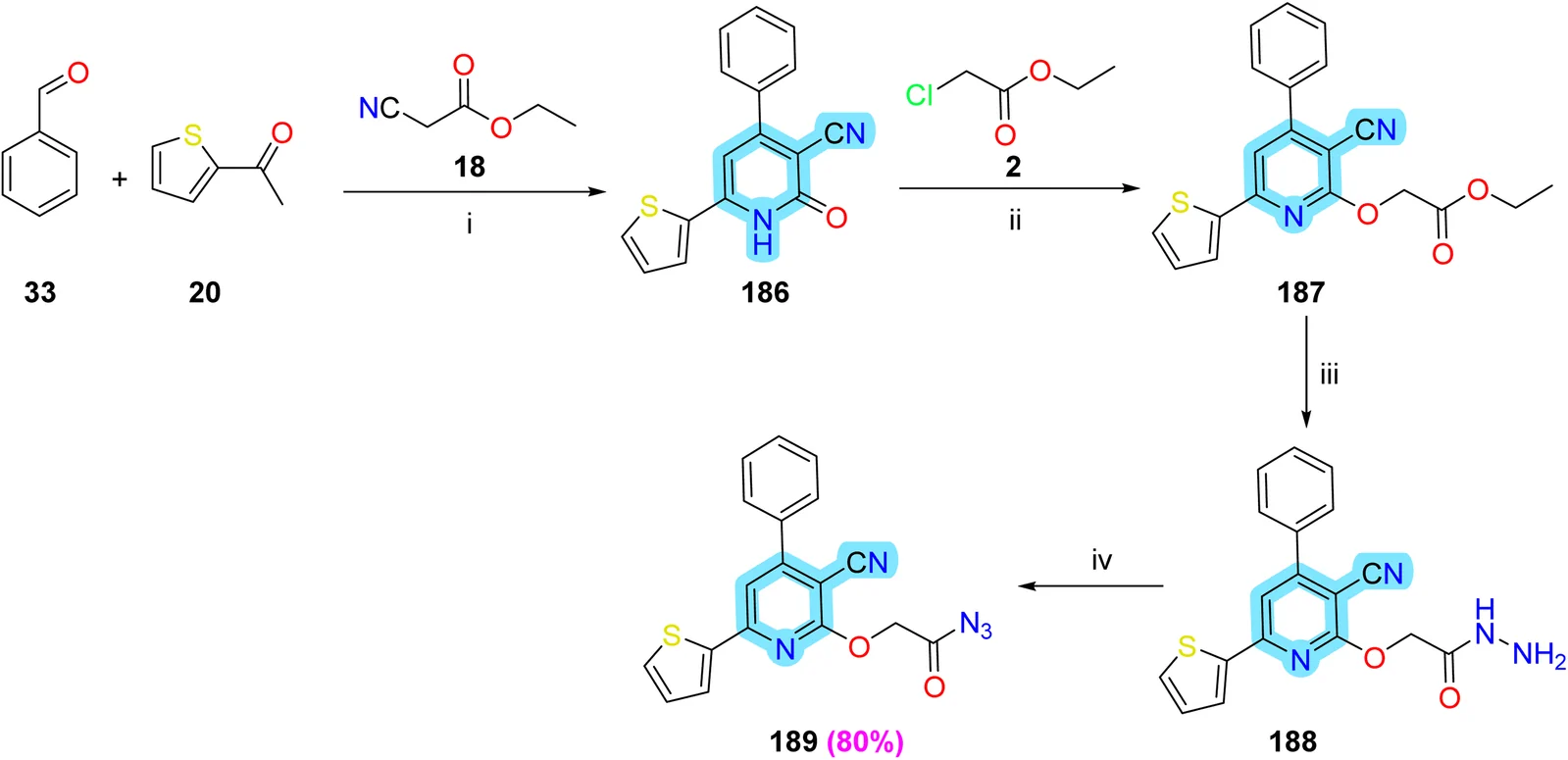
Synthesis of compound 189; reagents and conditions: (i) AcONH_4_, EtOH, reflux, 1 h.; (ii) K_2_CO_3_, DMF, r.t, 20 h; (iii) N_2_H_4_·H_2_O, EtOH, reflux, 6 h; (iv) NaNO_2_, AcOH, stirring, r.t.

Compound 189 showed anti-proliferative activity toward HePG-2, MCF-7, HCT-116 cancer cell lines with IC_50_ values of 19.85 ± 1.4, 23.09 ± 2.3 and 29.17 ± 1.9 µg mL^−1^, respectively, using the standard drug DOX.^103^

Hebat-Allah S. Abbas and her team investigated efficient synthesis and biological evaluation of new series of pyridine derivatives. The synthetic pathway for the target compound 194 is illustrated in Scheme 38. The first step involved the synthesis of the intermediate 190*via* the reaction of the 4-methyl acetophenone 37 and 4-methylbenzaldehyde 144 followed by reflux with ethyl cyanoacetate 18 and ammonium acetate in ethanol. Next, alkylation of pyridine-3-carbonitrile 191 with ethyl bromoacetate 87 in dry acetone in the presence of anhydrous K_2_CO_3_ afforded the corresponding pyridine derivative 192. Moreover, hydrazinolysis was carried out by the reaction of pyridine derivative 192 with hydrazine hydrate in refluxing ethanol, which furnished the desired acetohydrazide 193. Finally, refluxing of acetohydrazide 193 with formic acid resulted in the formation of the desired 194 (yield: 65%).^104^

**Scheme 38:**
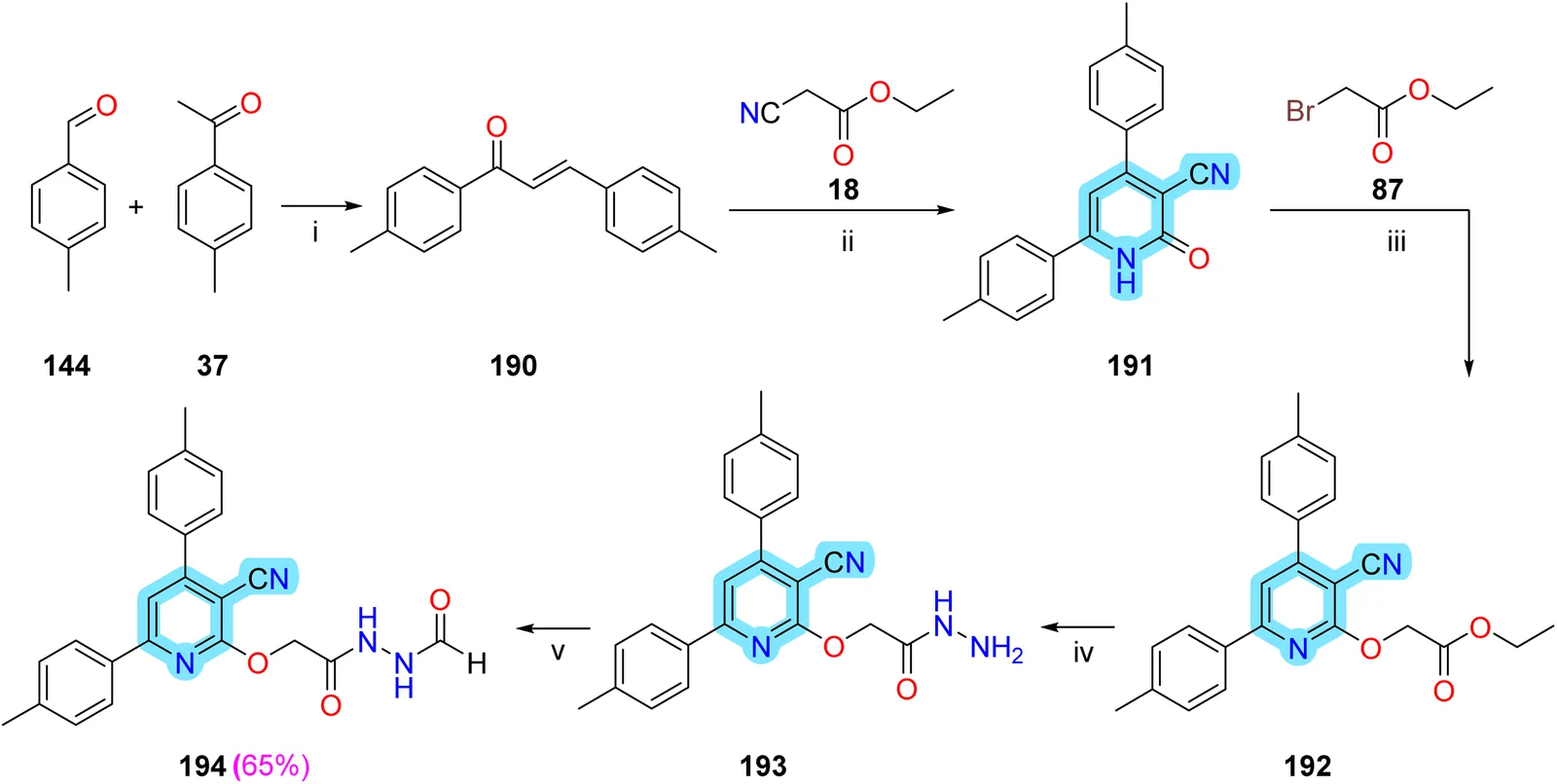
Synthesis of compounds 194; reagents and conditions: (i) NaOH, EtOH, r.t, 4 h; (ii) AcONH_4_, EtOH, reflux, 6 h; (iii) K_2_CO_3_, acetone, reflux, 12–16 h.; (iv) N_2_H_4_·H_2_O, EtOH, reflux, 16–18 h; (v) HCOOH, reflux, 110–115 °C, 6 h.

Compound 194 was screened against three tumor cell lines; MCF-7, HCT-116, and SKOV3 with IC_50_ values of 0.7, 0.5, and 0.1 µM, respectively, which exceeded the reference drug DOX (IC_50_ = 1.9, 1.8 and 2.1 µM), respectively.^104^

Mehmet Erşatır and Metin Yıldırım developed green synthesis of 2-amino-3-cyanopyridines and their antiproliferative effects on liver, breast, and prostate cancer studies. The target 2-amino-3-cyanopyridine derivative 195 was synthesized in a short time and with 99% yield in an ultrasonic bath without using solvent and catalyst in a single pot multi-component reaction of *p*-nitrobenzaldehyde 146b, acetophenone 122, malononitrile 8, and ammonium acetate^105^Scheme 39.

**Scheme 39:**
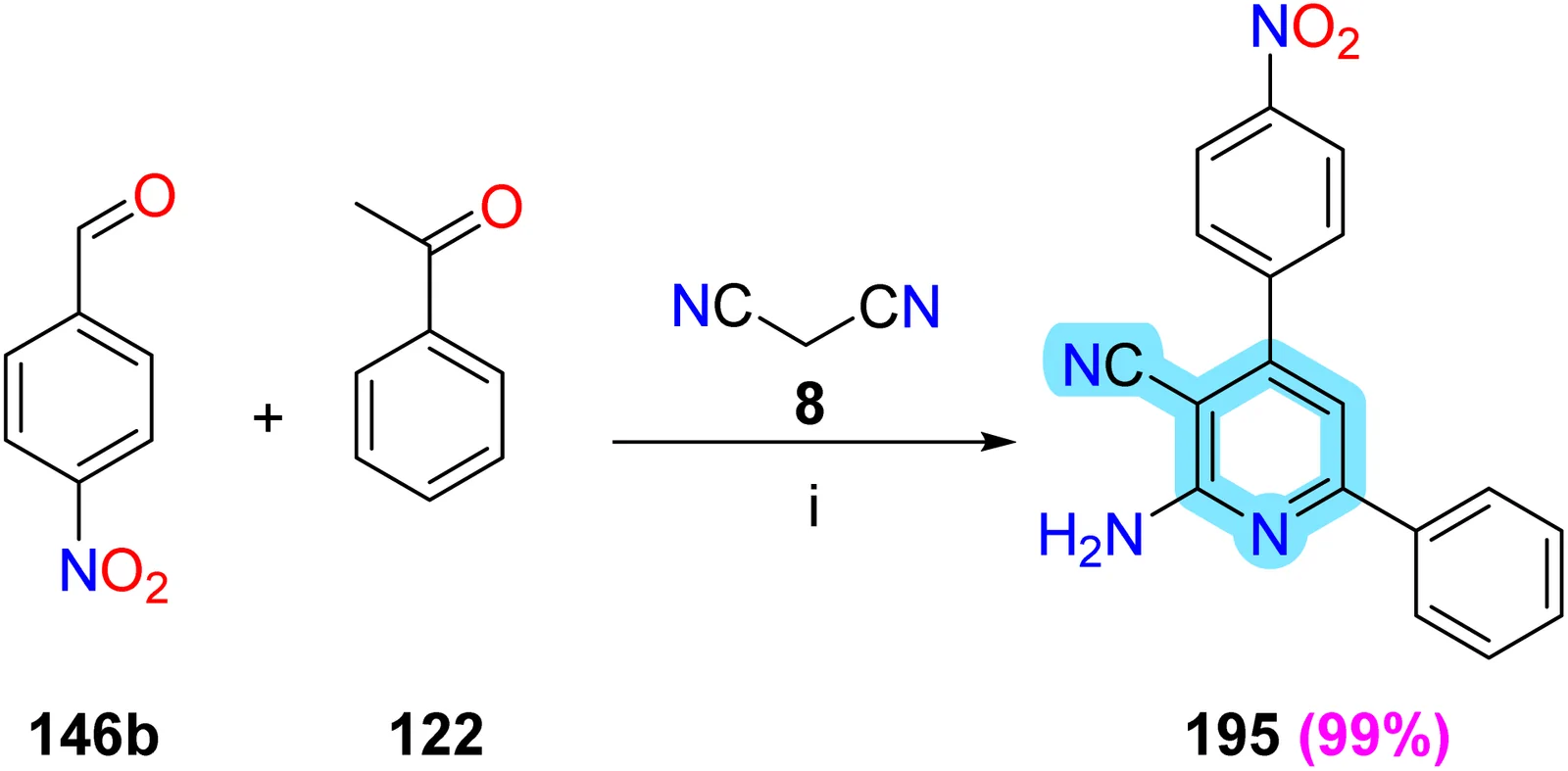
Synthesis of compound 195; reagents and conditions: (i) AcONH_4_, r.t, 5 min.

Compound 195 was tested for its anticancer activity against MCF-7, HepG2, and DU-145 cell lines. 195 showed better antiproliferative effects on the MCF-7 and HepG2 cells with IC_50_ values of 1.8 and 7.7 µM, respectively, than DOX, which was used as a control with IC_50_ values of 5.93 and 8.53 µM, respectively.^105^

Amira A. El-Sayed and her colleagues reported the synthesis and antiproliferative activity of new substituted pyridine candidates. Initially, one-pot multi-component reaction of 4-chlorobenzaldehyde 25, 1-(naphthalen-1-yl)ethanone 196, and ethyl cyanoacetate 18 in the presence of a catalytic amount of ammonium acetate and drops of piperidine afforded 4-(4-chlorophenyl)-6-(naphthalen-1-yl)-2-oxo-1,2-dihydropyridine-3-carbonitrile 197. Additionally, chlorination of compound 197 with POCl_3_ afforded 2-chloropyridine derivative 198 upon the reaction of the latter 198 with hydrazine hydrate, 2-hydrazinyl nicotinonitrile 199. Finally, the reaction of the hydrazinyl derivative 199 with phenylacetyl chloride 200 afforded the nicotinonitrile derivative 202 (yield: 33%)^106^Scheme 40.

**Scheme 40:**
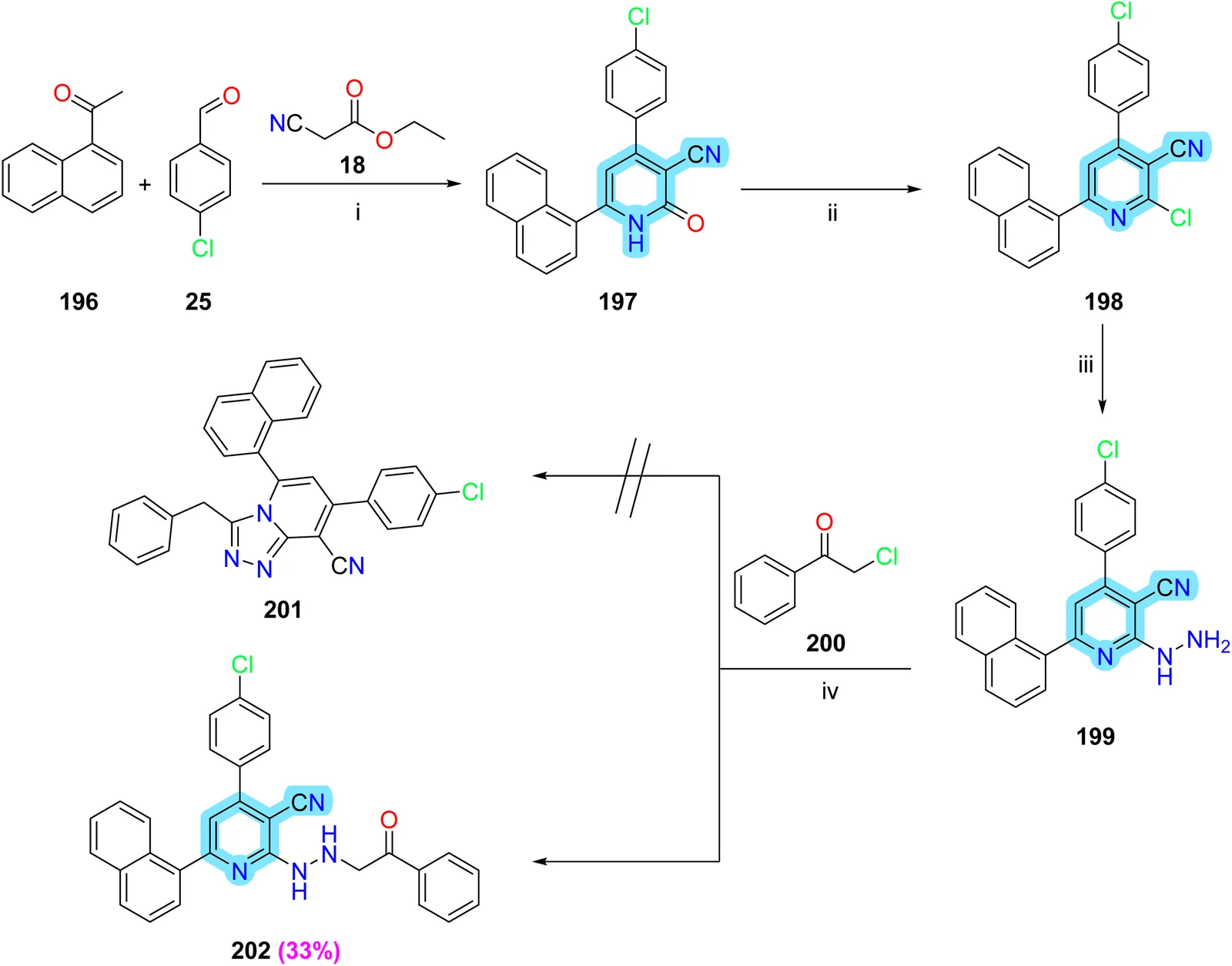
Synthesis of compounds 202; reagents and conditions: (i) AcONH_4_, piperidine (drops), EtOH, reflux, 2 h.; (ii) PCl_5_, POCl_3_, reflux,4 h; (iii) N_2_H_4_·H_2_O, EtOH, reflux, 2 h; (iv) dry pyridine, stirring, 0 °C, 0.5 h.

Compound 202 was tested against cancer cell lines, including U937, SKMEL-28, NCIH 460, RKOP 27, and HeLa cancer cell lines. 202 showed the highest antiproliferative effects among all the tested derivatives, where it had IC_50_ values of 25 ± 2.6, 16 ± 2, 127 ± 25, 422 ± 26, and 255 ± 2 nM against NCIH 460, RKOP 27, HeLa, U937, and SKMEL 28 cells, respectively, in comparison to all reference drugs used.^106^

Reham R. Raslan and her team reported the synthesis and antitumor evaluation of some new nicotinonitrile derivatives. The synthetic route for the target 204 is outlined in Scheme 41. One-pot four-component reaction of cyclohexanone 203 with 4-hydroxybenzaldehyde 75, malononitrile 8, and ammonium acetate in the presence of ceric ammonium nitrate (CAN) afforded the desired derivative 204 (yield: 65%).^107^

**Scheme 41:**
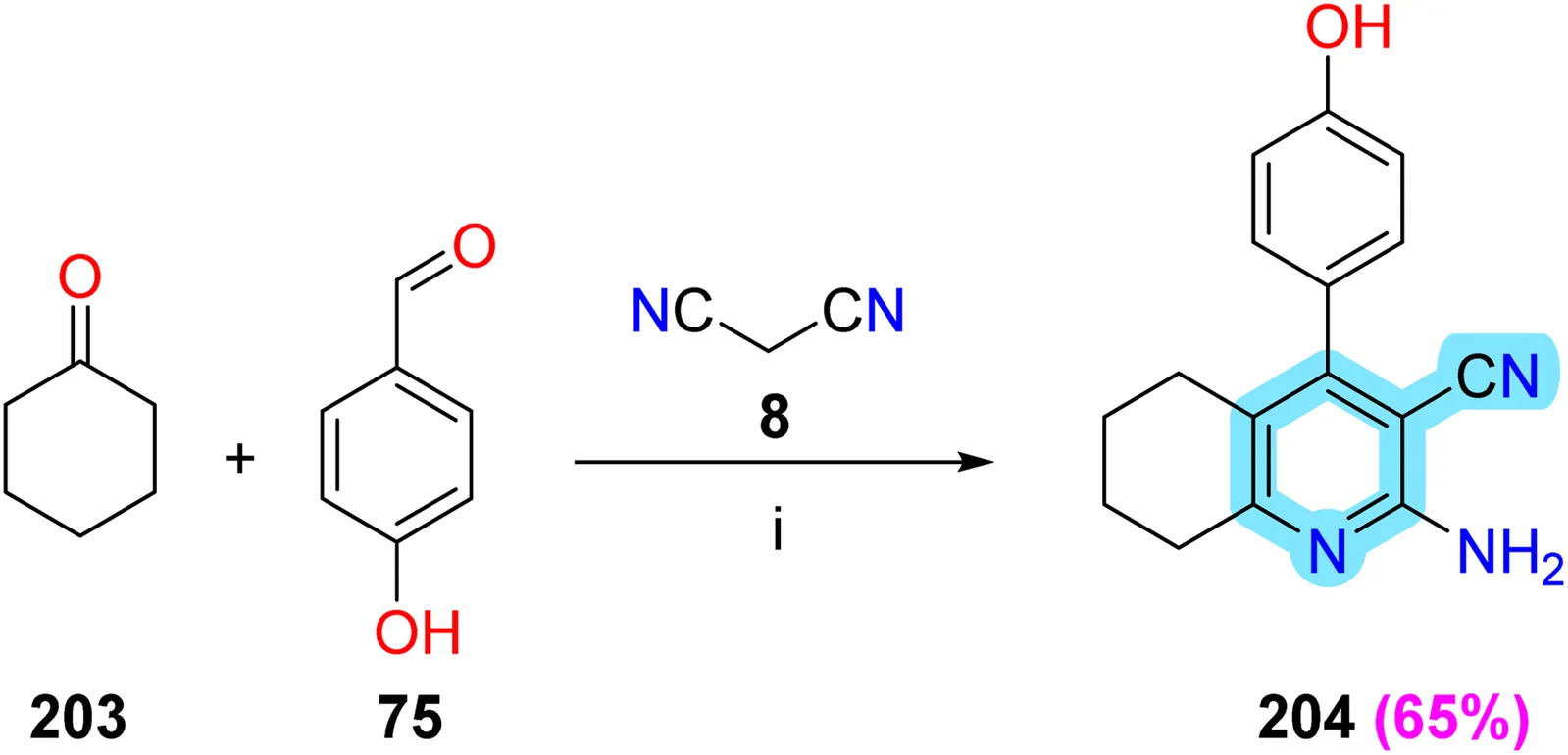
Synthesis of compounds 204; reagents and conditions: (i) AcONH_4_, CAN 5% mol, EtOH, reflux, 1 h.

The cytotoxicity of compound 204 was investigated against HePG-2 and colorectal Caco-2 cell lines. 204 exhibited excellent cytotoxic activity with IC_50_ values of 5.86 and 11.47 µM, respectively, compared to the standard drug DOX (IC_50_ values of 4.50 and 12.49 µM, respectively).^107^

New derivatives of heterocyclic bearing pyrazole moiety were synthesized *via* green synthesis methods by Kurls E. Anwer *et al.* The synthesis of the target 207 is straightforward and illustrated in Scheme 42. The synthesis involved the irradiation of bicyclic bifunctional starting material 205 with malononitrile 8 in the presence of sodium ethoxide, producing the desired pyrazolopyridine 207 (yield: 96.7%).^108^

**Scheme 42:**
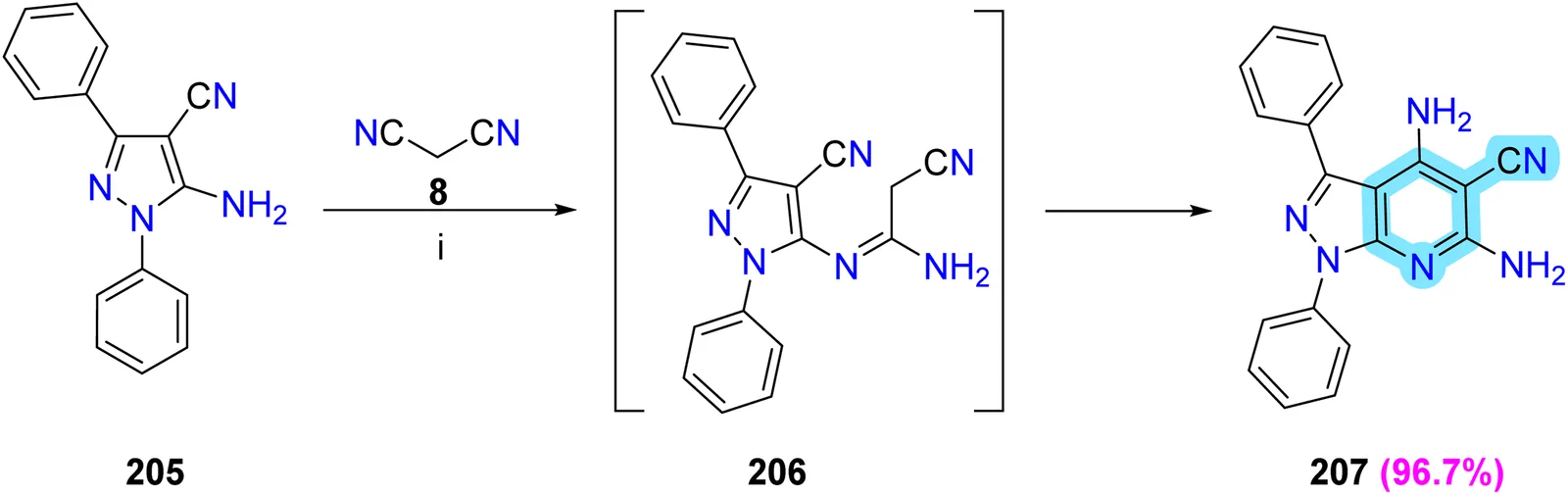
Synthesis of compounds 207; reagents and conditions: (i) EtONa, M.W, 2 min.

Compound 207 was *in vitro* evaluated for its cytotoxicity against two human cancer cell lines (HepG2 and HCT-116). 207 had the highest cytotoxic effects against the two assessed cell lines with IC_50_ values of 9.2 ± 2.8 and 7.7 ± 1.8 µM against HepG2 and HCT-116, respectively, compared with 5-FU as a ref. 108.

Table 2 summarizes the biological data of several recently synthesized nicotinonitrile derivatives with anticancer activity, detailing their key molecular targets, melting points, and cytotoxicity profiles.

Summary of reported biochemical, cellular, and computational activities of selected nicotinonitrile-based anticancer derivatives with kinase inhibition

**Table:** 

Scheme	Structure	m.p. (°C)	Kinase inhibition activity			Anticancer activity			Ref.
1	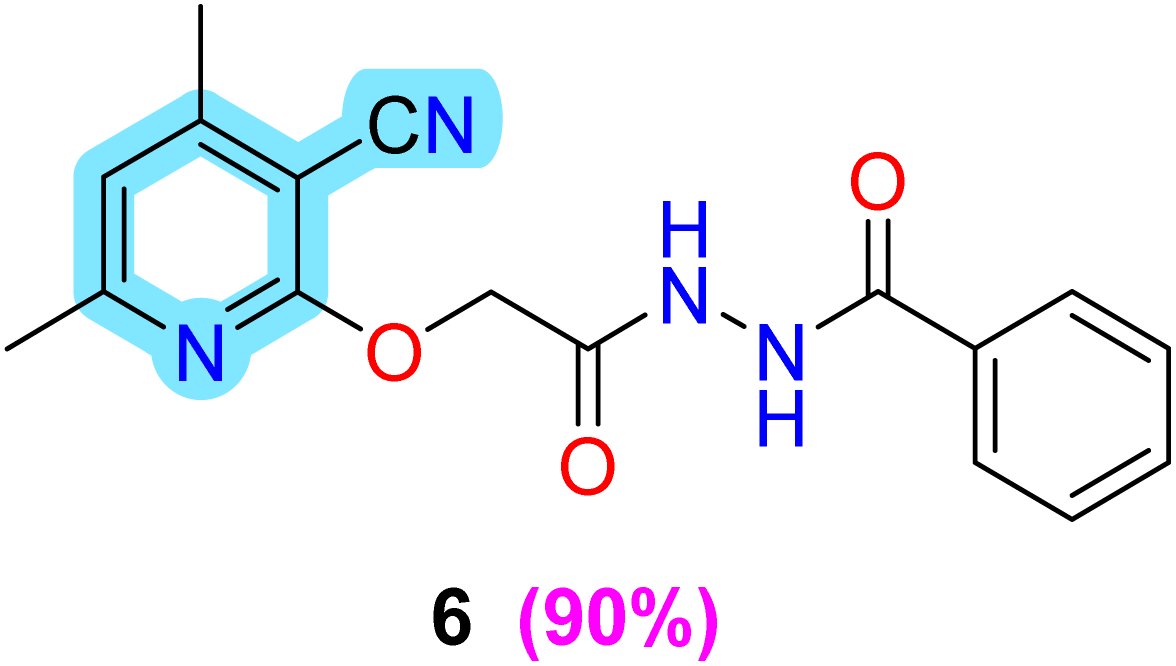	212	Enzymes	IC_50_ (nM)		Cell lines	IC_50_ [µM]		31
				6	Erlotinib		6	DOX	
			EGFR	42.6 ± 1.9	78.65 ± 2.1	A549	0.85 ± 0.03	3.07 ± 0.12	
						PC-3	3.63 ± 0.16	2.23 ± 0.1	
						WI38	59.88 ± 2.6	17.94 ± 0.78

**Table:** 

Scheme	Structure	m.p. (°C)	Kinase inhibition activity			Anticancer activity				Ref.
2	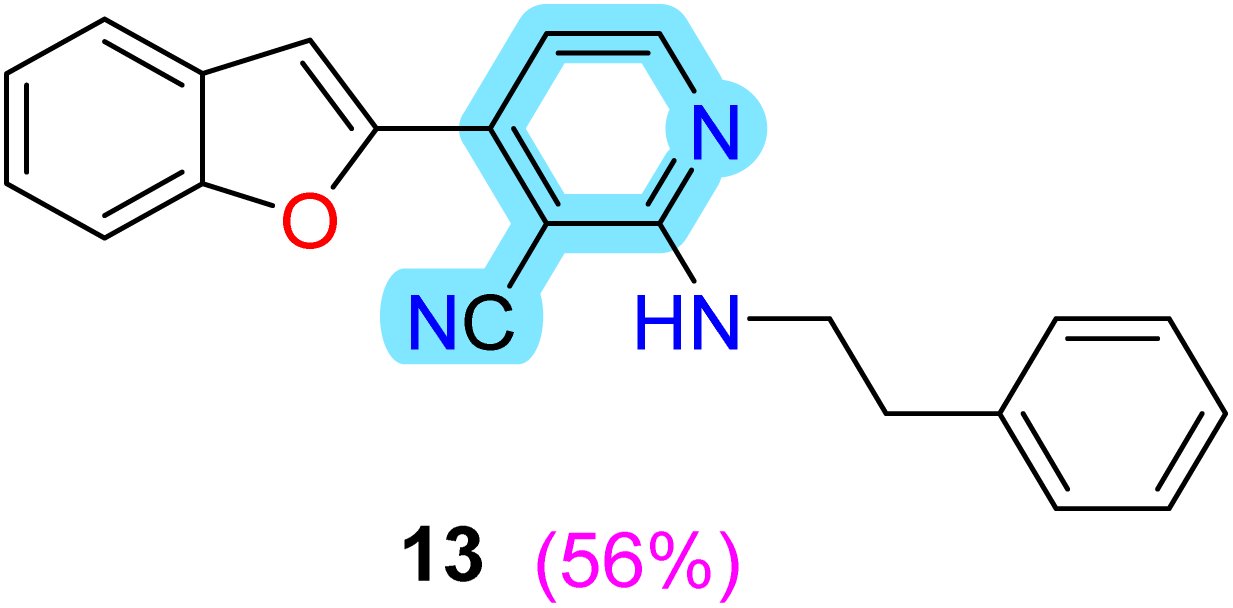	157–159	Enzymes	IC_50_ [µM]		Cell lines	IC_50_ [µM]			72
				13	Gefitinib		13	Afatinib	DOX	
			EGFR	0.81	0.90	HePG2	20.43 ± 1.6	5.5 ± 0.24	4.50 ± 0.2	
						HCT-116	46.13 ± 2.7	11.2 ± 1.11	5.23 ± 0.3	
						MCF-7	14.55 ± 1.3	7.2 ± 0.50	4.17 ± 0.2	
						PC-3	18.75 ± 1.5	7.6 ± 0.60	8.87 ± 0.6	
						HeLa	32.15 ± 1.9	6.3 ± 0.69	5.57 ± 0.4

**Table:** 

Scheme	Structure	m.p. (°C)	Kinase inhibition activity			Anticancer activity			Ref.
3	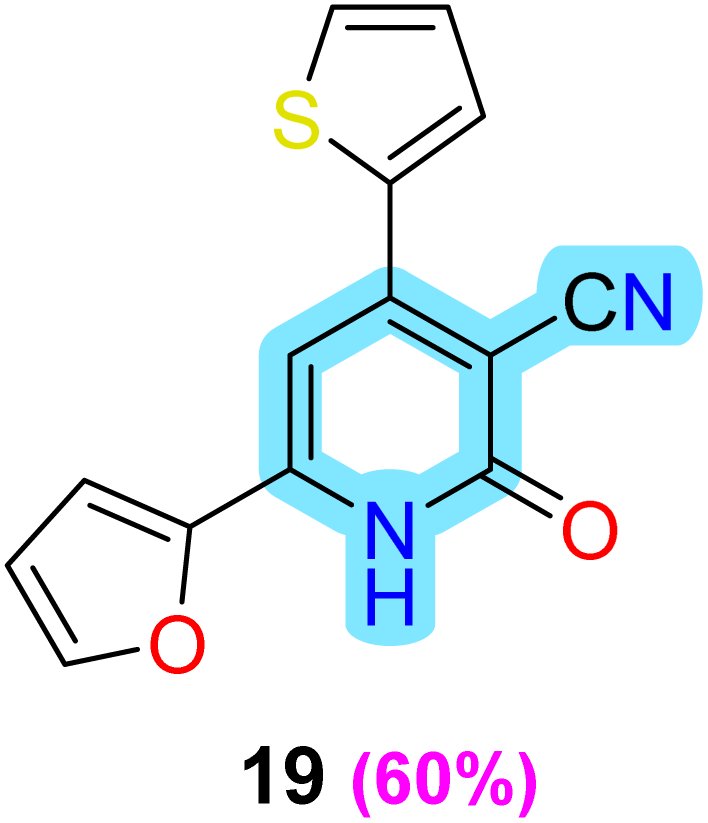	300–303	Enzymes	IC_50_ [µM]		Cell lines	IC_50_ µg mL^−1^		74
				19	Erlotinib		19	DOX	
			EGFR wild	0.203 ± 0.012	0.042 ± 0.003	MCF7	3.70 ± 0.14	4.5 ± 1.5	
						T47D	4.70 ± 0.29	4.0 ± 1.1	
						VERO	4.60 ± 0.40	23.0 ± 3.0	
						H460	8.0 ± 0.70	6.5 ± 1.40	
						FADU	3.90 ± 0.14	7.0 ± 1.60	
			EGFR T790M	0.156 ± 0.009	0.009 ± 0.001	HEP2	4.70 ± 0.42	5.60 ± 1.20	
						HEPG2	3.80 ± 0.21	4.58 ± 1.20	
						HCT116	3.90 ± 0.14	5.5 ± 1.60	
						CaCo2	4.10 ± 0.21	6.0 ± 1.80

**Table:** 

Scheme	Structure	m.p. (°C)	Kinase inhibition activity		Anticancer activity			Ref.
4	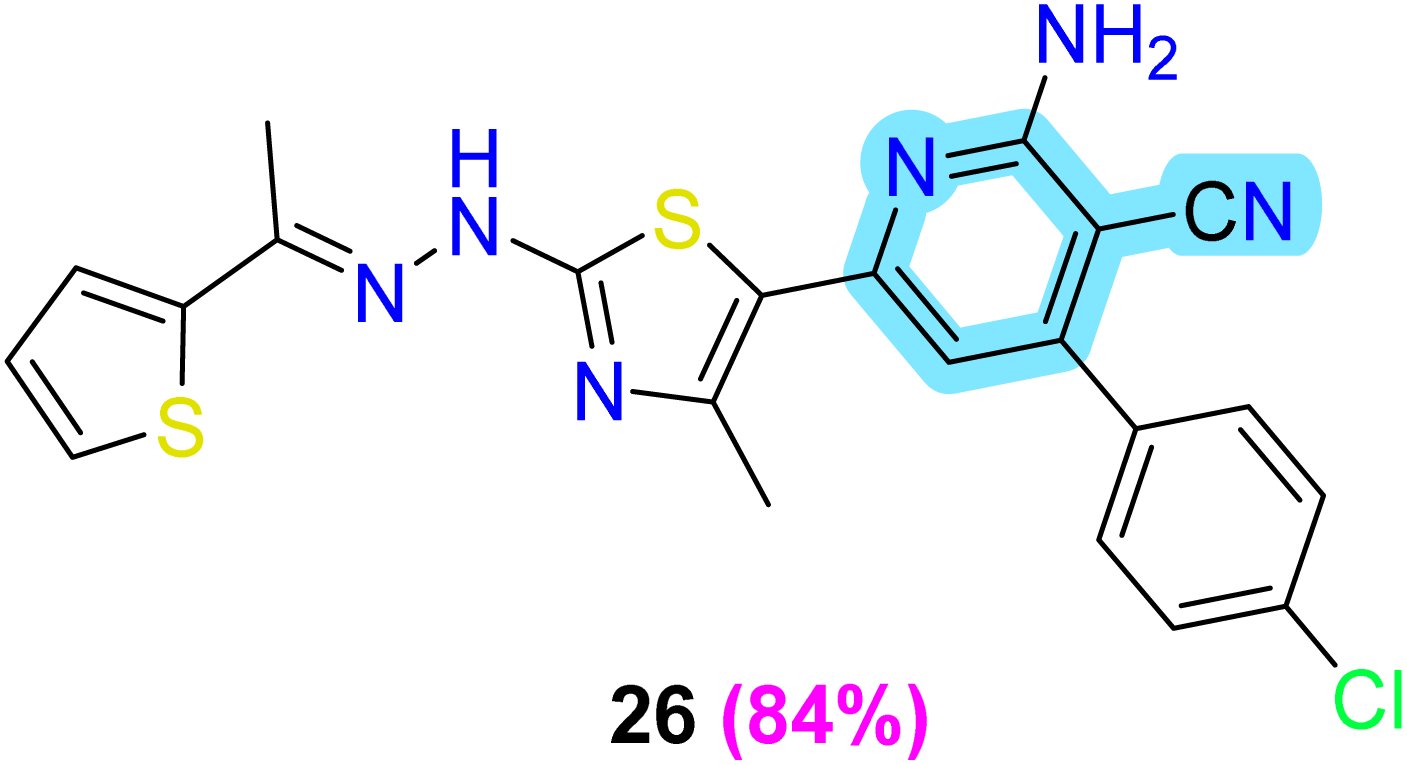	199–201	Enzymes	Docking study	Cell lines	IC_50_ [µM]		75
				26		26	DOX	
			EGFR	26 displayed the best docking score of −24.45	A549	0.302	0.460

**Table:** 

Scheme	Structure	m.p. (°C)	Kinase inhibition activity		Anticancer activity			Ref.
5	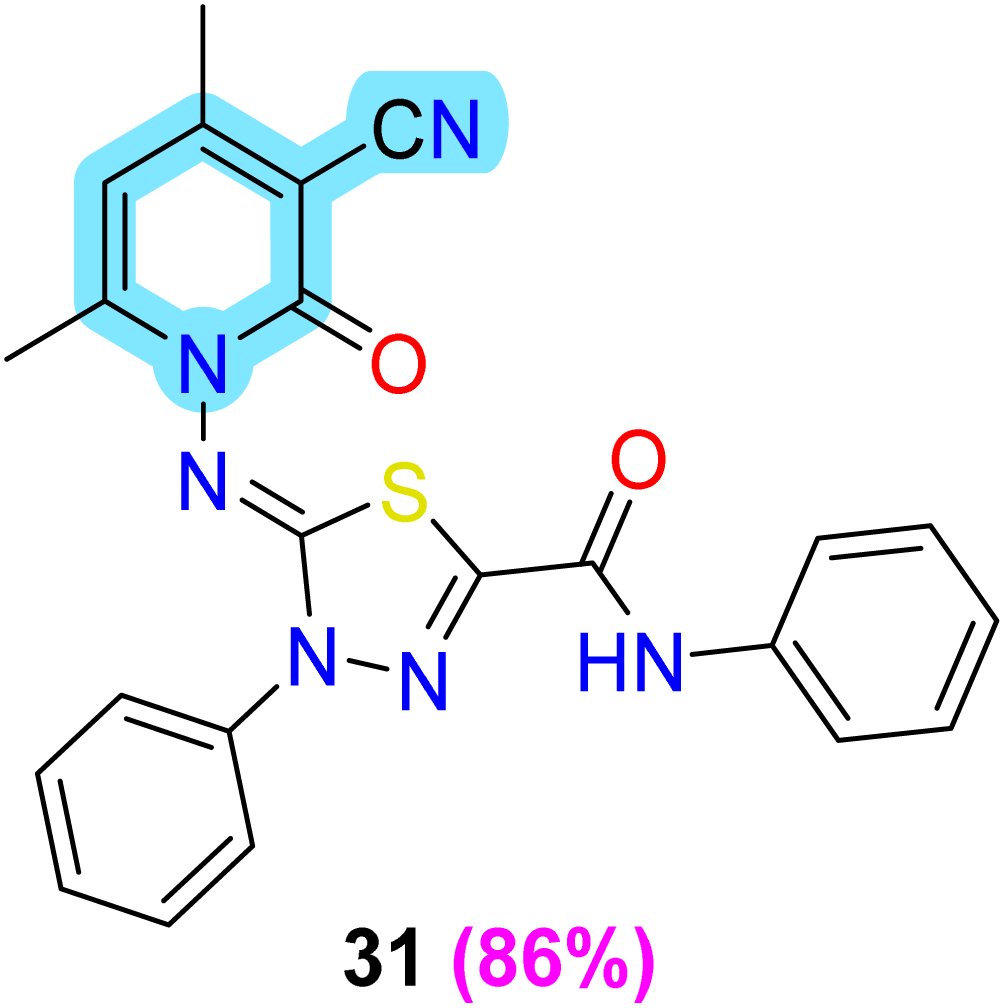	277–279	Enzymes	Docking study	Cell lines	IC_50_ [µM]		76
				31		31	Harmine	
				31 displayed the best docking score of −10.8	HCT-116	2.03 ± 0.72	2.40 ± 0.12	
					HepG2	2.17 ± 0.83	2.54 ± 0.82	
					A-2780	0.077	0.138

**Table:** 

Scheme	Structure	m.p. (°C)	Kinase inhibition activity		Anticancer activity			Ref.
6	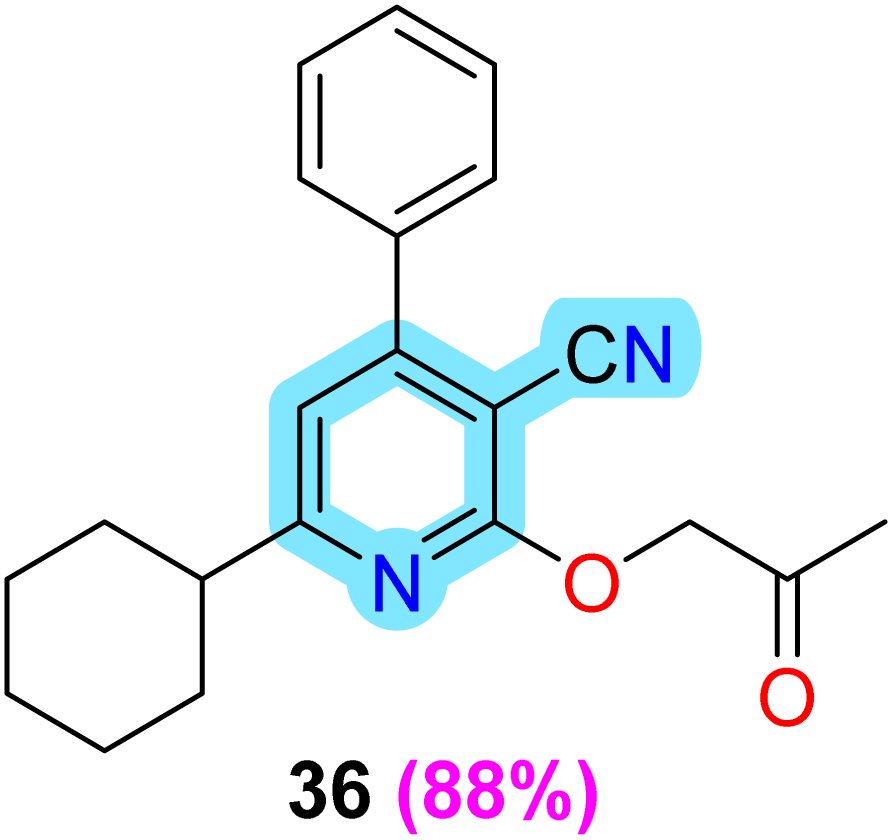	138–139	Enzymes	Docking study	Cell lines	IC_50_ [µM]		44
				36		36	DOX	
			EGFR	36 displayed the best docking score of −8.14	A549	7.3	49.3	
					PACA2	29.8	48.8

**Table:** 

Scheme	Structure	m.p. (°C)	Kinase inhibition activity		Anticancer activity				Ref.
7	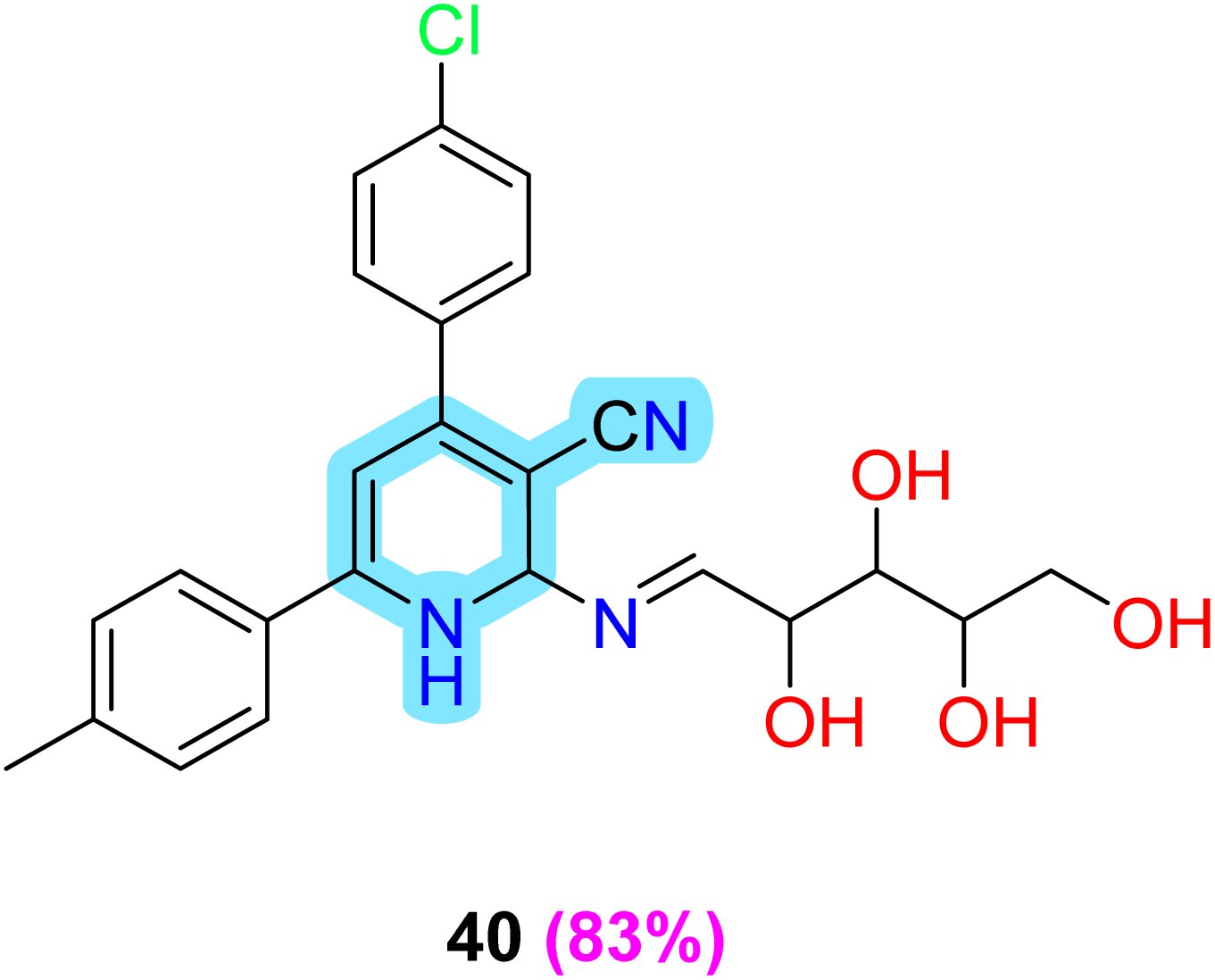	>300	Enzymes	Docking study	Cell lines	IC_50_ [µM]			77
				40 and 44		40	44	DOX	
			VEGFR-2	Compounds 40 and 44 displayed the best docking score of −40.73 and −59.11	HCT-116	4.1 ± 0.3	4.1 ± 0.2	4.1 ± 0.3	
	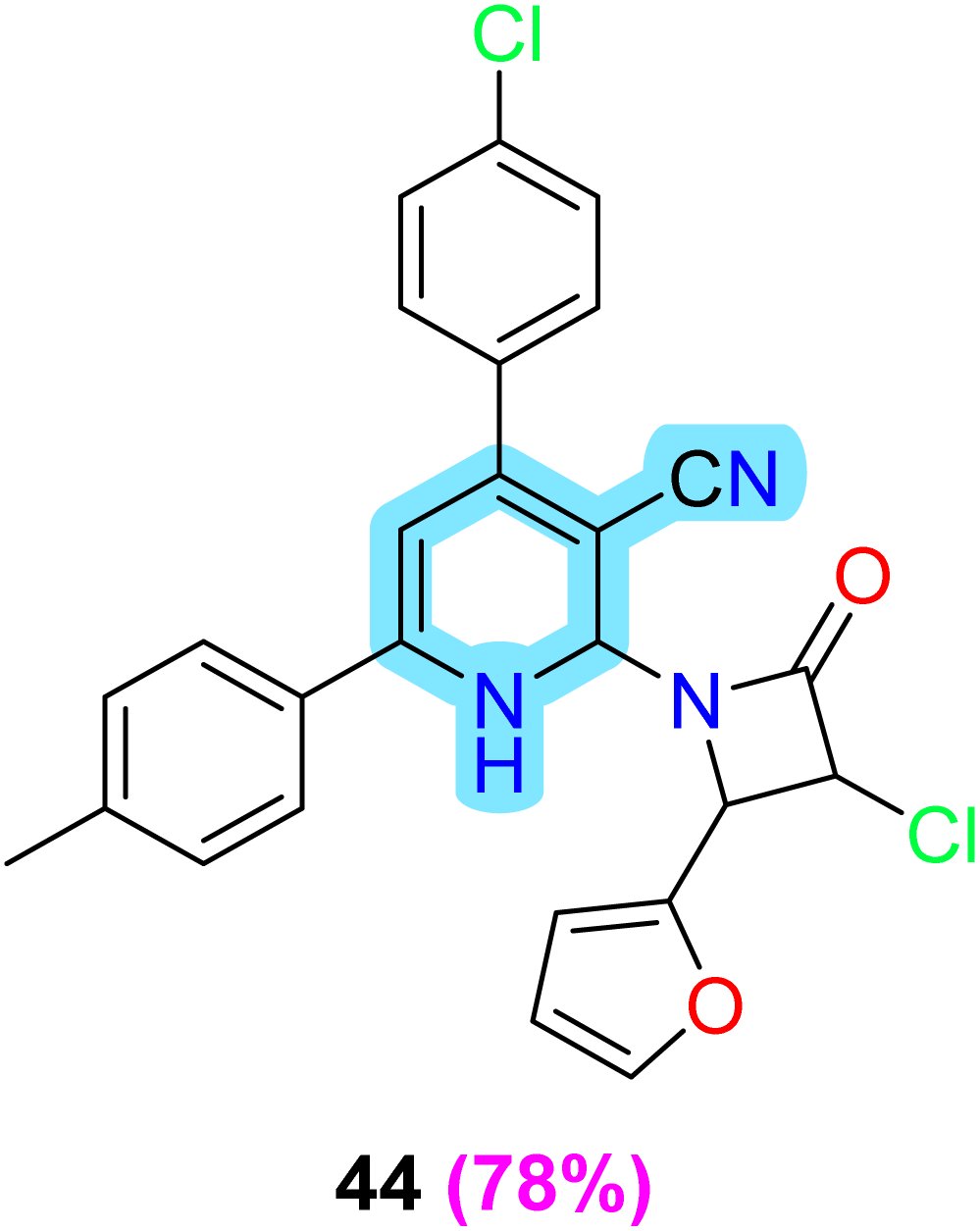	256–258			HepG-2	3.6 ± 0.1	5.0 ± 0.4	5.7 ± 0.3	
					MCF-7	4.0 ± 0.2	3.9 ± 0.1	5.2 ± 0.4

**Table:** 

Scheme	Structure	m.p. (°C)	Kinase inhibition activity			Anticancer activity			Ref.
8	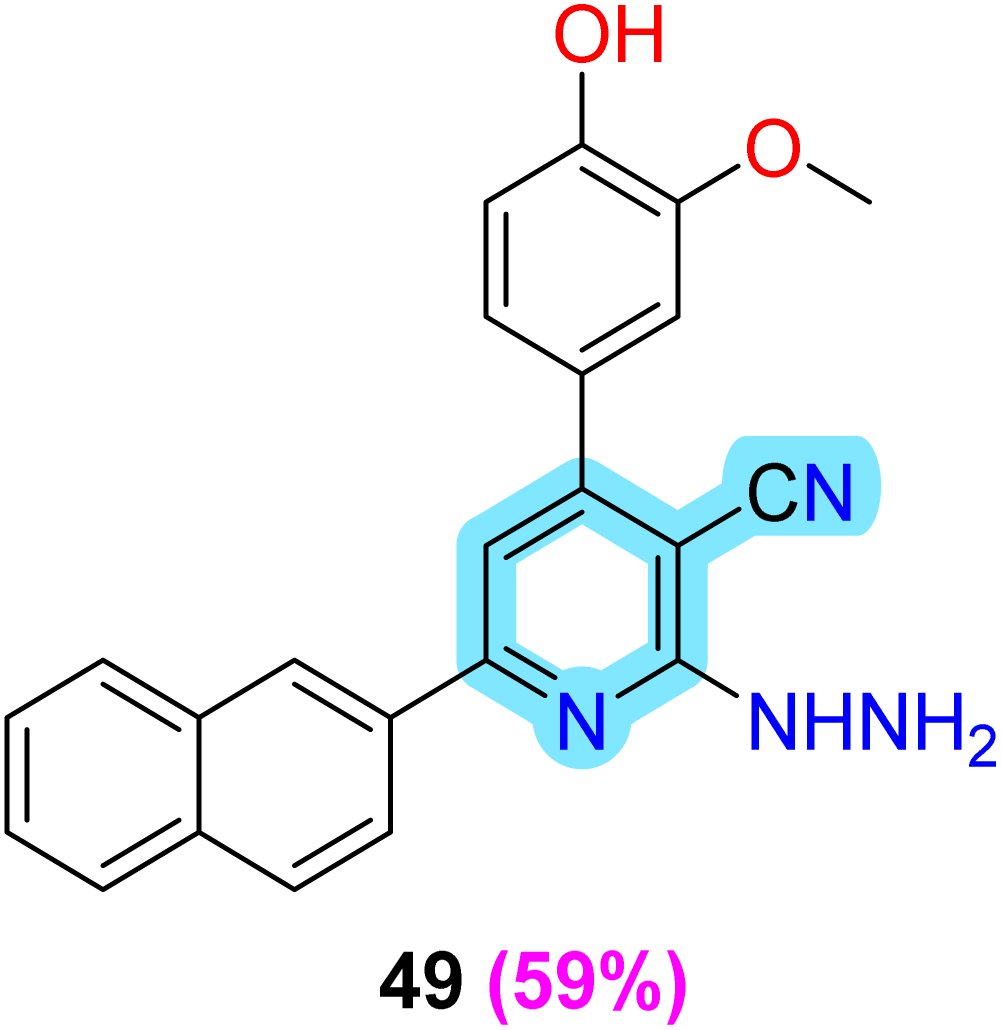	296–298	Enzymes	IC_50_ (nM)		Cell lines	IC_50_ [µM]		78
							49	DOX	
				49	Delphinidin	PC3	55 ± 3.1	59 ± 2.1	
						DU145	8.5 ± 0.43	1.8 ± 0.01	
			VEGFR-2	0.19 ± 0.01	5.09 ± 0.42	MCF-7	0.5 ± 0.001	11 ± 0.37	
						MDA-MB435	96 ± 1.6	69 ± 1.37

**Table:** 

Scheme	Structure	m.p. (°C)	Kinase inhibition activity			Anticancer activity				Ref.
9	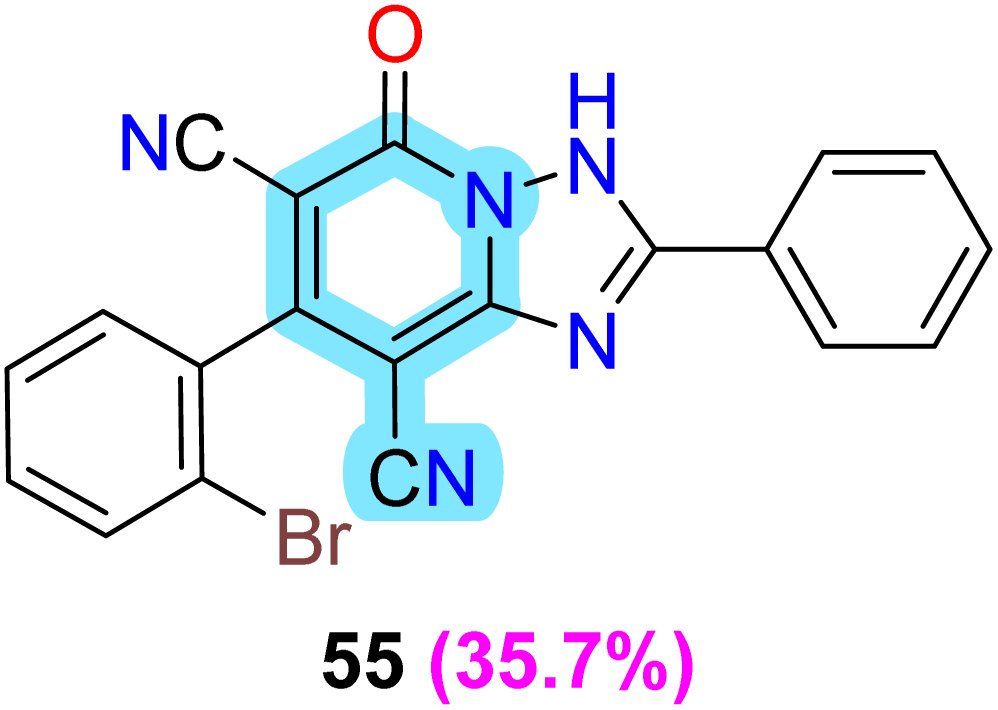	300	Enzymes	IC_50_ (µM)		Cell lines	IC_50_ [µM]			79
				55	Sorafenib		55	Sorafenib	DOX	
			VEGFR-2	0.12 ± 0.01	0.10 ± 0.02	HepG2	4.25 ± 0.03	9.18 ± 0.6	7.94 ± 0.6	
						MCF-7	6.08 ± 0.06	5.47 ± 0.3	8.07 ± 0.8

**Table:** 

Scheme	Structure	m.p. (°C)	Kinase inhibition activity			Anticancer activity		Ref.
10	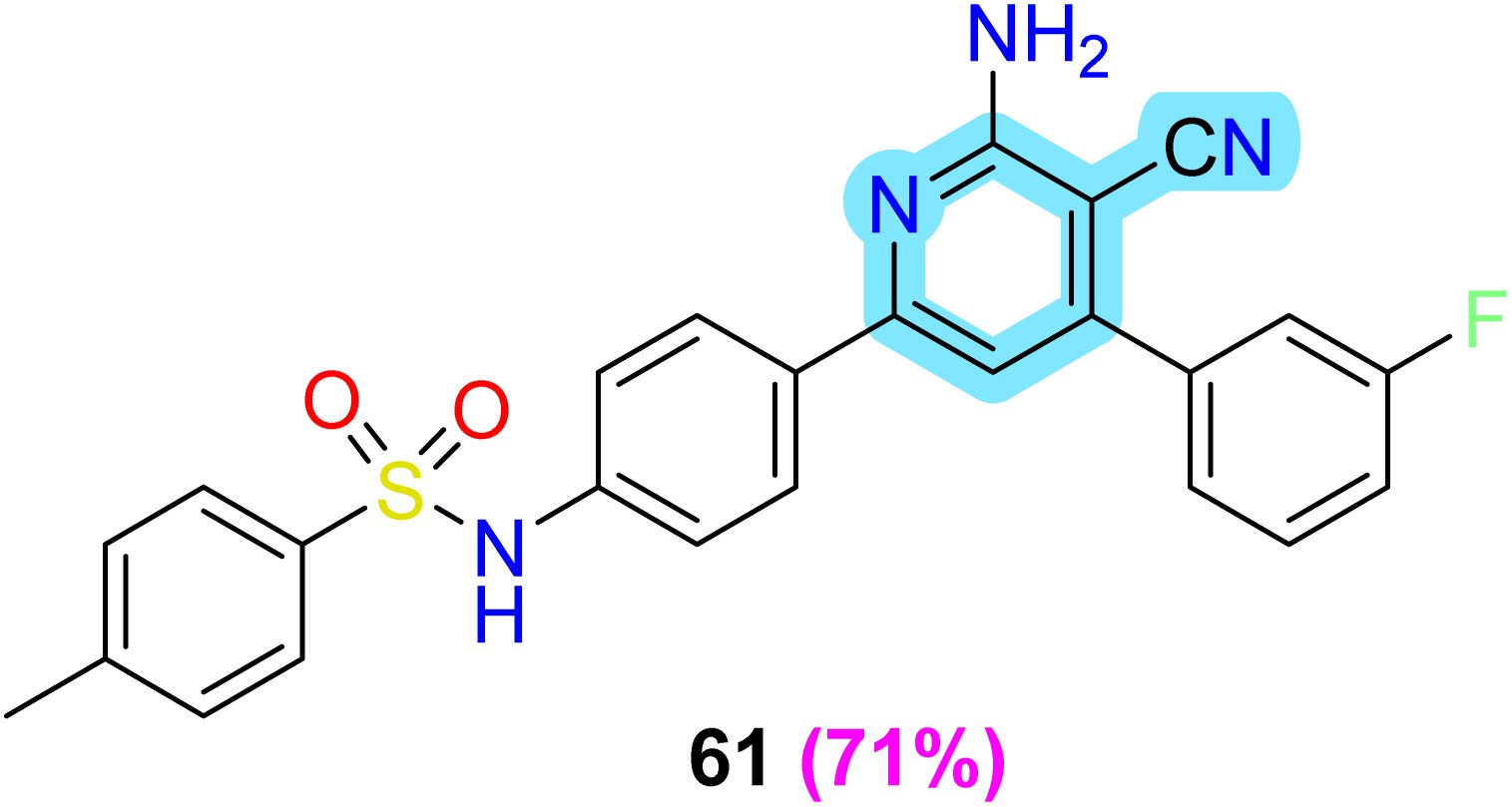	178–180	Enzymes	IC_50_ [µM]		Cell lines	GI_50_ [µM]	80
				61	Sorafenib		61	
			VEGFR-2	3.62 ± 0.04	4.85 ± 0.05	Panel of 60 cell lines	Ranging from 1.06 to 8.92

**Table:** 

Scheme	Structure	m.p. (°C)	Kinase inhibition activity				Anticancer activity				Ref.
11	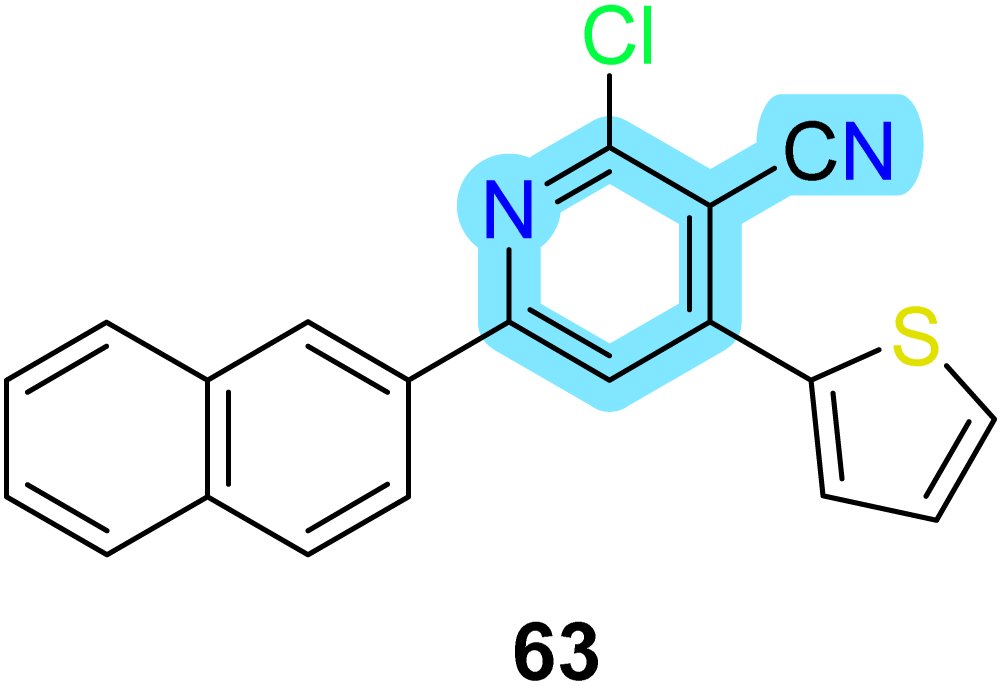	ND	Enzymes	IC_50_ [µM]			Cell lines	IC_50_ [µM]			48
				63	65	Roscovitine		63	65	DOX	
			CDK2	0.236 ± 0.1	0.504 ± 0.1	0.394 ± 0.1	HCT-116	45.9 ± 4.1	31.7 ± 3.5	40.0 ± 3.9	
	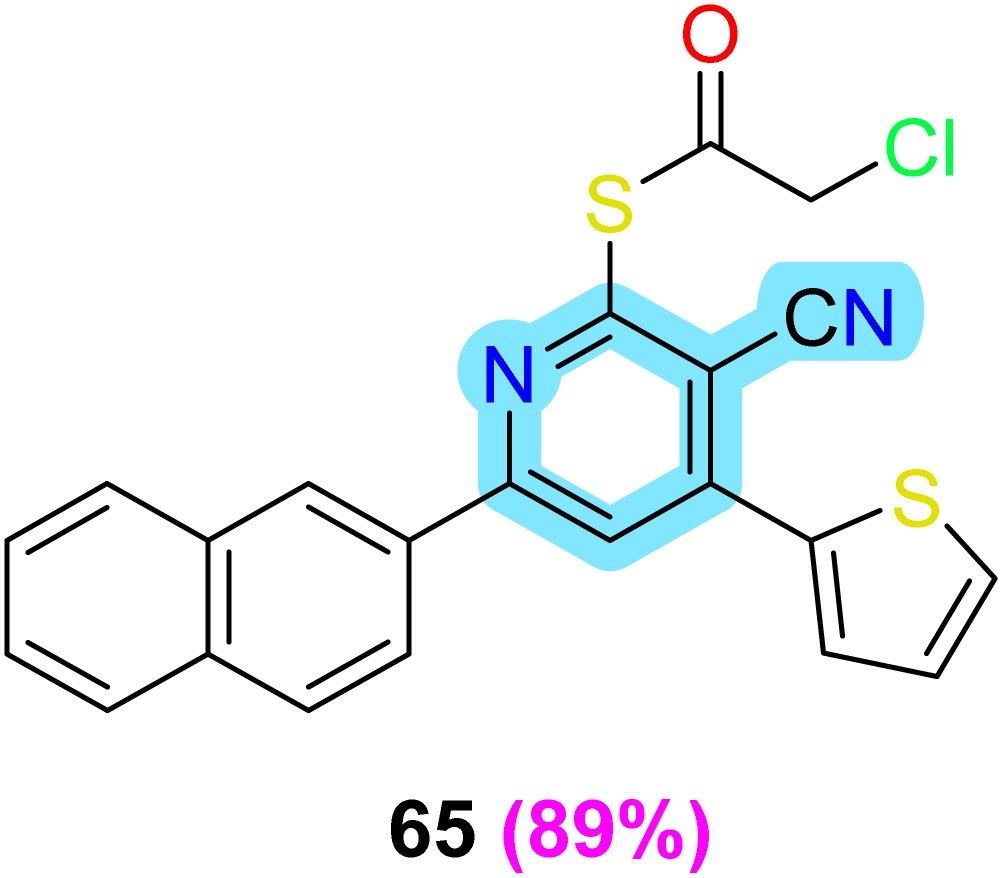	160–161					MCF-7	46.2 ± 4.1	46.4 ± 4.3	64.8 ± 4.1	
							HepG2	42.9 ± 3.9	27.6 ± 3.3	24.7 ± 3.2	
							A549	67.2 ± 5.1	56.9 ± 4.6	58.1 ± 4.1

**Table:** 

Scheme	Structure	m.p. (°C)	Kinase inhibition activity		Anticancer activity			Ref.
12	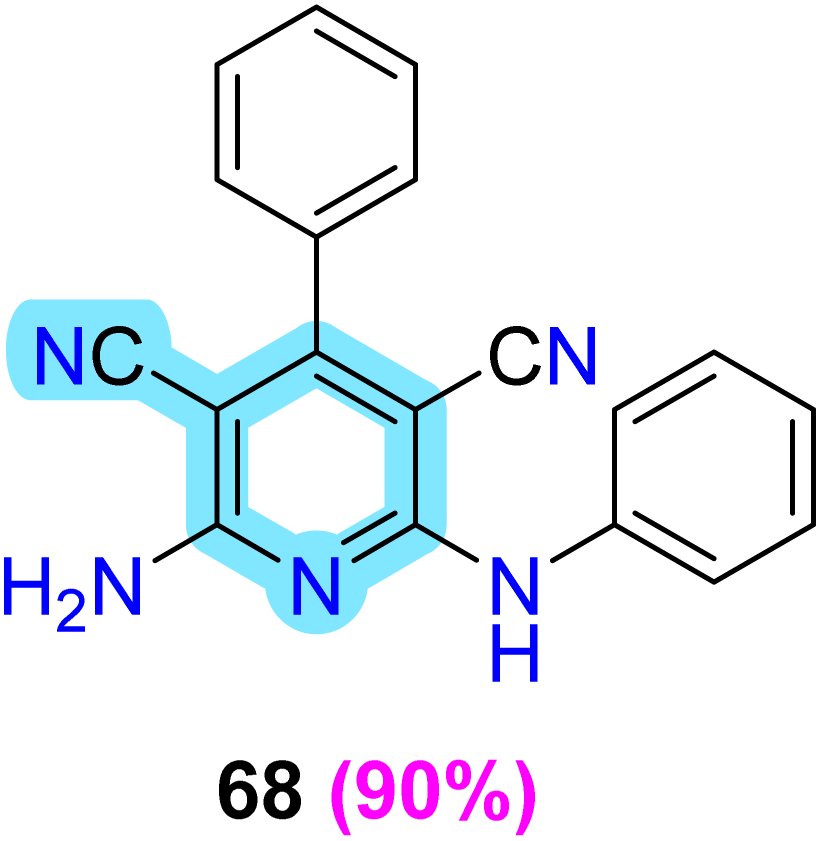	251	Enzymes	Docking study	Cell lines	IC_50_ [µM]		81
				68		68	5-FU	
			CDK2	68 displayed the best docking score of −9.5	HepG2	0.242	0.233	
					MDA-MB-231	81.3	0.49	
					Hela	74.1	100	
					PC3	0.56	7.49

**Table:** 

Scheme	Structure	m.p. (°C)	Kinase inhibition activity			Anticancer activity			Ref.
13	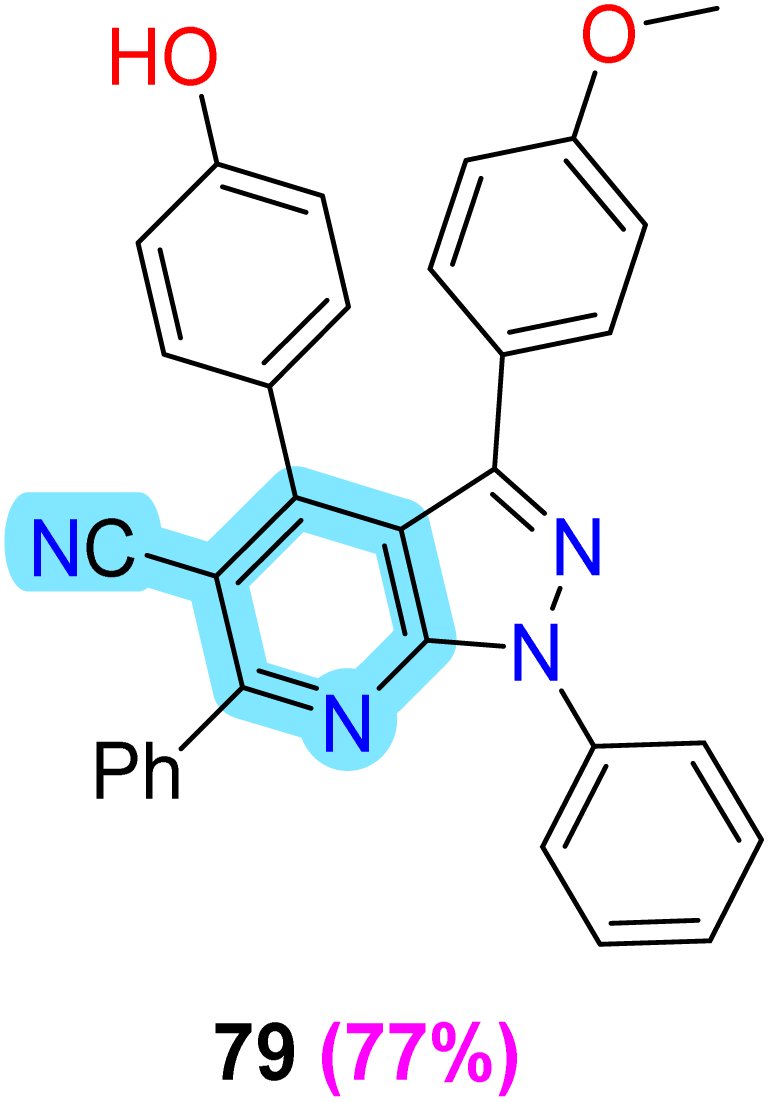	295–297	Enzymes	IC_50_ [µM]		Cell lines	IC_50_ [µM]		82
				79	Ribociclib		79	DOX	
			CDK2	0.460 ± 0.024	0.068 ± 0.004	Hela	12.65 ± 1.53	2.35 ± 0.08	
						MCF7	4.66 ± 0.38	4.57 ± 0.28	
			CDK9	0.801 ± 0.041	0.050 ± 0.003	HCT116	1.98 ± 0.17	2.11 ± 0.20

**Table:** 

Scheme	Structure	m.p. (°C)	Kinase inhibition activity		Anticancer activity			Ref.
14	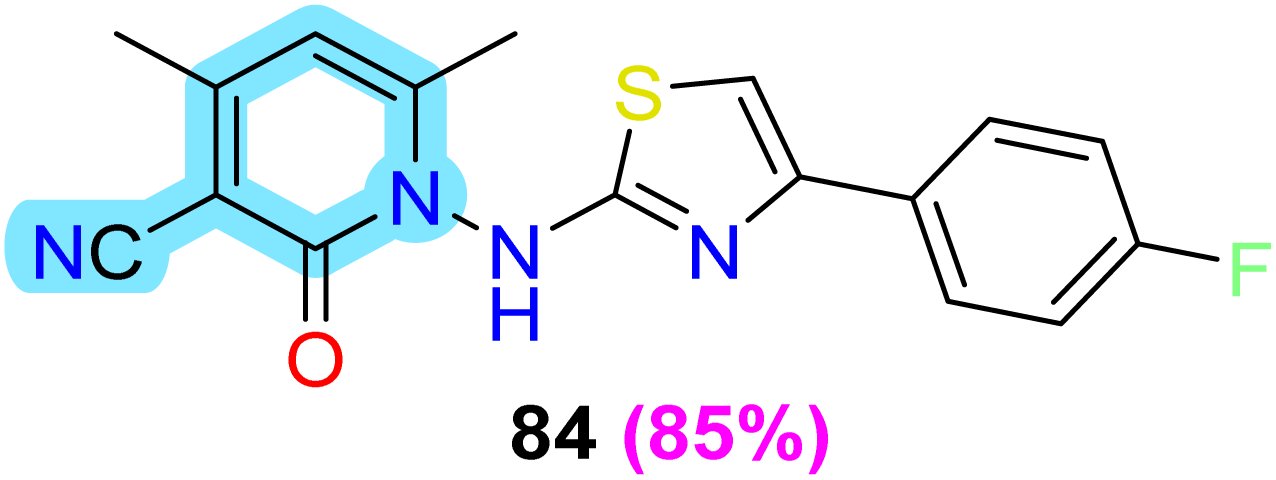	232–233	Enzymes	Docking study	Cell lines	IC_50_ [µM]		83
				84		84	DOX	
			CDK2	84 displayed the best docking score of −8.2	MCF-7	10.9 ± 0.5	2.1 ± 0.5	
					HePG2	6.98 ± 0.3	1.6 ± 0.4	
					HCT116	6.3 ± 0.2	1.9 ± 0.3

**Table:** 

Scheme	Structure	m.p. (°C)	Kinase inhibition activity		Anticancer activity			Ref.
15	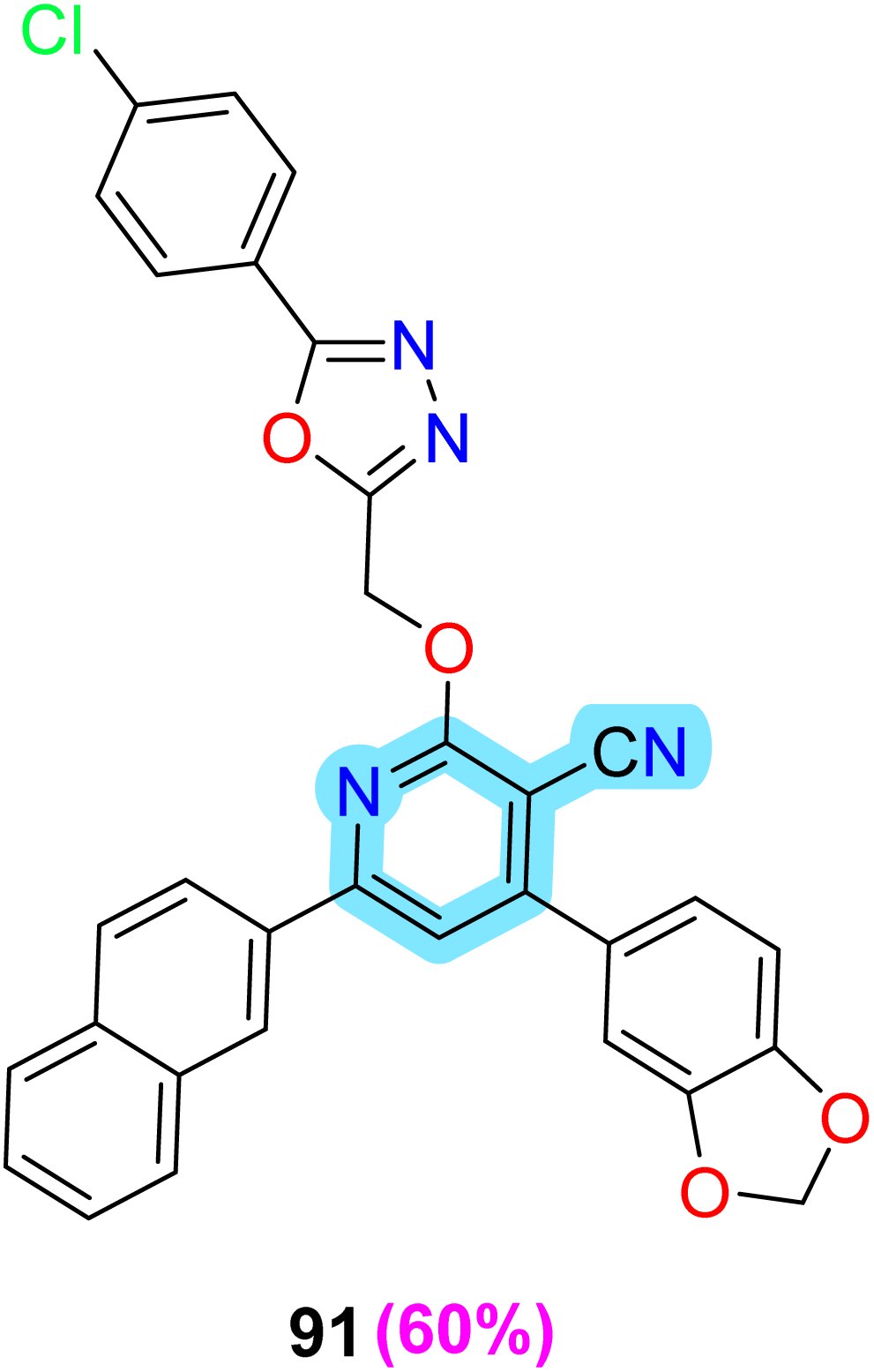	140–142	Enzymes	Docking study	Cell lines	IC_50_ [µg ml^−1^]		84
				91		91	5-FU	
			CDK2	91 displayed the best docking score of −21.8	MCF-7	26.95	Over 100	
					MDA-MB-231	29.08	Over 100

**Table:** 

Scheme	Structure	m.p. (°C)	Kinase inhibition activity			Anticancer activity					Ref.
16	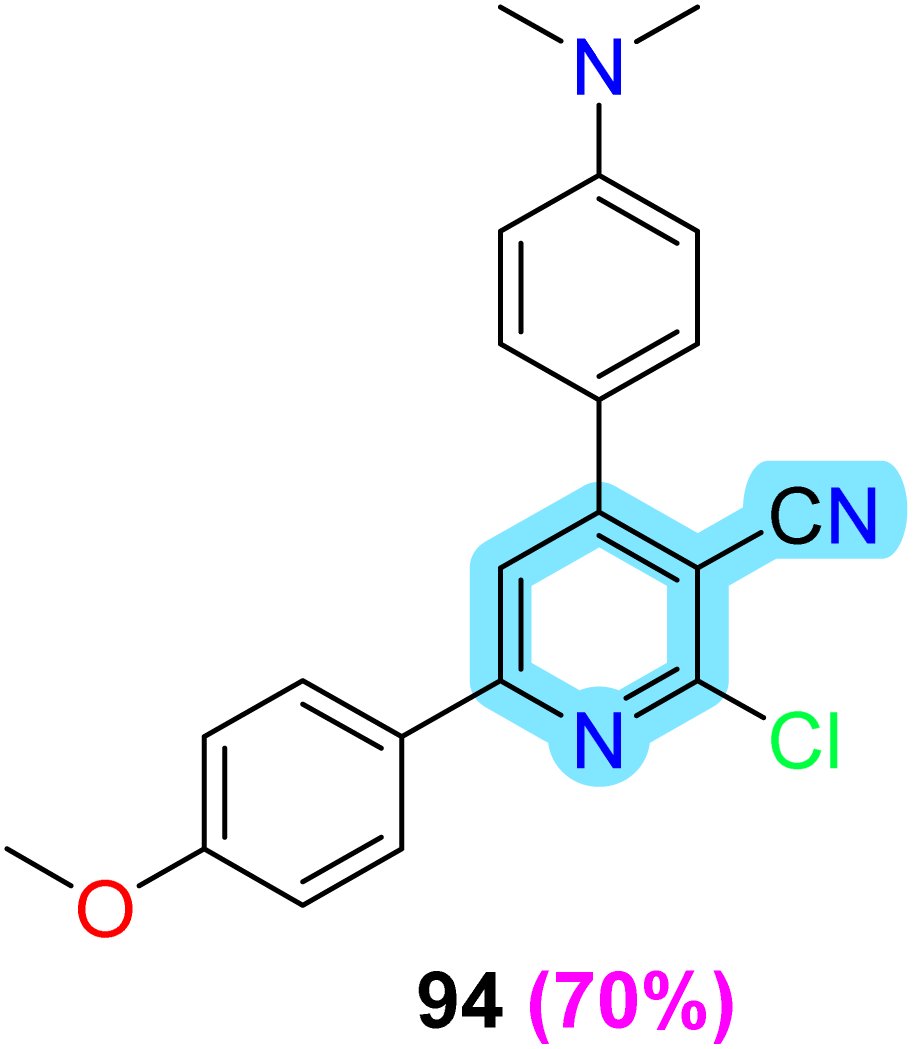	228–230	Enzymes	IC_50_ [µM]		Cell lines	IC_50_ [µM]				27
				94	Staurosporine		94	Taxol	Staurosporine	Epirubicin	
			PIM-1 kinase	0.28 ± 0.01	0.46 ± 0.02	HepG2	0.35 ± 0.02	22.35 ± 1.26	*ca.* 0.04	*ca.* 2.90	
						MCF-7	18.24 ± 1.02	6.81 ± 0.38	*ca.* 0.5	*ca.* 55.3

**Table:** 

Scheme	Structure	m.p. (°C)	Kinase inhibition activity				Anticancer activity			Ref.
17	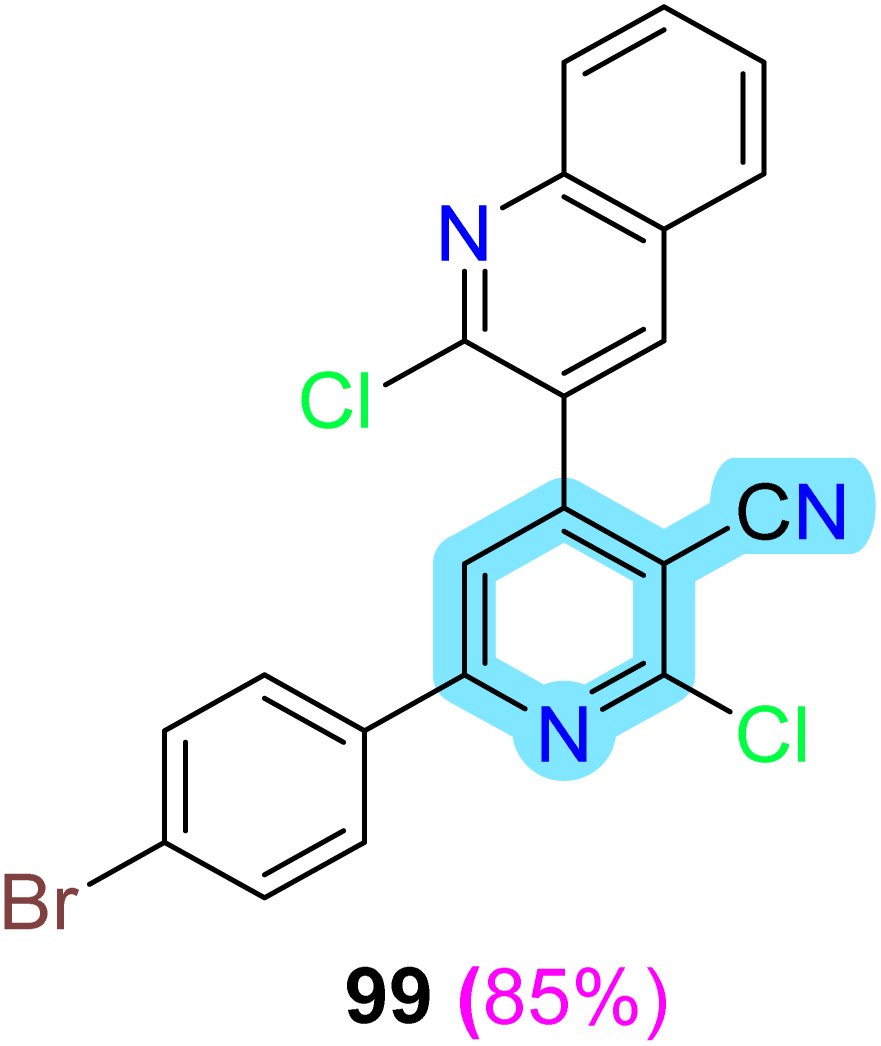	245–247	Enzymes	IC_50_ [µM]			Cell lines	IC_50_ [µM]		65
				99	PIM-1 kinase inhibitor A (VRV)	78.65 ± 2.1		99	5-FU	
			PIM-1 kinase	0.46 ± 0.02	0.93 ± 0.05	0.56 ± 0.03	HepG2	6.95 ± 0.34	9.42 ± 0.46	
							MCF-7	8.50 ± 0.42	7.75 ± 0.37	
							PC-3	14.08 ± 0.70	10.34 ± 0.50	
							HCT-116	8.35 ± 0.42	8.01 ± 0.39

**Table:** 

Scheme	Structure	m.p. (°C)	Kinase inhibition activity			Anticancer activity			Ref.
18	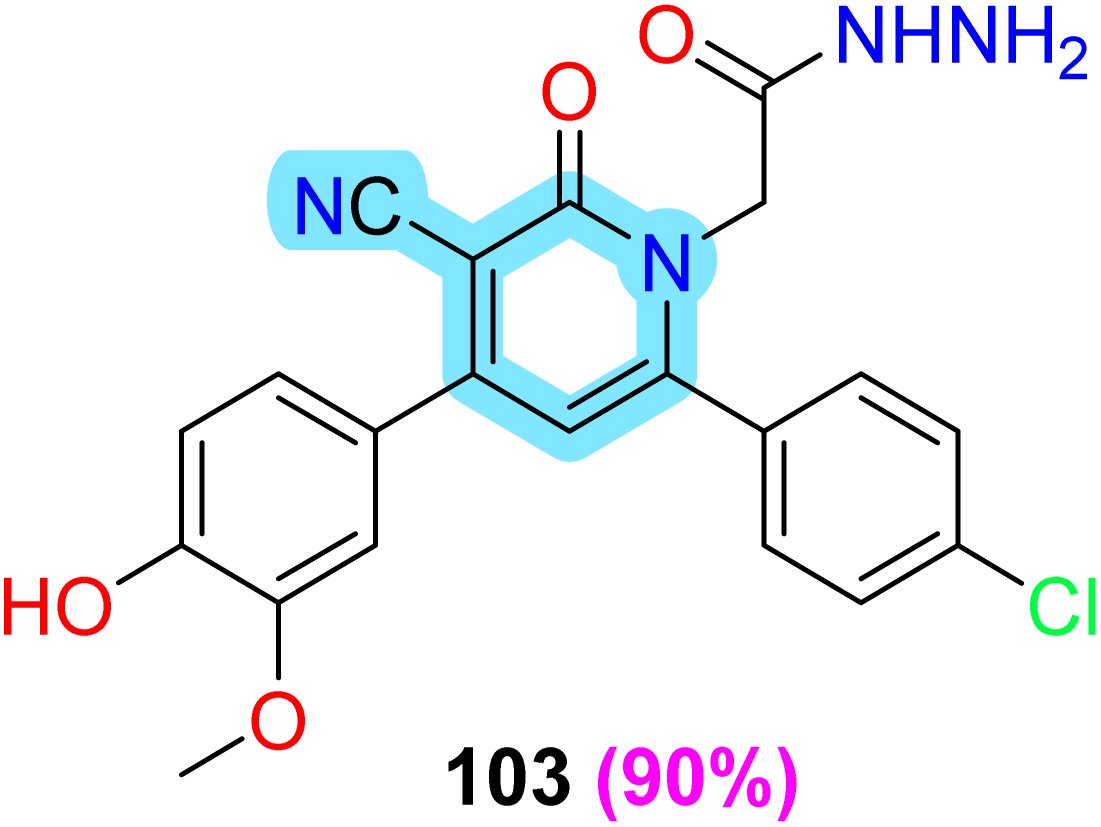	226–228	Enzymes	IC_50_ [nM]		Cell lines	IC_50_ [µM]		86
				103	Staurosporine		103	Staurosporine	
			PIM-1 kinase	8.4 ± 0.2	16.7 ± 0.32	MCF-7	0.44 ± 0.01	6.76	
						Hep G2	0.28 ± 0.01	—

**Table:** 

Scheme	Structure	m.p. (°C)	Kinase inhibition activity			Anticancer activity			Ref.
19	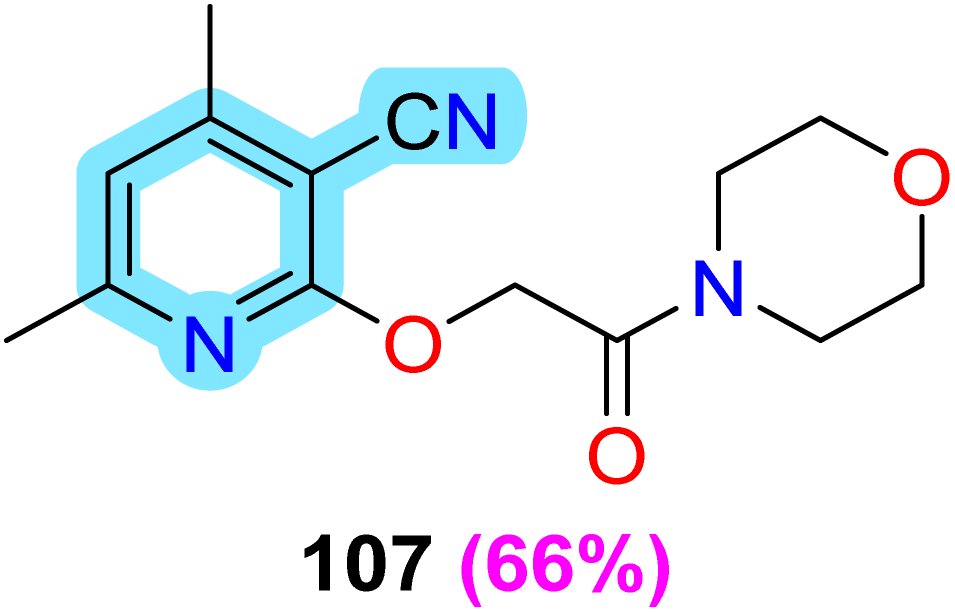	131	Enzymes	IC_50_ [nM]		Cell lines	IC_50_ [µM]		87
				107	Staurosporine		107	DOX	
			PIM-1 kinase	14.3 ± 0.6	16.7	MCF-7	0.51 ± 0.02	2.14 ± 0.1	
						HepG2	5.27 ± 0.2	2.48 ± 0.1	
						MCF-10A	52.85 ± 2.1	15.75 ± 0.63

**Table:** 

Scheme	Structure	m.p. (°C)	Kinase inhibition activity			Anticancer activity			Ref.
20	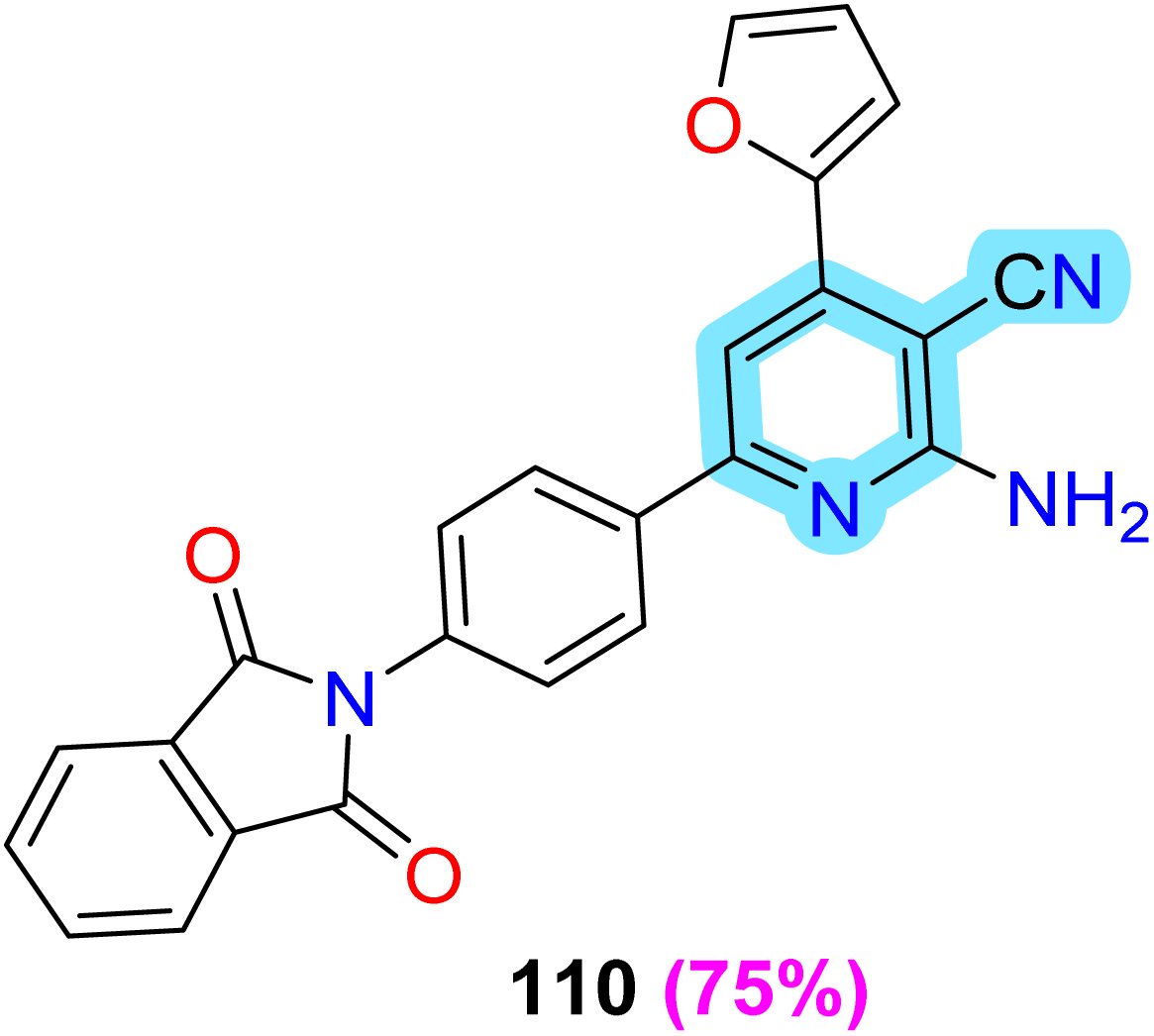	330–332	Enzymes	IC_50_ (µM)		Cell lines	IC_50_ [µM]		30
				110	Colchicine		110	5-FU	
			PIM-1 kinase	0.63 ± 0.02	3.98 ± 0.15	PC-3	4.82 ± 0.36	7.53 ± 0.57	
				Kinase inhibition (%) at 10 µM		HepG2	5.19 ± 0.43	8.15 ± 0.61	
				76.43	64.15	MCF-7	8.17 ± 0.83	7.76 ± 0.46

**Table:** 

Scheme	Structure	m.p. (°C)	Kinase inhibition activity			Anticancer activity			Ref.
21	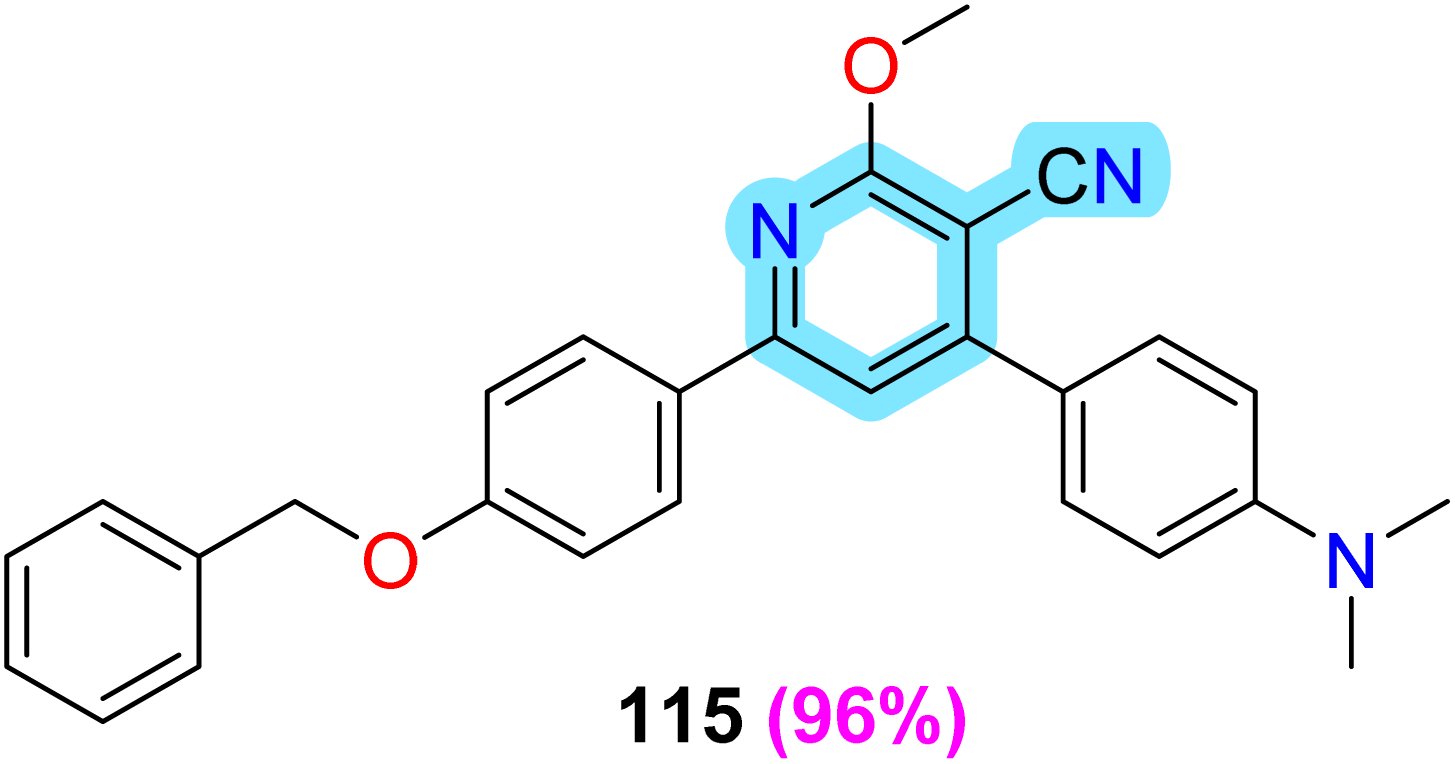	157–159	Enzymes	IC_50_ (µM)		Cell lines	IC_50_ [µM]		88
				115	Staurosporine		115	Staurosporine	
			pim-1 kinase	0.13 ± 0.008	0.294 ± 0.017	PC-3	0.99 ± 0.05	2.08 ± 0.1	
						DU-145	8.03 ± 0.38	13.4 ± 0.64	
						WI-38	57.30 ± 0.61	23.63 ± 0.94

**Table:** 

Scheme	Structure	m.p. (°C)	Kinase inhibition activity			Anticancer activity			Ref.
22	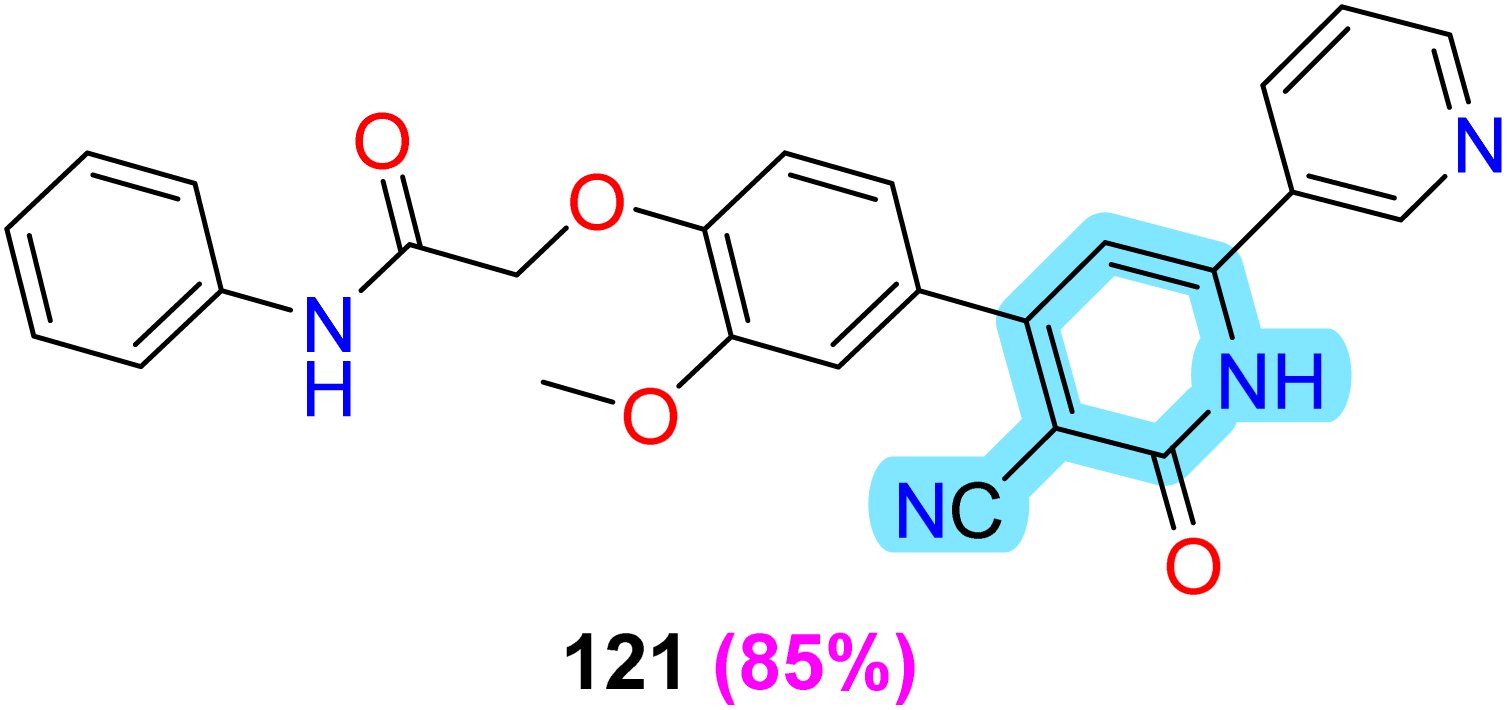	295–297	Enzymes	IC_50_ (µM)		Cell lines	IC_50_ [µM]		89
				121	Staurosporine		121	DOX	
			PIM-1 kinase	0.281 ± 0.012	0.223 ± 0.01	MCF-7	1.89 ± 0.08	11.49 ± 0.47

**Table:** 

Scheme	Structure	m.p. (°C)	Kinase inhibition activity		Anticancer activity			Ref.
23	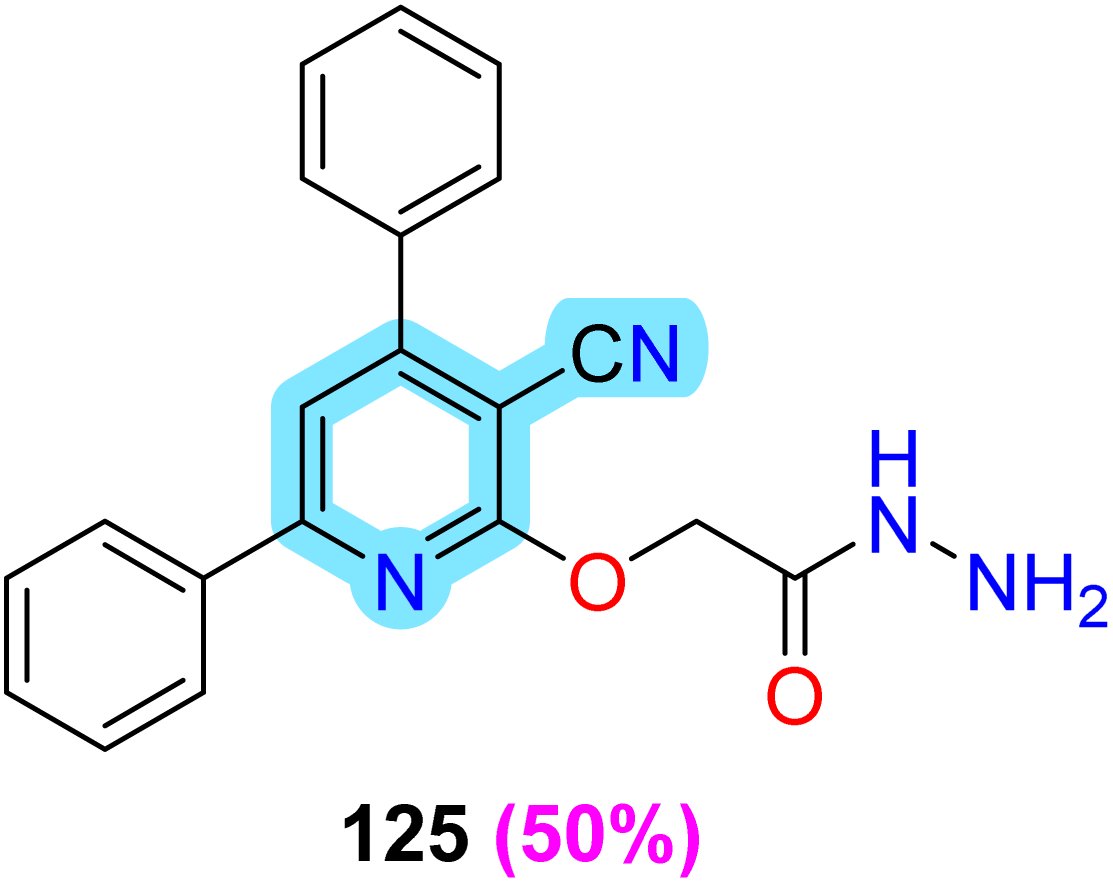	204	Enzymes	Inhibition the PIM-1 kinase gene expression	Cell lines	IC_50_ [µM]		90
				125		125	5-FU	
			PIM-1 kinase	0.45 ± 0.04	HepG2	7.26 ± 0.34	6.98	
					THLE2	≥50	ND

**Table:** 

Scheme	Structure	m.p. (°C)	Kinase inhibition activity			Anticancer activity			Ref.
24	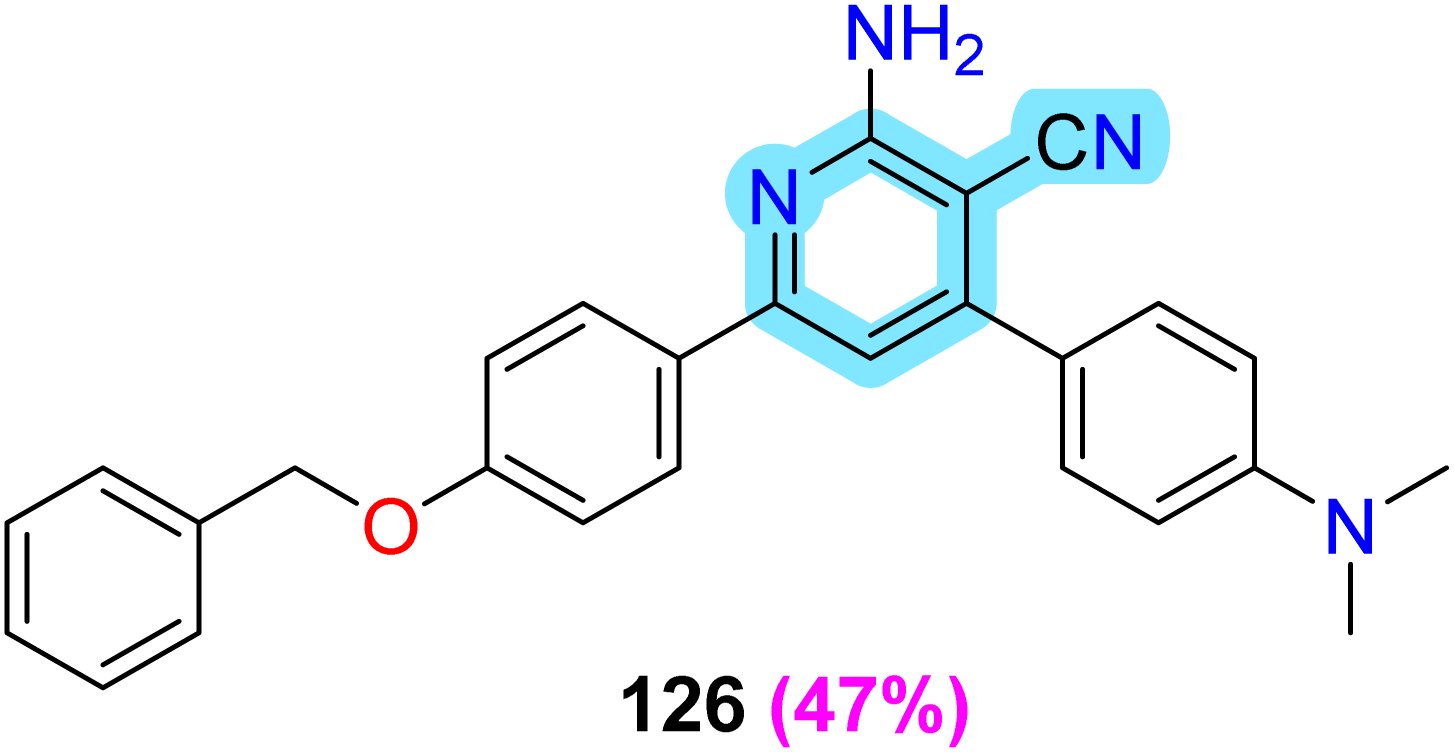	145–147	Enzymes	IC_50_ (µM)		Cell lines	IC_50_ [µM]		26
							126	5-FU	
				126	Colchicine	PC-3	2.04 ± 0.21	7.53 ± 0.57	
			PIM-1 kinase	0.47	3.98	HepG2	3.73 ± 0.18	8.15 ± 0.61	
				Kinase inhibition (%) at 10 µM		MCF-7	8.66 ± 0.73	7.76 ± 0.46	
				80.22	64.15

**Table:** 

Scheme	Structure	m.p. (°C)	Kinase inhibition activity			Anticancer activity			Ref.
25	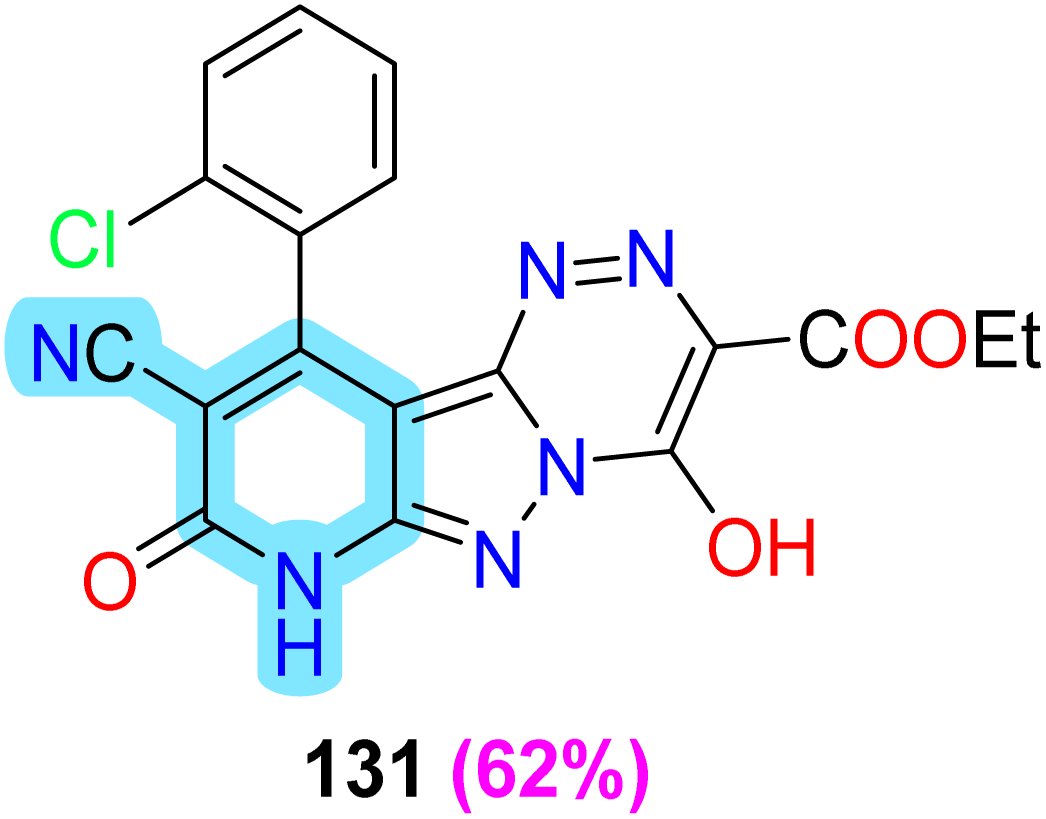	278–280	Enzymes	IC_50_ (nM)		Cell lines	IC_50_ [µM]		91
							131	5-FU	
			PIM-1 kinase	131	Staurosporin	MCF-7	5.61 ± 0.15	5.8 ± 0.34	
				26 ± 0.98	17	MDA-MB-231	38.7 ± 1.03	15.74 ± 0.65

**Table:** 

Scheme	Structure	m.p. (°C)	Kinase inhibition activity				Anticancer activity				Ref.
26	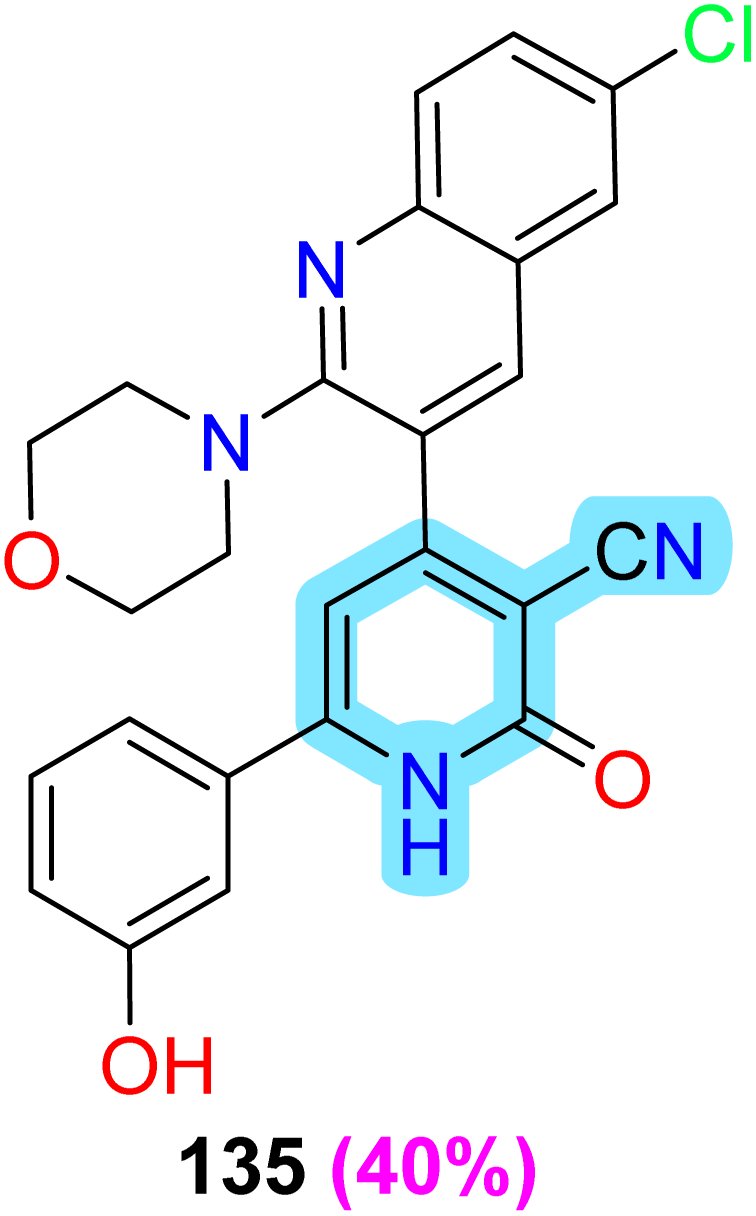	>300	Enzymes	IC_50_ [µM]			Cell lines	IC_50_ [µM]			92
				135	137	Quercetin		135	137	DOX	
		>300	PIM-1 kinase	1.31	1.27	1.17	HepG-2	0.00528 ± 0.0001	0.00238 ± 0.0001	0.0504	
	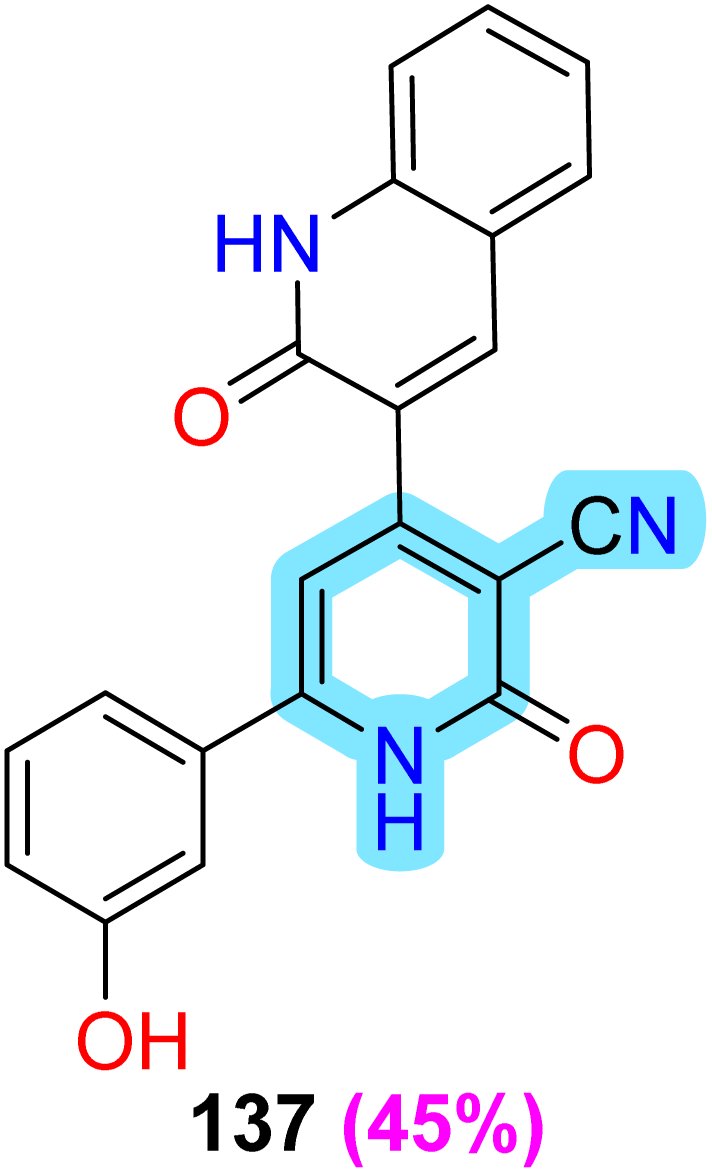						Caco-2	0.0453 ± 0.001	0.0228 ± 0.001	—	
			PIM-2 kinase	0.67	—	0.61	PC-3	0.0261 ± 0.004	0.0200 ± 0.003	—	
							NFS-60	0.00932 ± 0.004	0.009 ± 0.0001	0.0332

**Table:** 

Scheme	Structure	m.p. (°C)	Kinase inhibition activity				Anticancer activity			Ref.
27	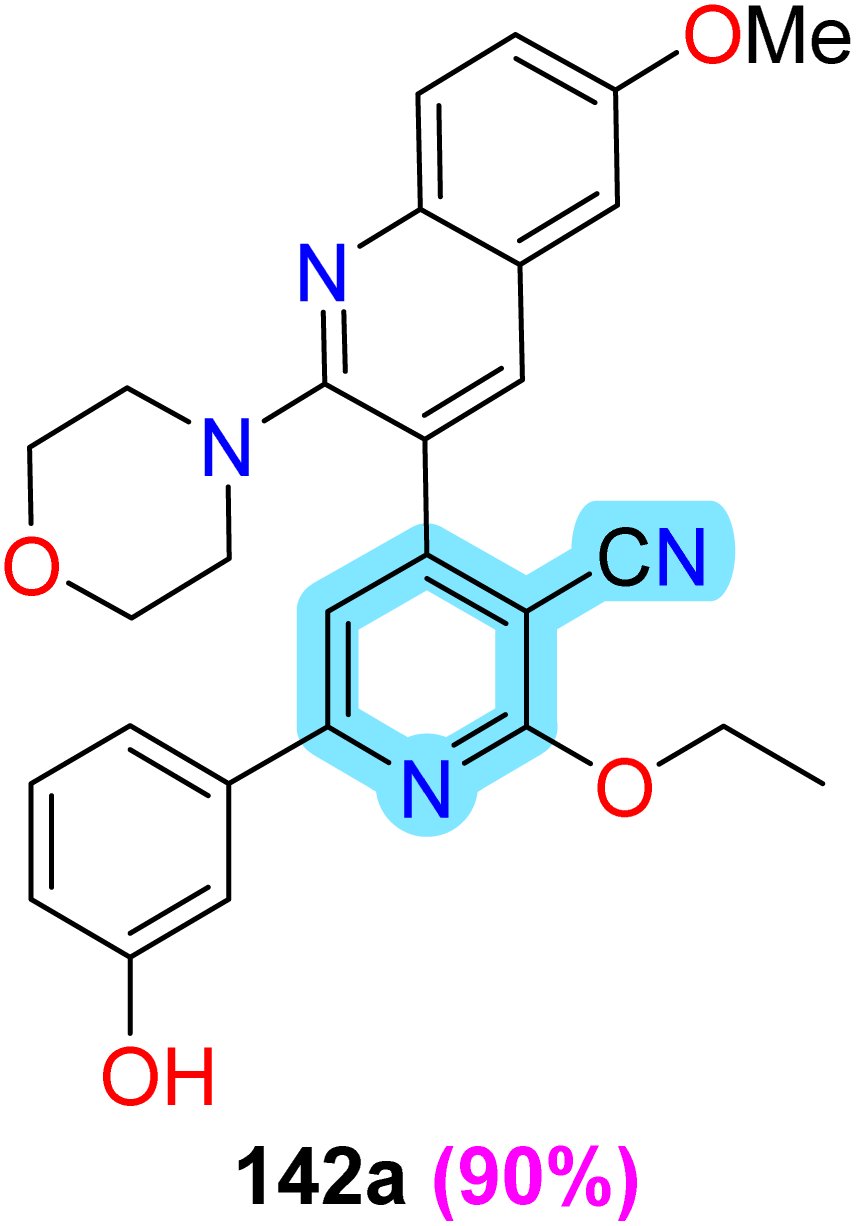	280–281	Enzymes	IC_50_ [µM]			Cell lines	IC_50_ [µM]		93
				142a	142b	Quercetin		142a		
			PIM-1 kinase	0.110 ± 0.0027	0.095 ± 0.0023	1.17 ± 0.0294	HepG-2	0.0132 ± 0.002	—	
	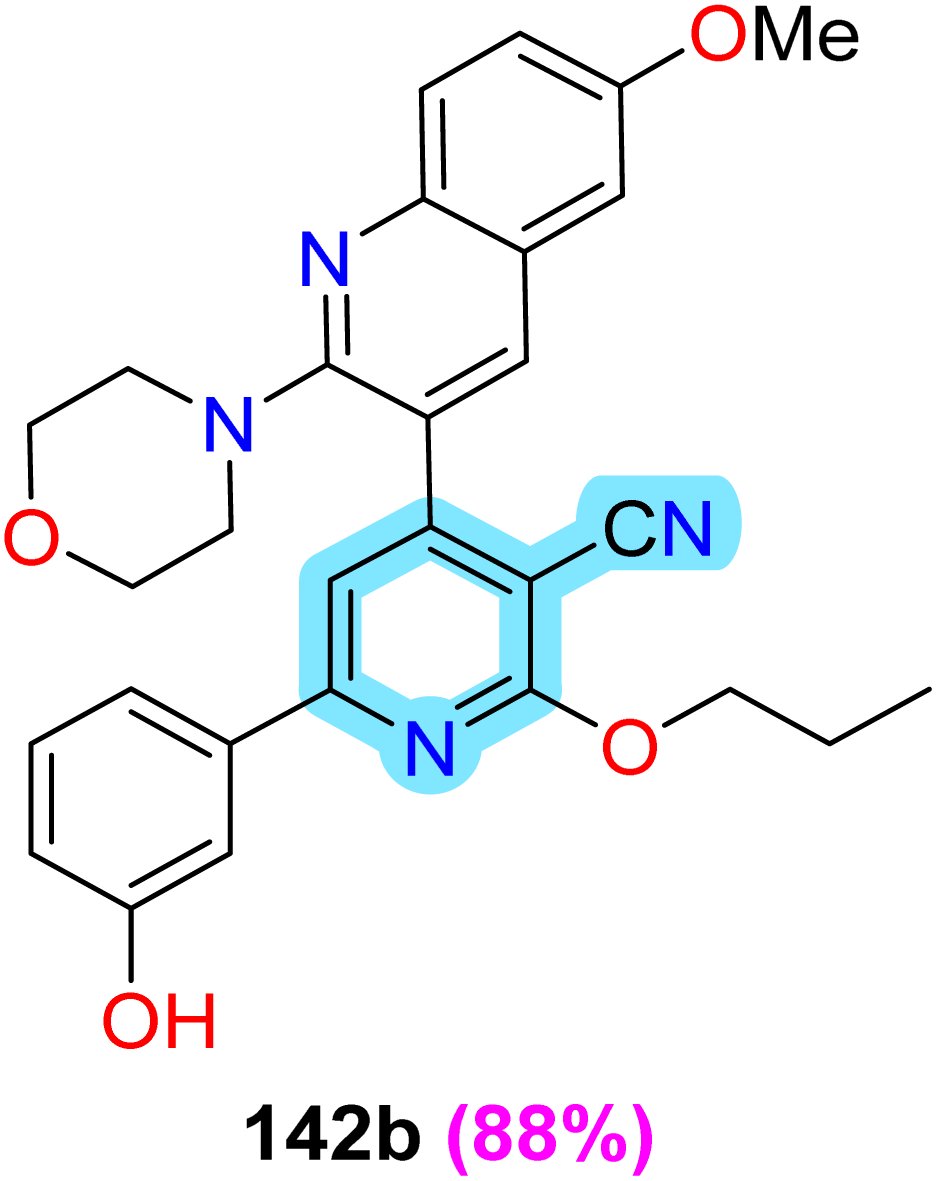	200–202					Caco-2	0.007 ± 0.001	—	
							PC-3	0.005 ± 0.0002	—	
							NFS-60	0.006 ± 0.0002	—

**Table:** 

Scheme	Structure	m.p. (°C)	Kinase inhibition activity			Anticancer activity			Ref.
28	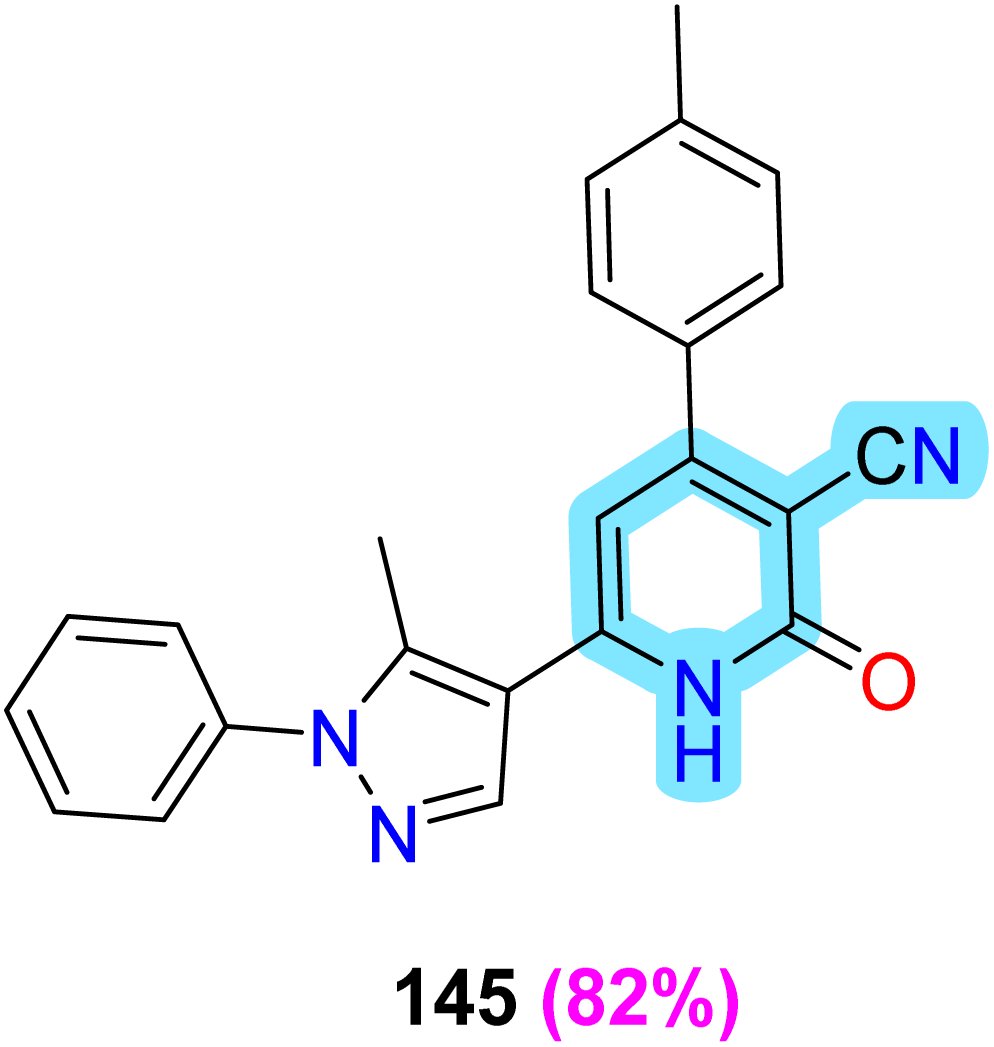	>300	Enzymes	IC_50_ [nM]		Cell lines	IC_50_ [µM]		25
				145	Staurosporine		145		
			PIM-1 kinase	110 ± 24	276 ± 12	PC-3	5.24 ± 0.33	—	
			c-Met	145	Tivantinib	HCT116	2.79 ± 0.21	—	
				151 ± 14	146 ± 5	MDA-MB-231	3.71 ± 0.32	—

**Table:** 

Scheme	Structure	m.p. (°C)	Kinase inhibition activity					Anticancer activity					Ref.
29	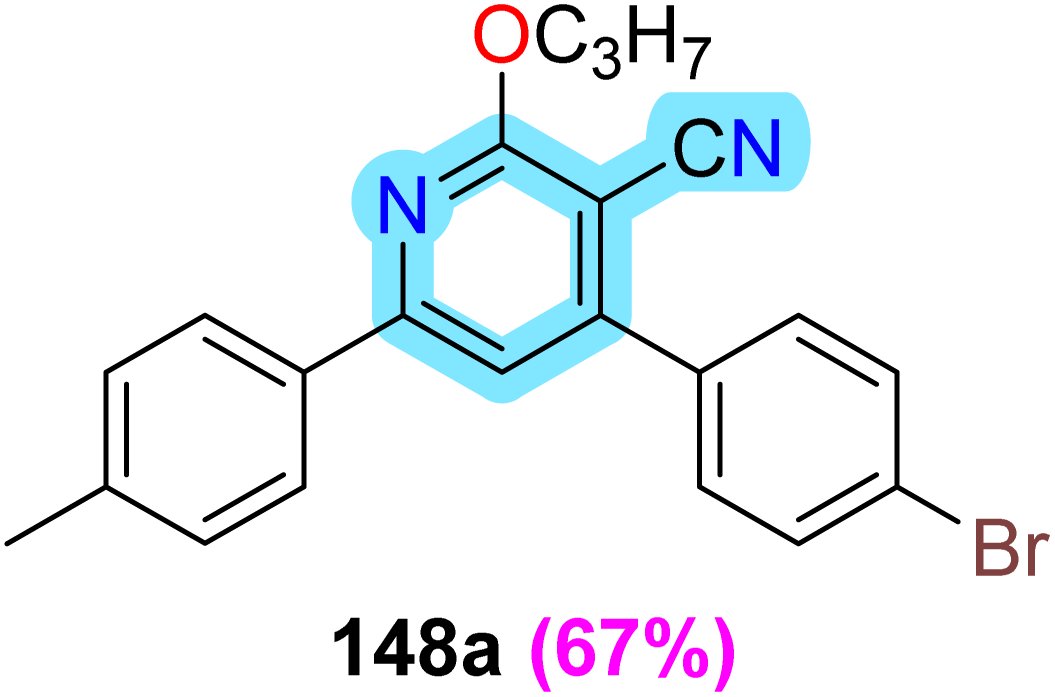	170–172	Enzymes	IC_50_ (µM)				Cell lines	IC_50_ [µM]				61
				148a	148b	SOR	MTX		148a	148b			
		200–202	EGFR	0.248 ± 0.011	0.138 ± 0.006	0.110 ± 0.006	—	Hep G2	9.14 ± 0.8	6.39 ± 0.5	—	—	
								HeLa	7.33 ± 0.6	4.92 ± 0.3	—	—	
	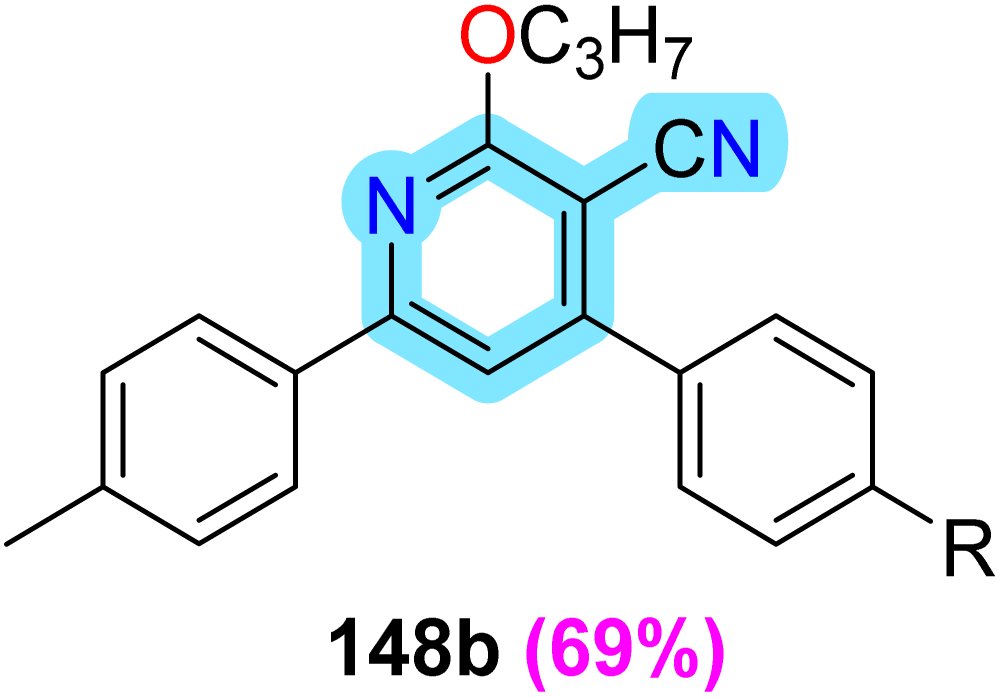		HER2	0.156 ± 0.006	0.092 ± 0.004	0.131 ± 0.008	—	HEp-2	14.18 ± 1.1	8.97 ± 0.7	—	—	
								HCT 116	24.87 ± 1.8	10.84 ± 0.9	—	—	
			DHFR	0.138 ± 0.007	0.193 ± 0.01	—	0.061 ± 0.003	PC-3	20.07 ± 1.5	12.39 ± 1.0	—	—	
								MCF-7	6.16 ± 0.4	3.84 ± 0.2	—	—

**Table:** 

Scheme	Structure	m.p. (°C)	Kinase inhibition activity				Anticancer activity			Ref.
30	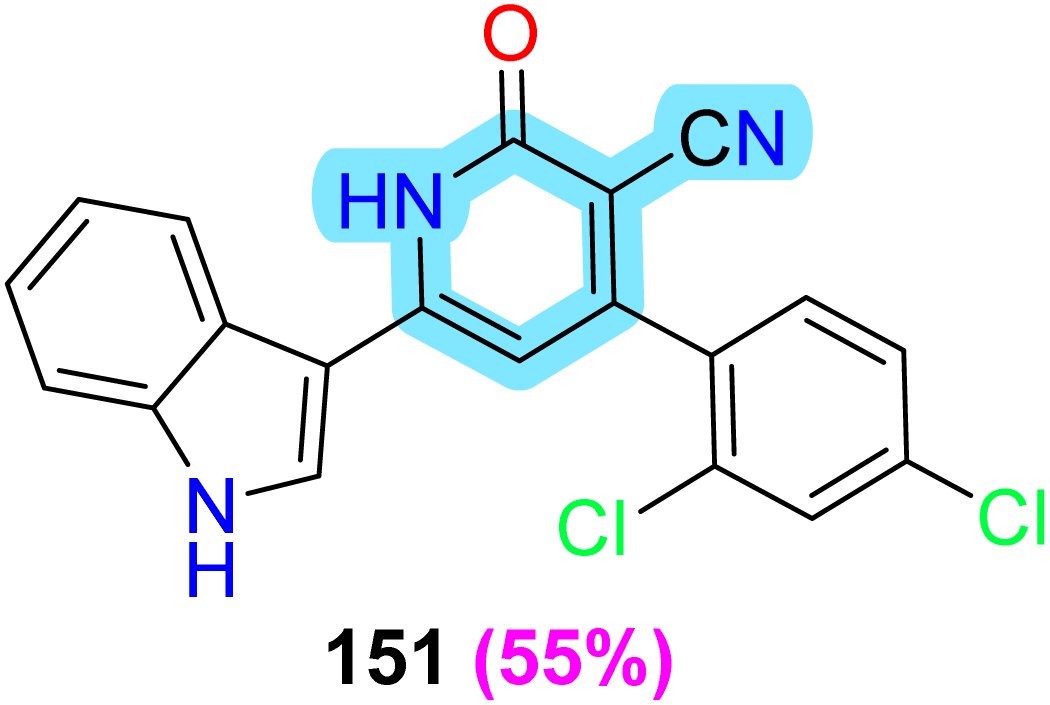	371–373	Enzymes	IC_50_ (nM)			Cell lines			95
				151	Staurosporine	Abemaciclib		151		
			EGFR	69 ± 3	135 ± 6	—	HOP-92	00.75 ± 00.09	—	
							SR	02.77 ± 00.19	—	
			c-MET	205 ± 12	330 ± 20	—	RXF 393	03.12 ± 00.33	—	
			CDK4	95 ± 5	—	29 ± 1	HCT-116	02.53 ± 00.26	—	
			CDK6	184 ± 9	—	74 ± 4

**Table:** 

Scheme	Structure	m.p. (°C)	Kinase inhibition activity			Anticancer activity			Ref.
31	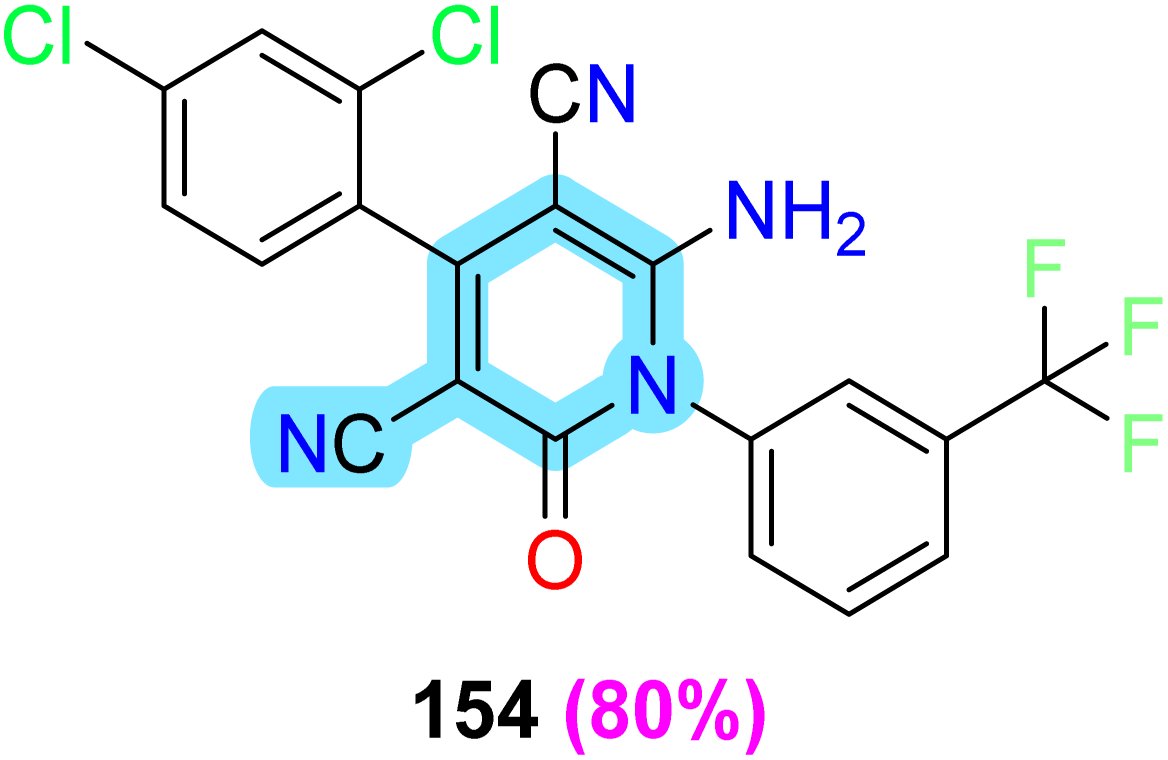	239–241	Enzymes	IC_50_ (µM)		Cell lines	IC_50_ (µM)		43
				154	Lapatinib		154	—	
			VEGFR-2	0.124 ± 0.011	0.182 ± 0.010	MCF-7	1.39 ± 0.08	—	
			HER-2	0.077 ± 0.003	0.131 ± 0.012	HepG2	10.70 ± 0.58	—

**Table:** 

Scheme	Structure	m.p. (°C)	Kinase inhibition activity					Anticancer activity			Ref.
32	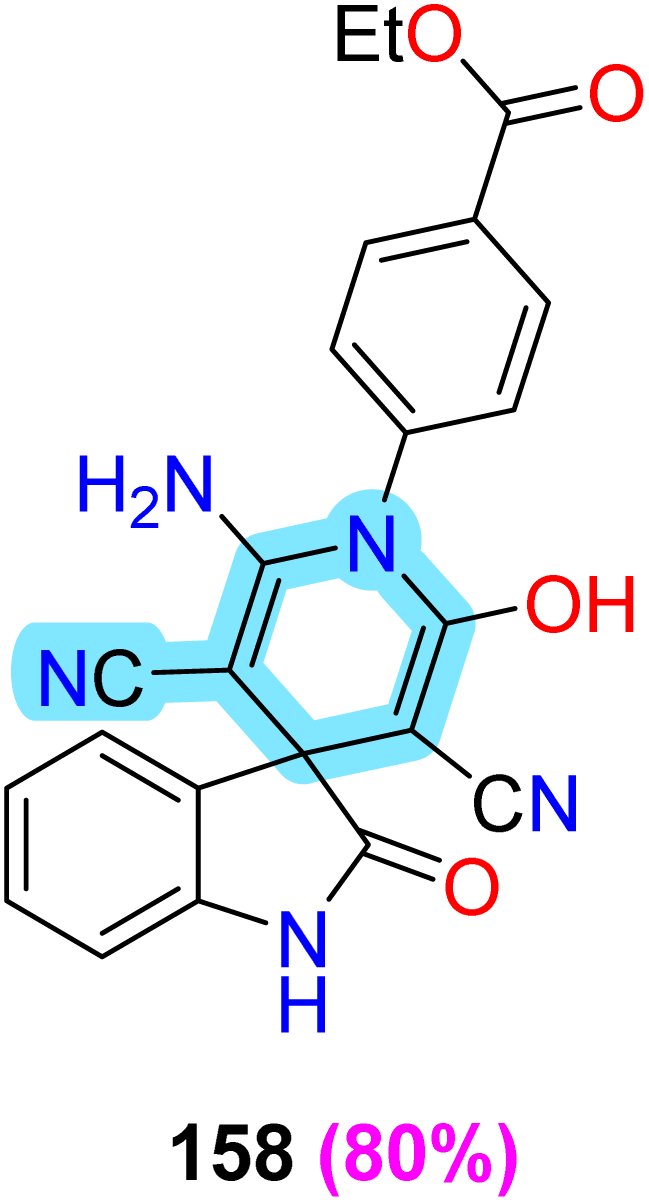	170–172	Enzymes	IC_50_ (µM)				Cell lines	IC_50_ (µM)		97
				158	DOX	Erlotinib	Sorafenib		158		
			EGFR^Wt^	0.124 ± 0.009	0.349 ± 0.016	0.033 ± 0.002	—	HepG-2	8.90 ± 0.60	—	
			VEGFR-2	0.221 ± 0.009	—	—	0.043 ± 0.002	Caco-2	7.83 ± 0.50	—

**Table:** 

Scheme	Structure	m.p. (°C)	Kinase inhibition activity			Anticancer activity			Ref.
33	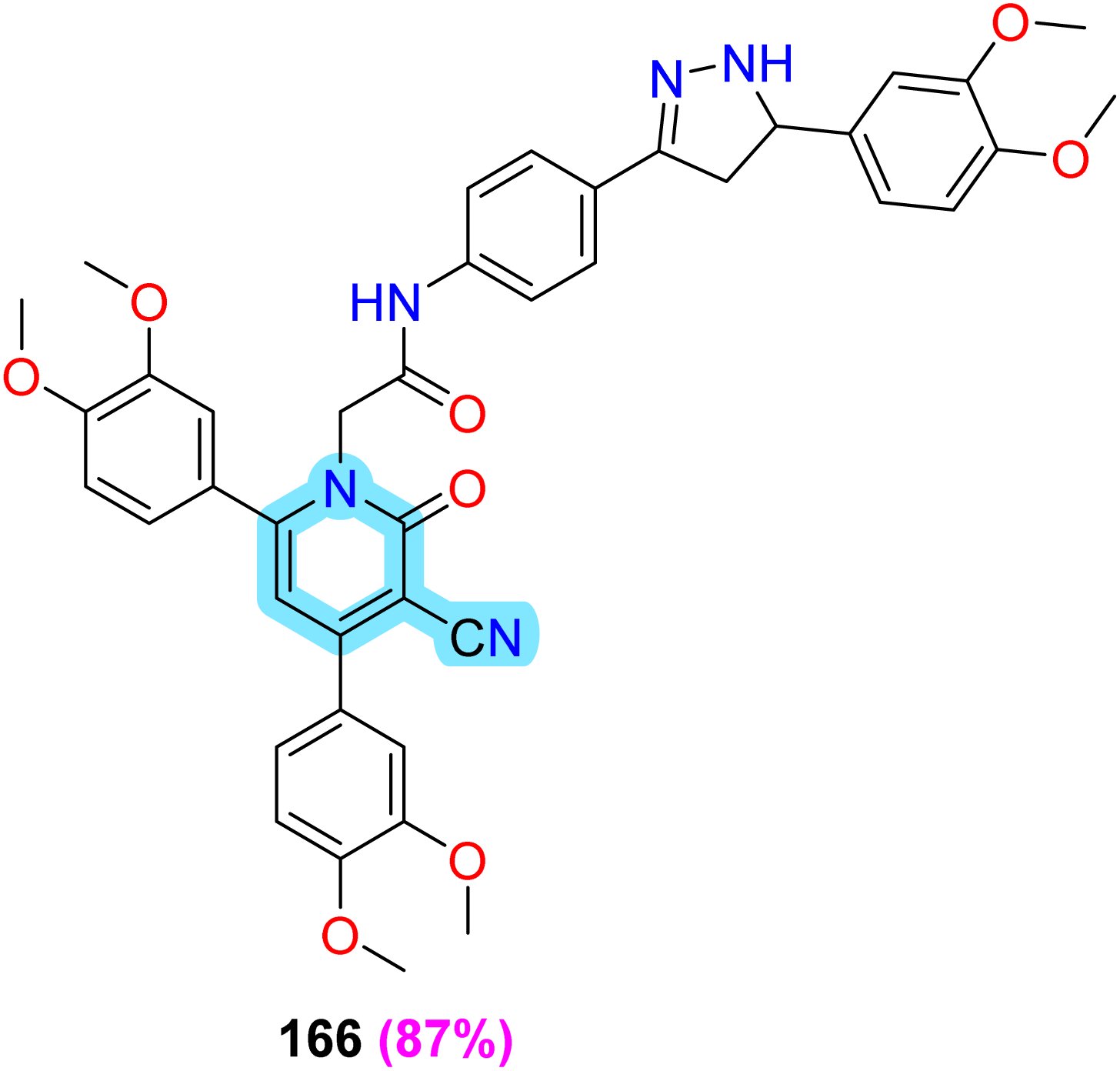	233–234	Enzymes	IC_50_ [nM]		Cell lines	IC_50_ (µM)		98
				166	Erlotinib		166		
			EGFR	68 ± 4	80 ± 5	A-549	23 ± 2	—	
						MCF-7	28 ± 2	—	
			BRAF^V600E^	65 ± 5	60 ± 5	Panc-1	24 ± 2	—	
						HT-29	24 ± 2	—

**Table:** 

Scheme	Structure	m.p. (°C)	Kinase inhibition activity			Anticancer activity			Ref.
34	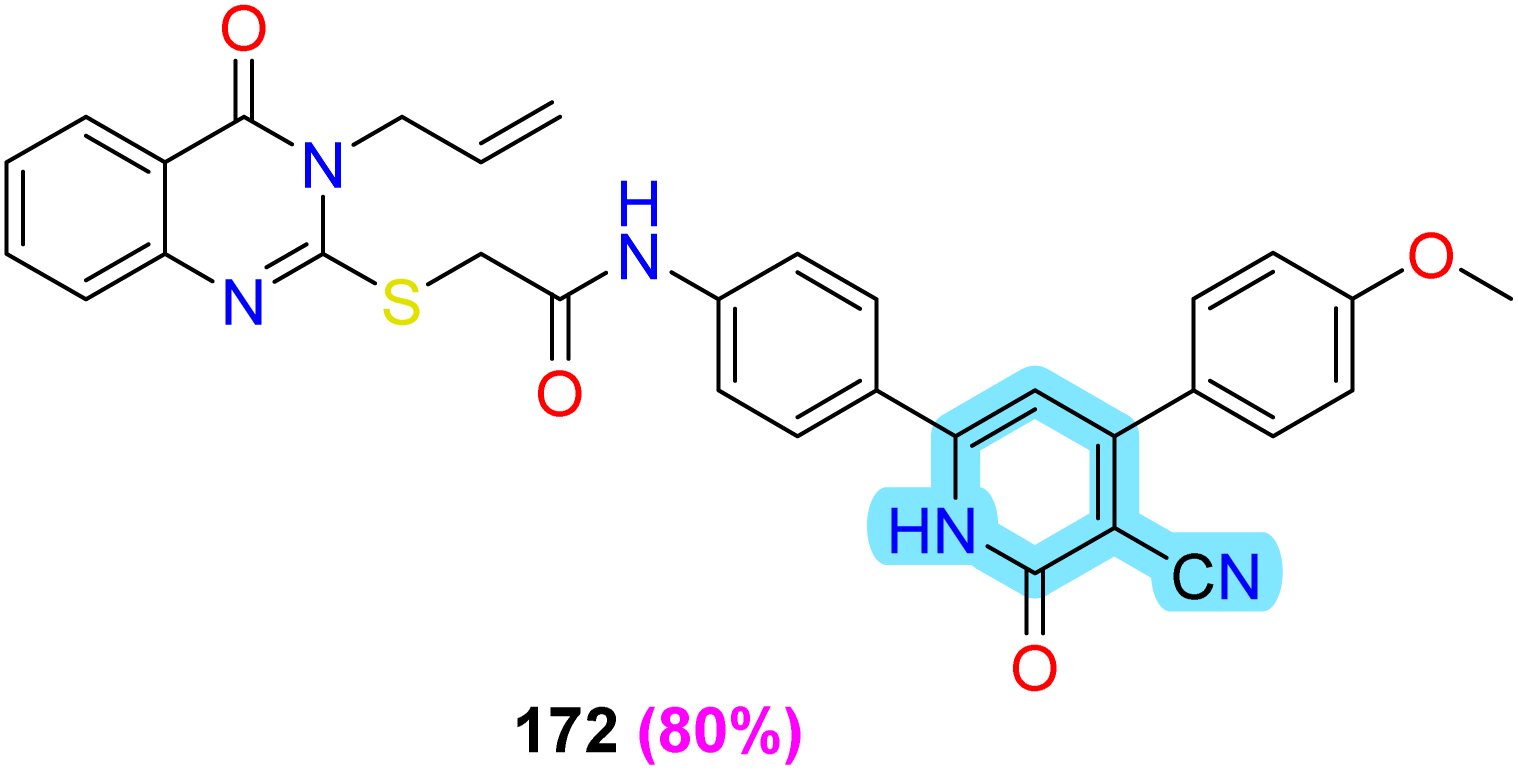	>300	Enzymes	IC_50_ [nM]		Cell lines	IC_50_ [nM]		99
				172	Erlotinib		172	—	
			EGFR	110 ± 10	80 ± 5	A-549	1.10 ± 0.10	—	
						MCF-7	1.00 ± 0.10	—	
			BRAF	140 ± 10	60 ± 5	Panc-1	1.30 ± 0.10	—	
						HT-29	1.30 ± 0.10	—

**Table:** 

Scheme	Structure	m.p. (°C)	Kinase inhibition activity		Anticancer activity			Ref.
35	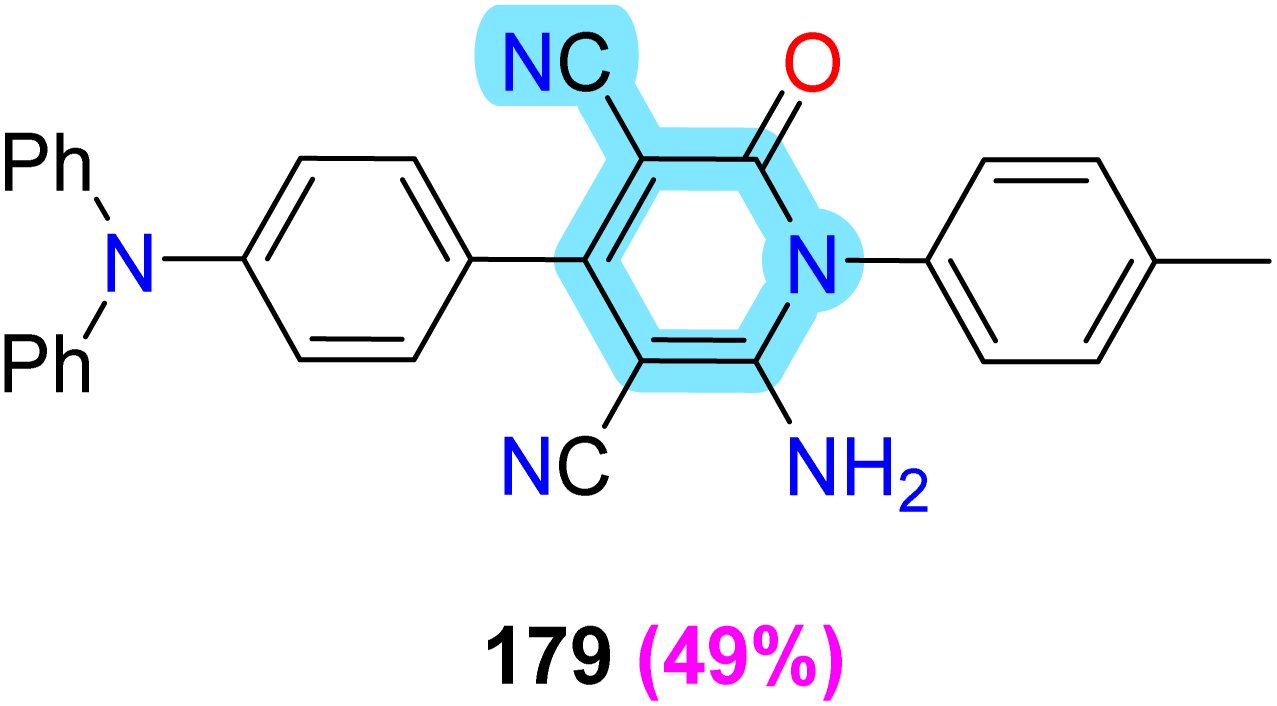	>300	Enzymes	Docking study	Cell lines			101
				179		179	—	
			—	179 displayed the best docking score of −8.3	A-549	0.00803	—	
					MDA-MB-231	0.0103	—

**Table:** 

Scheme	Structure	m.p. (°C)	Kinase inhibition activity		Anticancer activity			Ref.
36	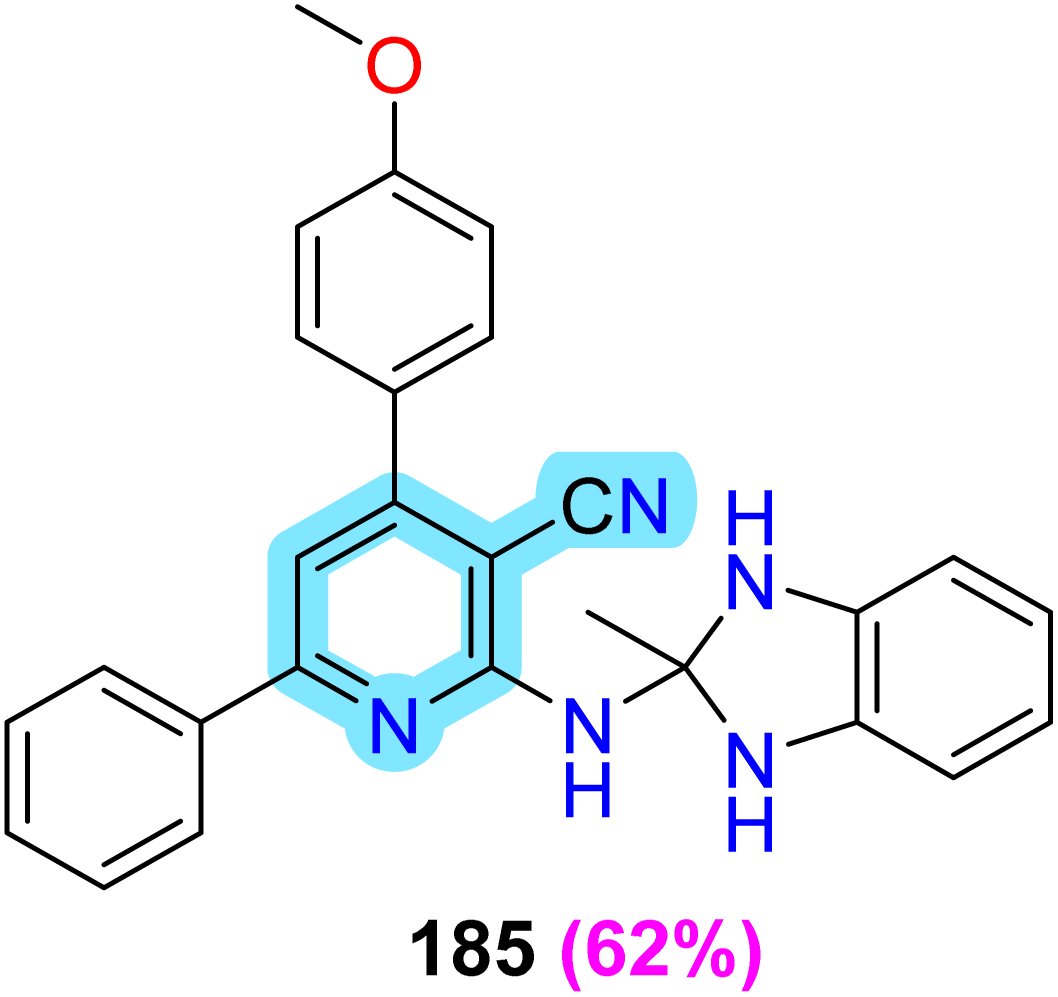	232–234	Enzymes	—	Cell lines			102
						185	—	
			—		HePG2	7.48 ± 0.6	—	
					MCF-7	5.69 ± 0.4	—

**Table:** 

Scheme	Structure	m.p. (°C)	Kinase inhibition activity		Anticancer activity			Ref.
37	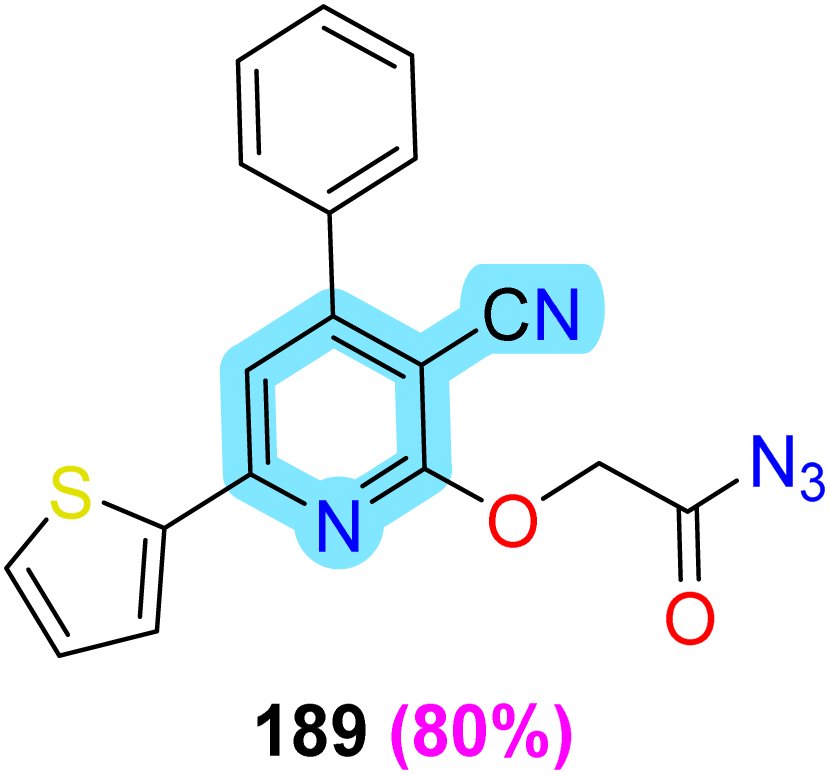	118–120	Enzymes	—	Cell lines			103
						189		
			—		HCT116	29.17 ± 1.9	—	
					HePG-2	19.85 ± 1.4	—	
					MCF7	23.9 ± 2.3	—

**Table:** 

Scheme	Structure	m.p. (°C)	Kinase inhibition activity		Anticancer activity			Ref.
38	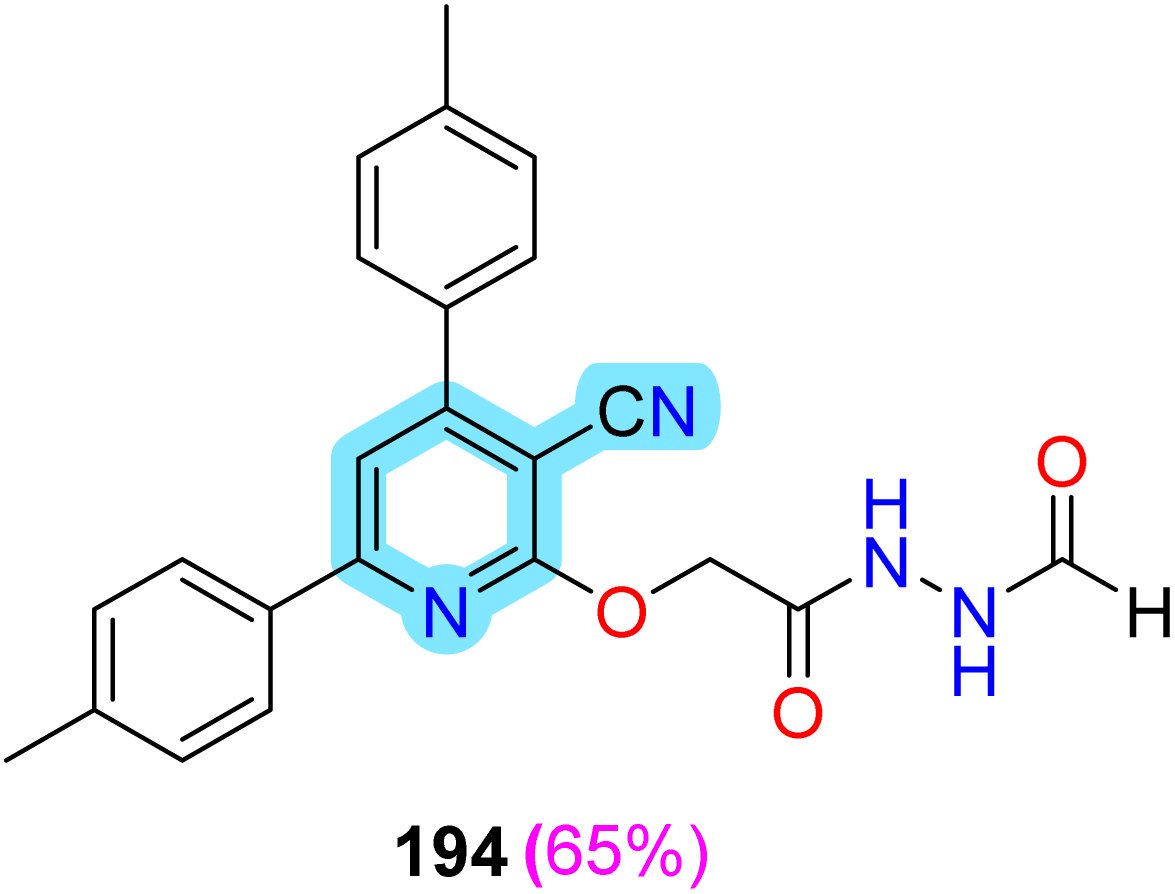	244–246	Enzymes	—	Cell lines			104
						194		
					MCF7	0.7 ± 0.3	—	
					HCT116	0.5 ± 0.04	—	
					SKOV3	0.1 ± 0.03	—

**Table:** 

Scheme	Structure	m.p. (°C)	Kinase inhibition activity		Anticancer activity			Ref
39	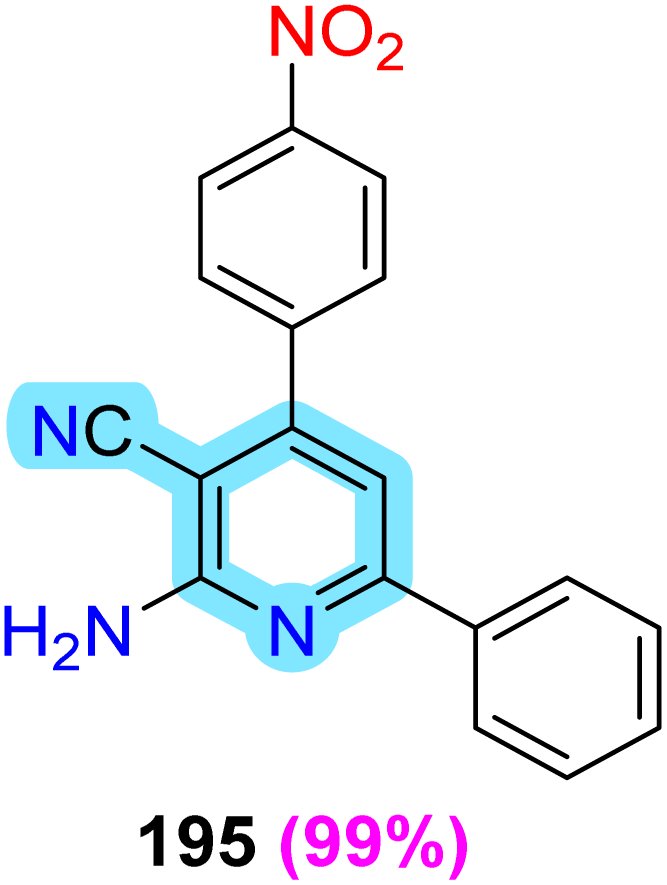	173–175	Enzymes	—	Cell lines			105
						195		
					MCF7	1.80 ± 0.11	—	
					HePG-2	7.71 ± 0.41	—	
					DU-145	36.00 ± 2.4	—

**Table:** 

Scheme	Structure	m.p. (°C)	Kinase inhibition activity		Anticancer activity						Ref
40	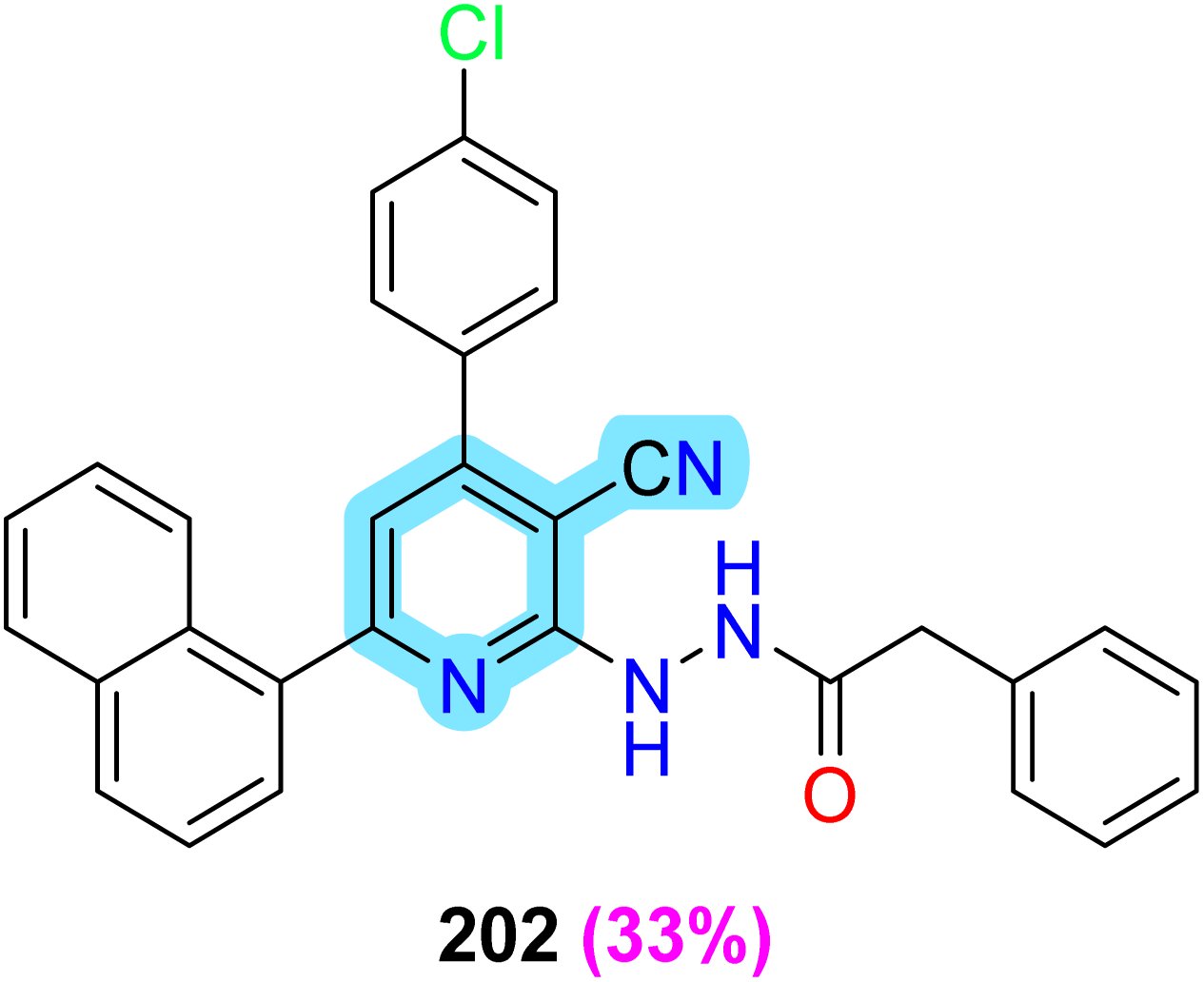	182–184	Enzymes	—	Cell lines						106
						202	DOX	—	—	—	
					U937	422 ± 25	4450 ± 50	—	—	—	
					SKMEL-28	255 ± 2		—	—	—	
					NCIH 460	25 ± 2.6		—	—	—	
					RKOP 27	16 ± 2		—	—	—	
					HeLa	127 ± 25.5			—	—

**Table:** 

Scheme	Structure	m.p. (°C)	Kinase inhibition activity		Anticancer activity			Ref
41	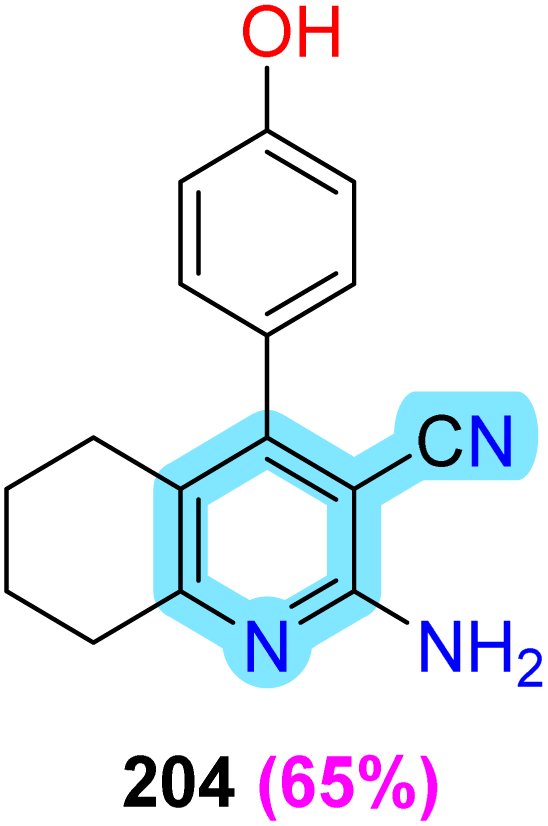	153–155	Enzymes	—	Cell lines			107
						204	—	
					HePG-2	5.86 ± 0.3	—	
					Caco-2	11.47 ± 1.1	—

**Table:** 

Scheme	Structure	m.p. (°C)	Kinase inhibition activity		Anticancer activity			Ref
42	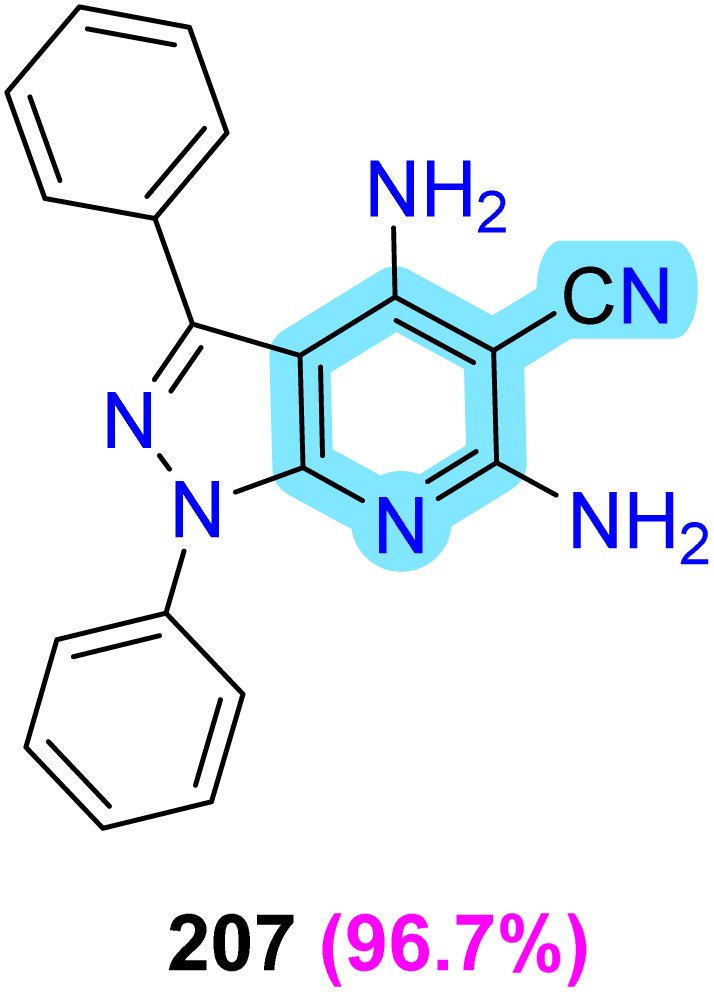	ND	Enzymes	—	Cell lines			108
						207	5-FU	
					HepG2	9.2 ± 2.8	7.86 ± 0.5	
					HCT-116	7.7 ± 1.8	5.35 ± 0.3	
					PC3	1.64 ± 0.06	0.88 ± 0.09	

Biological activity values were extracted from the respective original studies and reflect different experimental platforms, assay conditions, incubation times, enzyme constructs, cell lines, and reference compounds. Accordingly, IC_50_ and GI_50_ values from different studies should not be interpreted as direct head-to-head potency comparisons. Biochemical enzyme inhibition, cellular antiproliferative activity, and computational docking represent distinct levels of evidence and are presented separately. Concentration values were standardized to nM or µM only when scientifically valid; values originally reported in µg mL^−1^ were retained when conversion to molar concentration could not be performed reliably from the reported information. Selectivity indices were included only where both cancer-cell and non-malignant-cell IC_50_ values were available under comparable experimental conditions.

### 3.4. Translational merits and therapeutic windows of multitarget nicotinonitrile inhibitors

For clinical translation, small-molecule kinase inhibitors need to balance high anti-tumor efficacy with low systemic toxicity.^109^ Traditional single-target therapies are often hampered by the rapid development of acquired drug resistance.^110^ This resistance is typically mediated by activation of alternative bypass signaling pathways.^111^ For instance, tumors subjected to EGFR-targeted therapy frequently escape from this blockade through activation of parallel receptor tyrosine kinases like VEGFR-2 or amplification of c-Met and HER2 expression.^112^ A very successful strategy to overcome these bypass networks is the design of single low molecular weight agents with nicotinonitrile core structure, which are able to block simultaneously several complementary signaling nodes. This pharmacology multi-target is responsible for synergic therapeutic effects.^113^

This multi-target paradigm has been successfully applied to several nicotinonitrile-based candidates with encouraging pre-clinical results. Compound 151 is a classic example of a multi-kinase inhibitor targeting a number of nodes in the MAPK signaling cascade. The agent demonstrated strong inhibition activity in the nanomolar range against the wild-type EGFR (IC_50_ = 281 nM), c-MET (IC_50_ = 205 nM), BRAF (IC_50_ = 112 nM), and CDK4/6 (IC_50_ = 95 nM and 184 nM respectively). Furthermore, compound 151 showed remarkable selectivity against the mutated variants, inhibiting the mutant EGFR^T790M^ and B-RAF^V600E^ with IC_50_ values of 69 nM and 83 nM, respectively.^95^ The latter resulting in wide antiproliferative success of this agent against resistant tumor lines. Also, dual EGFR/BRAFV600E inhibitor compound 166 showed simultaneous target engagement at IC_50_ = 68 nM (EGFR) and 65 nM (BRAFV600E). This has led to compound 166 showing excellent sub-micromolar cytotoxicity (24 nM) against colorectal (HT-29) and pancreatic (Panc-1) cancer models.^98^ Furthermore, the dual c-Met/PIM-1 inhibitor compound 145 co-targeted these independent survival pathways at IC_50_ = 151 nM and 110 nM, respectively. Thus, it effectively inhibited metastatic potential in prostate and colon cancer cell lines.^25^

We further assessed the therapeutic index of these active agents by comparing cytotoxic concentrations in tumor cells to those in non-malignant human cells. The latter approach was adopted to evaluate their possible safety profiles.^114^ The typical concentration range of these investigational nicotinonitriles was depicted at low nanomolar to sub-micromolar. On the other hand, toxic thresholds within normal cells have been depicted at substantially higher concentrations. For example, compound 6 showed very selective toxicity to lung cancer cells (IC_50_ = 0.85 µM) but almost no toxicity to normal WI-38 human embryonic lung fibroblasts (IC_50_ = 59.88 µM), with an encouraging 70-fold safety margin 31. Similarly, PIM-1 inhibitor compound 115 exhibited a safe range in normal WI-38 cells (IC_50_ = 57.30 µM) in comparison to its high cytotoxic activity against PC-3 prostate cancer cells (IC_50_ = 0.99 µM) yielding a 58-fold selectivity index.^115^ In hepatospecific models, the clinical candidate 125 killed HepG2 liver cancer cells selectively (IC_50_ = 7.26 µM) without toxicity against normal human liver cells (THLE2; IC_50_ ≥ 50 µM).^6^ Finally, compound 126 showed a very good safety window of 42-fold with IC_50_ = 3.73 µM in HepG2 cells *versus* IC_50_ = 160.04 µM in WI-38 normal cells.^9^ These generous safety margins demonstrated the high structural selectivity of the nicotinonitrile scaffold for binding to the oncogenic kinase *versus* the normal cellular machinery, indicating its promising potential for future *in vivo* and clinical development.

## 4. Molecular insights into the binding and pharmacophoric adequacy of nicotinonitrile at the kinase catalytic site

Throughout the previous sections, we have presented substantial clinical evidence for the successful incorporation of the nicotinonitrile scaffold into several clinical candidates and literature-reported kinase inhibitors. Highlighting the kinases' molecular aspects and their correlated pharmacophoric features with nicotinonitrile would be beneficial for guiding future development and optimization of this scaffold. This section will discuss three main themes, including the structural features of the most described kinases with nicotinonitrile-based compounds, the key regulatory and functional elements of these kinases, and the typical architecture of their canonical catalytic site, as well as the universal kinase inhibition pharmacophore, and to what extent the nicotinonitrile moiety can be aligned with.

Among the most investigated receptor kinases as promising biotargets for nicotinonitrile-based small-molecule inhibitors are members of both the tyrosine and serine/threonine kinase families.^10^ Investigated kinases like EGFR, VEGFR-2, HER-2, c-MET, as well as CDK-2, PIM-1, and BRAF endorse remarkable structural similarities underpinning their common mechanistic catalysis of transferring ATP's γ-phosphates towards their respective substrate proteins. These targets uphold conserved protein folding despite their differential regulatory elements and cellular distributions (Fig. 5A).^116^ The catalytic domain of these proteins is bilobed, where a N-terminal lobe is compact with dominant β-sheet folding capped with αC-helix, while the carboxy lobe is typically folded as α-helices and comprises the catalytic machinery. The space between both lobes houses the central functional region of the ATP-binding site, which is highly conserved among transmembrane kinases (EGFR, VEGFR-2, HER2, c-MET) and cytosolic kinases (BRAF, PIM-1, and CDK-2).^117^

**Fig. 5:**
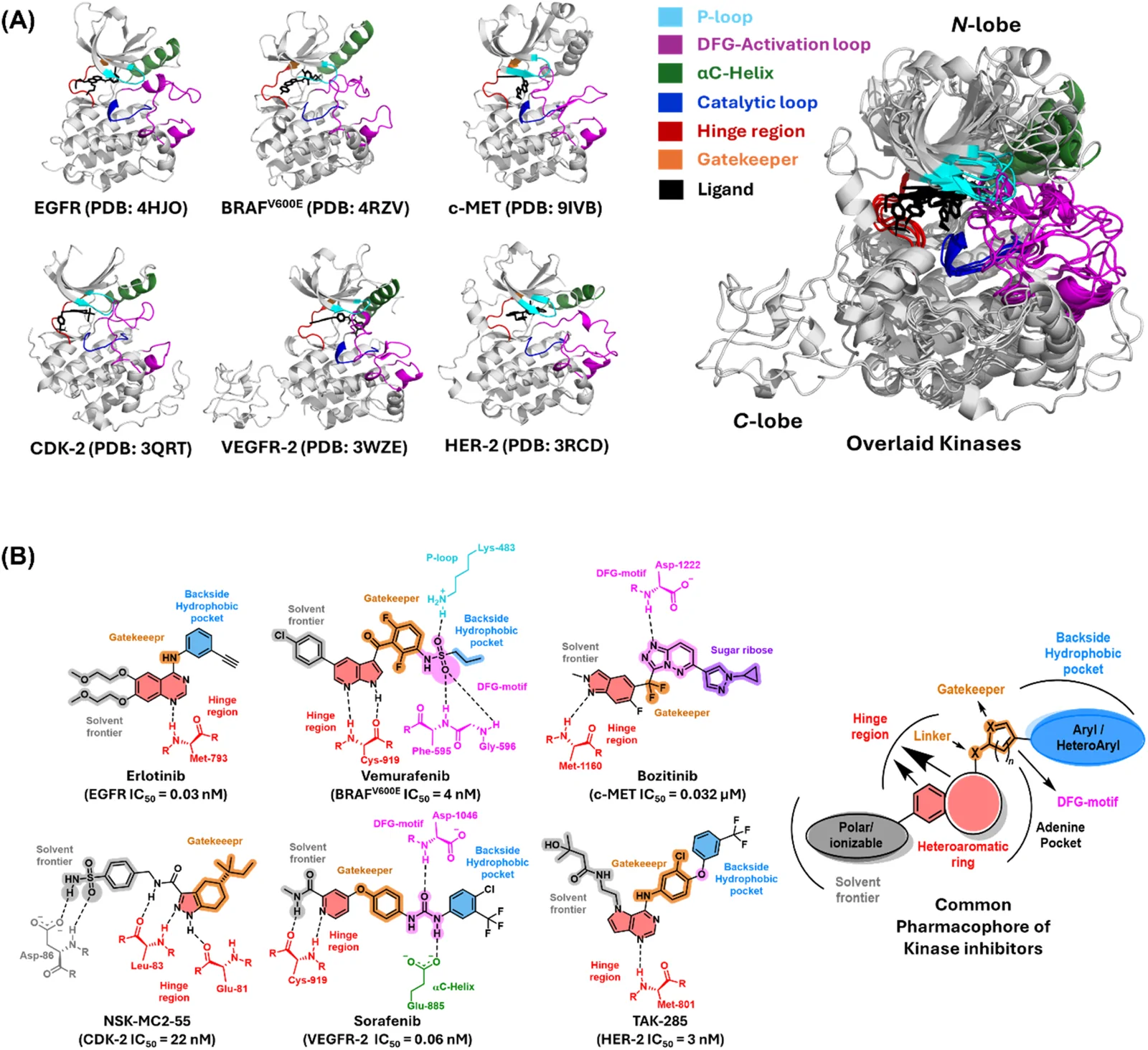
Evolutionary conservation of the kinase folding and ATP-binding site requirements for competitive kinase inhibitors. (A) 3D-cartoon representation of the highly conserved catalytic domain of different kinase family members in complex with respective co-crystallized inhibitors (black sticks). The key structural and catalytically functional elements are color-coded. (B) Common pharmacophoric features within the structure of the ATP-competitive kinase inhibitors that are complementary to the ATP-binding subsites.

Key regulatory elements within the kinase structures include the hinge region, which physically connects the catalytic domain lobes and provides a polar anchoring site for the ATP's adenine ring (Fig. 5B). Backbone hydrogen-bonding patterns (two-to-three hydrogen bonds) towards ATP and most ATP-competitive inhibitors are conserved across all kinases' hinge region residues despite their role in selectivity contribution.^118^ Another structural feature is the activation loop, starting with a conserved motif of Asp-Phe-Gly (DFG) amino acid sequence controlling the catalytically active-inactive conformational transitions as per the aspartate residue position towards Mg^+2^ for coordination. The orientation of the phenylalanine residue (in or out) has been linked to catalytic geometry disruption and access towards a deep hydrophobic back pocket often explored by type-II kinase inhibitors.^119–121^ Typical gatekeeper amino acids control the ease with which a ligand can gain access to this buried hydrophobic site. The size and identity of this gatekeeper residue are different across kinases such as VEGFR-2, EGFR, and BRAF, with a significant impact on the target-ligand selectivity and resistance interlink.^122^ The orientation of the αC-helix has also been linked with catalytic conformation shifts based on successful conserved glutamate–lysine salt bridge formation for stabilized ATP anchoring and aligned catalytic residues for transphosphorylation.^123,124^ A final recognized elementary structure is the glycine-rich phosphate-binding loop (P-loop) with its conserved GxGxxG motif, which exhibits an inherited flexibility relevant for solvent shielding, ATP's α-/β-phosphate stabilization, and conformational flexibility during ATP binding/release.^125^ All these elements are conserved despite the differential aspects in allosteric elements, phosphorylation patterns, and regulatory spines that mediate functional diversity.

Based on the conserved structural features of the kinases' ATP-binding sites, several stable common pharmacophoric characteristics (four-element blueprint) are generally presented within the inhibitors of these kinases (Fig. 5B).^126,127^ Typically, a main pharmacophoric feature is an adenine-like ring scaffold for mimicking the polar binding patterns at the kinase hinge region, providing the main assets in target selectivity.^128^ Binding at the adenine pocket can be further fortified through boosting the aromaticity of the hydrophobic core to mediate relevant van der Waals interactions. A terminal hydrophobic moiety is another pharmacophoric feature being directed towards the buried back pocket beyond the gatekeeper area. Notably, the type-II inhibitors of the c-MET, VEGFR-2, and BRAF kinases rely on this binding feature to take advantage of the bigger hydrophobic cavity being accessible in the DFG-out conformation.^121^ A flexible linker is another functional group that is strategically positioned near the activation loop DFG-motif for mediating polar contacts with DFG-aspartate or occupying the vicinal hydrophobic spaces serving as a hydrophobic channel filler stabilizing the inactive (type-II) or active (type-I) conformers.^129^ The last pharmacophoric feature is a typical polar/ionizable scaffold directed at the entrance of the ATP pocket, being exposed towards the solvent front. These decorated moieties improve the inhibitor's solubility indices, binding with residues at the pocket rim, as well as the pharmacokinetic behavior.^130^

Based on the above-described ATP-binding pocket requirements, the nicotinonitrile scaffold represents a privileged pharmacophoric core for relevant ATP-competitive inhibition (Fig. 6A). Combining heterocyclic planarity, structural versatility for synthetic derivatization, and hydrogen-bond capability, this privileged scaffold can be recognized as fundamental for adenine-pocket binders. Initially, the nicotinonitrile group mimics the adenine ring at the hinge region, capable of mediating a bidentate polar contact owing to the scaffold's electron-deficient heteroaromatic surface. The ring nitrogen and cyanide group serve as hydrogen-bond acceptor analogs of the adenine N1/N7 sites, having the cyanide with an additional dipolar-dipole or π–π stacking interaction potential for further scaffold stabilization. Compared to pyridines, the nicotinonitrile is slightly more polarized, allowing improved binding and orientation.^131^ The X-ray crystallographic structures of bosutinib^132^ and neratinib,^133,134^ representing the potent respective human HER-3 and EGFR^T790M/L858R^ mutant kinase inhibitors, depict the relevant orientation. The respective fused nicotinonitrile scaffold of these co-crystallized inhibitors showed favored anchoring at the adenine ring pocket, mediating relevant hydrogen bonding towards the backbone of the Leu771 (HER-3) or Met793 (EGFR) residues (Fig. 6B and C).

**Fig. 6:**
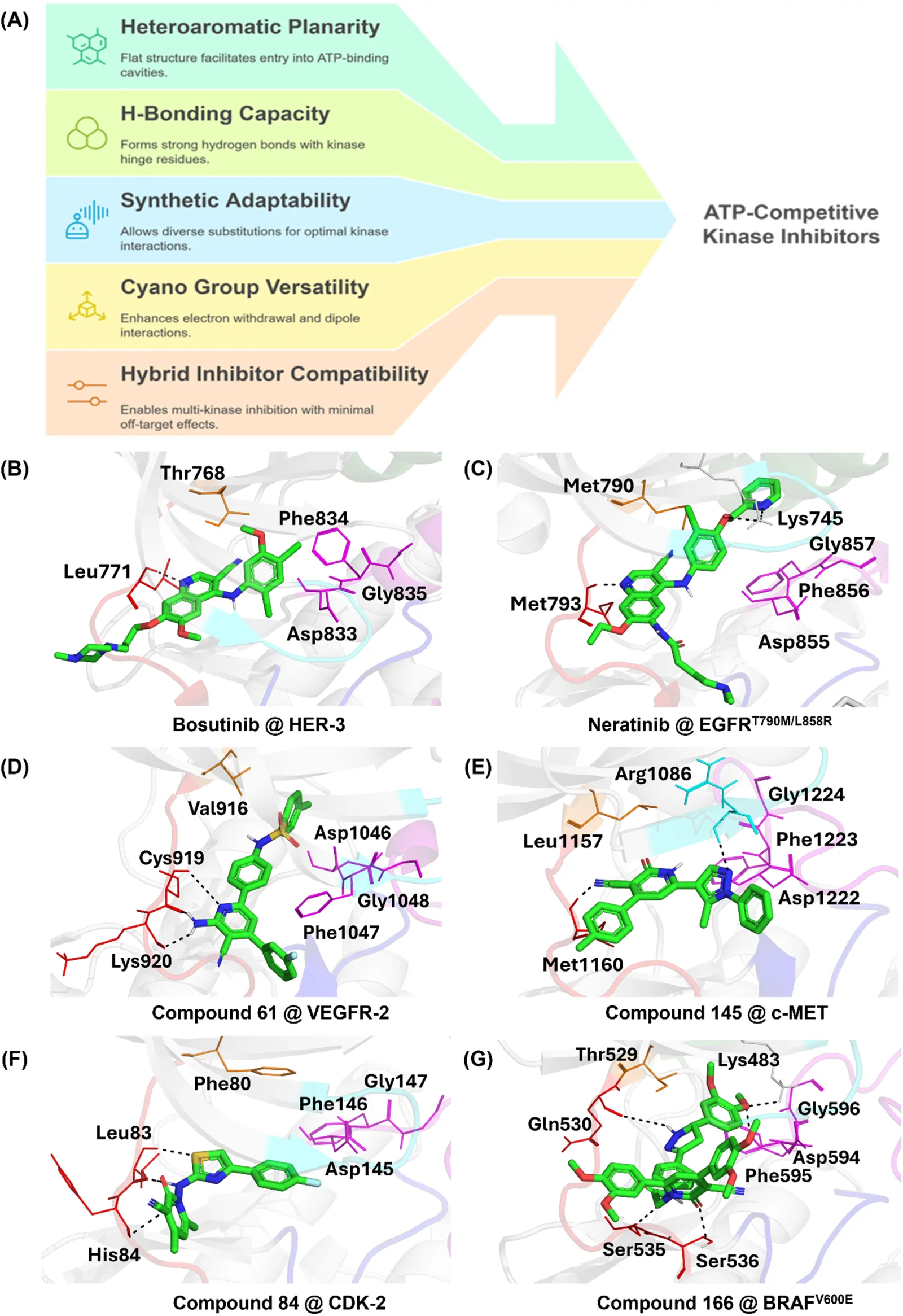
Nicotinonitrile scaffold as privileged pharmacophoric core for relevant ATP-competitive kinase inhibition. (A) Pharmacophoric features and synthetic advances of adopting nicotinonitrile scaffold as core skeleton for different kinase inhibitors. (B and C) Atomic coordinates of co-crystalline nicotinonitrile-based kinase inhibitors (bosutinib and neratinib) at HER-3 (PDB: 6OP9) and mutant EGFR (PDB: 3W2Q) catalytic sites, respectively. (D–G) Newly generated standardized illustrative molecular docking poses of representative literature-reported nicotinonitrile-based kinase inhibitors at the respective ATP-binding canonical pockets of VEGFR-2 (PDB ID: 3WZE), c-MET (PDB ID: 9IVB), CDK-2 (PDB ID: 3QRT), and BRAFV600E (PDB ID: 4RZV). Ligands are represented as green sticks, and key kinase structural elements are shown as cartoons and color-coded (red Hinge region, orange gatekeeper residue, magenta DFG activation loop, cyan P-loop, blue catalytic loop, and green αC-helix). Only residues at 4 Å from the ligand are shown as lines, labeled and colored with respect to their position. Polar interactions are illustrated as black dashed lines. PyMol v3.1.3 software was employed to illustrate and analyze compound-enzyme interactions and conformations.

It is worth mentioning that current literature lacks relevant reported co-crystallized nicotinonitrile-based inhibitors at specific significant kinase targets; VEGFR-2, c-MET, CDK-2, and BRAF. Thus, the researchers performed newly generated illustrative molecular docking simulations against the kinase domains of these selected members. This approach was highlighted important to provide a standardized, comparative visual representation of the binding modes of representative nicotinonitrile-based inhibitors. These simulations were executed purely for explanatory and comparative purposes to visually unify the pharmacophoric alignments discussed throughout this text scaffold. In brief, the protein and ligands were generated, reduced, and subsequently docked utilizing the complimentary AutoDock Vina Extended extension for the SAMSON molecular design platform, as per previous research.^135^ The binding site was defined to fit the co-crystallized ligand. The docking grid box was set to dimensions of 20 × 20 × 20 Å^3^ to include all recognized essential binding residues within the enzyme pocket. The molecular docking procedure was performed through the stochastic iterated local search/Monte Carlo optimization strategy and Broyden–Fletcher–Goldfarb–Shanno local optimization for the search of the ligand positional, orientational and conformational degrees of freedom.^136^ The docking parameters were chosen with an exhaustiveness of 100, 20 iterative binding configurations, and a maximum free-binding energy disparity of 4 Kcal mol^−1^ among the expected binding modes.^137^ The ideal anticipated conformation was selected because to high docking scores, a minimal root mean-squared deviation (RMSD) of under 2 Å from the ligand, and/or confirmed interactions with essential pocket residues aligned with small molecule inhibitors. Validity of the docking protocol and the obtained poses were ensured through redocking the co-crystallized ligands adopting the same docking protocol. Attaining a superimposed low RMSD (<2 Å) for the co-crystalized and redocked reference has been extensively documented as crucial for validating the employed docking methodology in predicting binding modes and, consequently, binding energies with possible bioactivity implications.^138–140^

Interestingly, the molecular docking investigation of different reported nicotinonitrile-based kinase inhibitors highlighted comparable orientation/confirmation across VEGFR-2, c-MET, CDK-2, and BRAF (Fig. 6D–G). The nicotinonitrile scaffold also provides adequate size, planarity, and hydrophobicity, facilitating easy access to the adenine pocket with minimal steric hindrance/penalties. The ring scaffold can be further optimized if directed toward the pocket entrance to minimize the solvation entropy associated with ligand binding. Focusing on its orientation at the center of the ATP-binding pocket, the nicotinonitrile scaffold offers structural diversity for occupying additional ATP subsites by extending substitutions towards the solvent frontier, the type-II inhibitor's selectivity (gatekeeper-adjacent, buried, hydrophobic pocket), the DFG-motif/vicinal hydrophobic channel, and the ribose-anchoring pocket. This can be leveraged by using the cyanide group as a point of bioisosteric replacement or by synthetic derivatization into amidines, amides, and heterocyclic and fused systems.^141,142^ All these aspects highlight the nicotinonitrile scaffold as an excellent platform for structure-guided optimization across diverse kinase families.

## 5. Comprehensive structure–activity relationship analysis and design limitations

The present review of the different nicotinonitrile-based compounds enables a comparative analysis that evidences the structure–activity relationships (SAR) governing the biochemical kinase inhibition and the cellular cytotoxicity directly. The central, electron deficient 3-cyanopyridine core is the basic pharmacophoric requirement for this class of compounds. This planar heteroaromatic nucleus is a very good adenine mimic, enabling the essential bidentate polar anchoring in the ATP binding hinge region. In particular, the heterocyclic ring nitrogen and the adjacent nitrile group serve as hydrogen bond acceptors, mimicking the N1/N7 positions of adenine. Furthermore, the highly polarized nitrile group adds to dipole–dipole and π–π stacking interactions, which stabilize the ligand in the hinge pocket of enzymes such as the residue backbones of EGFR (Met793) and HER-3 (Leu771).

In addition to this basic anchoring mechanism, the type and position of substituents at the C2, C4, and C6 positions of the pyridine ring are the primary determinants of cellular cytotoxicity. Very potent cytotoxicity is associated with the incorporation of flexible, polar or ionizable decorators at C-2 position. Thus, the introduction of a flexible hydrazide linker (compound 6, A549) (IC_50_ = 0.85 µM)^31^ or an acetohydrazide extension (compound 103, HepG2) (IC_50_ = 0.28 µM)^143^ enables the molecule to reach the highly dynamic solvent frontier at the pocket rim. This projection minimizes solvation entropy and results in better drug-like solubility. Importantly, the authors commented on the importance of transforming 2-pyridone intermediates into fully aromatic *O*-alkylated systems (*e.g. O*-alkylated quinoline–pyridine hybrids 142a, 142b).^93^ The latter dramatically enhanced both enzymatic inhibition and nanomolar cell-line cytotoxicity, relative to their parent pyridones. This gain in potency highlights the role of increased core aromaticity and structural rigidity in supporting target engagement. Meanwhile, the hydrophobicity of substituents at the C4 and C6 positions determines the ability of the molecule to occupy the buried hydrophobic subsites and back-pockets near the gatekeeper region. There is a strong correlation exceptional oncotoxic potency with the introduction of extended, electron-rich aromatic systems at these positions. The bulky triphenylamine linked pyridine compound 179 showed excellent IC_50_ = 0.00803 µM against A-549 cells,^56^ while as the highly effective multi-target MAPK pathway inhibition shown by the 6-indolylpyridone derivative compound 151 (ref. 48) were both excellent examples. These bulky hydrophobic groups fill the neighboring hydrophobic pockets, stabilizing the inactive conformation of the transmembrane kinases.

In contrast, analysis of scaffolds with low potency or poor translation from biochemical assays to cellular models highlights critical design limitations. The most serious structural failure was substituting the C4 and C6 positions with small, non-optimized aliphatic substituents. In compounds 63 and 65, the presence of simple methyl groups at C4 and C6 leads to a total lack of access to the deeper hydrophobic back-pockets of CDK2. These molecules displayed moderate inhibition of cell-free enzyme (IC_50_ = 0.24 µM and IC_50_ = 0.50 µM).^48^ Nonetheless, both latter were characterized by very weak antiproliferative profiles in cell lines, with IC_50_ values ranging from 56.9 µM to 67.2 µM. Likewise, the absence of flexible, polar solvent-front decorators or the presence of rigid, non-optimized heterocyclic extensions (*e.g.* the simple thienyl substitution in compound 189) severely hampered cellular uptake and target-binding, resulting in weak micromolar cytotoxicity (IC_50_ = 19.85 µM).^60^ These comparative trends illustrate that a polarized 3-cyanopyridine core is required for hinge binding but that a balanced structural architecture with a C2 solvent-front decorator in combination with bulky, pocket-filling hydrophobic substituents at C4 or C6 is necessary to elicit meaningful cellular cytotoxicity. The above-described SAR insights are summarized in Fig. 7.

**Fig. 7:**
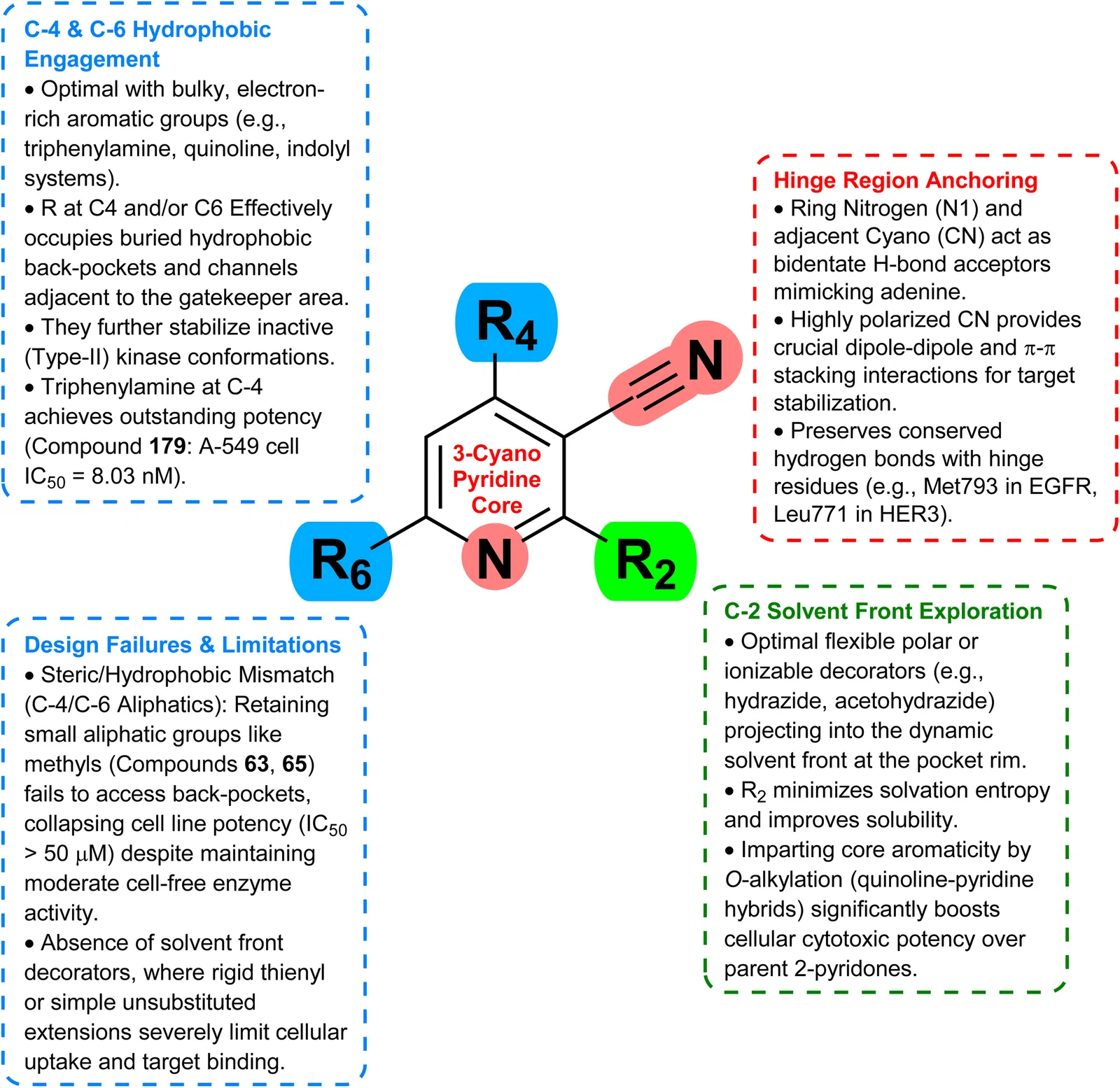
Structural blueprint and SAR landscape of the 3-cyanopyridine (nicotinonitrile) scaffold in kinase-directed drug design. Schematic diagram summarizes the major pharmacophoric determinants of cellular cytotoxicity and enzymatic potency and the critical structural pitfalls that result in reduced therapeutic efficacy.

## 6. Conclusion

Nicotinonitrile-containing derivatives represent a structurally versatile class of compounds that has attracted considerable interest in anticancer drug discovery. Across the studies reviewed, several derivatives demonstrated potent biochemical inhibition of cancer-associated kinases, including EGFR, VEGFR-2, HER2, CDKs, PIM kinases, c-Met, and BRAF, together with antiproliferative activity in a range of cancer cell models. In selected studies, these effects were accompanied by mechanistic evidence such as apoptosis induction, cell-cycle perturbation, or modulation of oncogenic signaling pathways. However, these findings should largely be regarded as preclinical, since biochemical potency and cell-line cytotoxicity do not by themselves establish therapeutic efficacy or safety. Despite encouraging activity profiles, important translational gaps remain. For many investigational nicotinonitrile derivatives, ADME and pharmacokinetic properties have not been comprehensively characterized, while *in vivo* antitumor efficacy and systemic toxicity have been evaluated only in a limited number of studies. In addition, broader kinase selectivity, potential off-target interactions, therapeutic index, and resistance mechanisms require more systematic investigation. Because the ATP-binding regions of many kinases are highly conserved, achieving adequate target selectivity remains a particular challenge for this class of inhibitors. Future development should therefore integrate biochemical profiling with cellular target-engagement studies, pharmacokinetic and toxicological assessment, appropriate *in vivo* tumor models, and mechanistically relevant drug-resistance systems. Taken together, the nicotinonitrile scaffold remains a promising platform for designing single-, dual-, and multi-target anticancer agents. Its future translational value will depend not only on potency but also on demonstrated selectivity, pharmacokinetic suitability, tolerability, *in vivo* efficacy, and robust validation of mechanisms relevant to tumor heterogeneity and acquired resistance.

## 7. Expert opinion

Strategically integrating nicotinonitrile groups into anticancer drug design provides a basis for developing multitarget, selective therapeutics. Its strong binding to kinase active sites, combined with its tunable electronic and steric properties, should make it especially useful for treating complex, resistance-prone cancers. The development of dual-acting and multi-targeted derivatives, on the other hand, highlights a trend in oncology toward breaking away from like-with-like (network-on-node to network-based) pharmacology rather than single-point inhibition. Despite encouraging biochemical and *in vitro* findings, further translational development is limited by incomplete characterization of ADME and pharmacokinetic properties, toxicity, kinase selectivity, off-target effects, and resistance mechanisms, as well as relatively limited *in vivo* validation. Lead compounds can be further optimized through rational design based on SAR and *in silico* docking. With their synthetic accessibility and broad bioactivity, nicotinonitriles will play an important role in the new paradigm for kinase inhibitors. Their further development in the context of integrative oncology (*i.e.*, genomics, proteomics, and systems biology) may lead to new personalized therapeutic approaches for cancer patients.

## Conflicts of interest

There are no conflicts to declare.

## Data Availability

No primary research results, software or code have been included and no new data were generated or analysed as part of this review.
